# The temporal organization of mouse ultrasonic vocalizations

**DOI:** 10.1371/journal.pone.0199929

**Published:** 2018-10-30

**Authors:** Gregg A. Castellucci, Daniel Calbick, David McCormick

**Affiliations:** 1 Neuroscience Institute, New York University School of Medicine, New York, NY, United States of America; 2 Haskins Laboratories, New Haven, CT, United States of America; 3 Department of Genetics, Yale University of Medicine, New Haven, CT, United States of America; 4 Institute of Neuroscience, University of Oregon, Eugene, OR, United States of America; 5 Department of Biology, University of Oregon, Eugene, OR, United States of America; University of Missouri Columbia, UNITED STATES

## Abstract

House mice, like many tetrapods, produce multielement calls consisting of individual vocalizations repeated in rhythmic series. In this study, we examine the multielement ultrasonic vocalizations (USVs) of adult male C57Bl/6J mice and specifically assess their temporal properties and organization. We found that male mice produce two classes of USVs which display unique temporal features and arise from discrete respiratory patterns. We also observed that nearly all USVs were produced in repetitive series exhibiting a hierarchical organization and a stereotyped rhythmic structure. Furthermore, series rhythmicity alone was determined to be sufficient for the mathematical discrimination of USVs produced by adult males, adult females, and pups, underscoring the known importance of call timing in USV perception. Finally, the gross spectrotemporal features of male USVs were found to develop continuously from birth and stabilize by P50, suggesting that USV production in infants and adults relies on common biological mechanisms. In conclusion, we demonstrate that the temporal organization of multielement mouse USVs is both stable and informative, and we propose that call timing be explicitly assessed when examining mouse USV production. Furthermore, this is the first report of putative USV classes arising from distinct articulatory patterns in mice, and is the first to empirically define multielement USV series and provide a detailed description of their temporal structure and development. This study therefore represents an important point of reference for the analysis of mouse USVs, a commonly used metric of social behavior in mouse models of human disease, and furthers the understanding of vocalization production in an accessible mammalian species.

## Introduction

Wild and laboratory house mice (*Mus musculus*) produce a variety of ultrasonic vocalizations (USVs), including neonatal isolation calls [1–3], female social investigation calls [4–7], female pup separation calls [8, 9], male aggressive calls [10], and male courtship vocalizations [11–14]. Unlike most mouse audible distress calls [5, 10, 14–17] (though *c.f*. [18, 19]), individual USVs are preferentially produced in repetitive series [20] which display a regular temporal structure [21, 22].

Rhythmic multielement calls, such as mouse USV series, are common in the vocalization repertoires of a wide variety of animals, including rodents (*Baiomys* [23], *Meriones* [24], *Peromyscus* [25], *Rattus* [26], *Scotinomys* [27], *Spermophilus* [28], *Tamiasciuris* [29]), bats (*Myotis* [30], *Pteronotus* [31], *Rhinolophus* [32], *Saccopteryx* [33]), amphibians (*Hyla* [34–36]), ungulates (*Dama* [37]), pinnipeds (*Halichoerus* [38], *Leptonychotes* [39, 40], *Mirounga* [41, 42], *Neophoca* [43], *Odobenus* [44], *Phagophilus* [45, 46], *Zalophus* [47, 48]), cetaceans (*Balaenoptera* [49, 50], *Physeter* [51–53]), monkeys (*Cercophithecus* [54]), and a wide variety of avian species (*Aptenodytes* [55, 56], *Charadrius* [57], *Eupsittula* [59], *Lampornis* [60], *Morus* [58], *Poecile* [61], *Taeniopygia* [62]). These vocalizations are produced with a high degree of temporal regularity, though the acoustic properties of the individual elements may vary considerably (*i.e*. heterotypic calls). For example, the song of male zebra finches (*Taeniopygia guttata*) consists of multiple acoustically distinct notes whose onsets are timed to a near-isochronous rhythm [62]. Alternatively, in many species the individual vocal elements are nearly identical acoustically and also produced in temporally regular series (*i.e*. homotypic calls), as is observed in the advertisement calls of Pacific treefrogs (*Hyla regilla* [35]), the songs of Alston’s brown mouse (*Scotinomys teguina* [23, 27]), and the 20 Hz pulses of fin whales (*Balaenoptera physalus* [50]). Furthermore, in some species the rhythmic features of multielement calls vary systematically with the state of producer. For instance, male California sea lions (*Zalophus californianus*) produce long series of nearly identical barks, but the specific repetition rate of the barks reflects the ongoing behavior of the animal [47, 48]. Similarly, the repetition rate of fallow deer (*Dama dama*) groans is related to which breeding-associated behavior the buck is currently engaged in [37].

In many cases, the temporal features of multielement calls are informative in the identification of conspecifics. For example, various species of *Hyla* treefrogs produce advertisement calls which vary in both repetition rate and the dominant frequency of individual call elements. However, species recognition by female treefrogs is completely dependent on call repetition rate alone, and not element frequency [36]. Similarly, harp seals (*Pagophilus groenlandicus*) and Weddell seals (*Leptonychotes weddellii*) produce multielement calls in three and seven stereotyped rhythmic patterns, respectively, which are temporally distinct from both abiotic environmental noise as well as the calls of other pinnipeds which inhabit the same environment [39, 45, 63]. The codas of sperm whales (*Physeter macrocephalus*) and pulsed threat vocalizations of Northern elephant seals (*Mirounga angustirostris*) also differ in their temporal structure from subpopulation to subpopulation [41, 53], suggesting that the timing of these calls contains information regarding group identity. The gross temporal features of multielement calls may also relay information regarding the sex of the producer, as is the case for Alston’s brown mouse, where the primary distinction between the songs of males and females is multielement call duration [23]. Furthermore, multielement calls within the same species may be differentiated by their temporal structure. For example, the leopard alarm calls of Diana monkeys (*Cercopithecus diana*) are shorter, contain less elements, and have longer silent intervals between elements in comparison to eagle alarm calls, though the elements of the two calls display many similar spectral features [54]. Similarly, the multielement encounter and advertisement calls of the Pacific treefrog are nearly identical in spectral structure, but differ dramatically in their temporal organization [34, 35].

The exact motivations for producing multielement calls over calls consisting of a single element are unknown, however it is thought that the production of temporally regular repetitions of a given call would increase its probability of identification, as only a portion of the redundant signal must be perceived for successful detection [39, 45, 47, 55, 62, 64]. Furthermore, temporal regularity improves auditory detection [65–67] and auditory discrimination [68], adding additional robustness to the perceptibility of multielement vocalizations. Therefore, the use of these calls is thought to be especially advantageous for animals vocalizing in noisy environments [55, 64]. This hypothesis is supported by the fact that many of the animals observed to produce multielement calls must contend with high levels of environmental noise from abiotic sources or calling conspecifics; for example, king penguins (*Aptenodytes patagonicus*) must produce vocalizations that can be heard over abiotic noises resulting from high wind speeds [55], and harp seals must compete with thousands of calling conspecifics during breeding season [69]. Furthermore, both king penguins and harp seals have been observed to increase the duration of their multielement calls in response to increased environmental noise [55, 64], suggesting that these species produce increased repetitions of their calls as an anti-masking strategy.

Given the suitability of multielement calls for effective vocal communication in noisy environments, the ecology of the house mouse provides a context in which the production of repetitive USV series is preferable to individual USVs. Specifically, mice form high density colonies in close proximity to humans, with male mice maintaining bordering territories populated by several breeding females, their offspring, and subordinate males [70]. As males, females, and mouse pups are all known to produce USVs in many behavioral contexts, these environmental conditions would undoubtedly result in numerous calling conspecifics, and consequently a high level of background noise. Therefore, the production of repetitive, long duration USV series would be beneficial to the perception and discrimination of the different USV types, which is known to occur in at least female mice, who approach pup and male USVs [71–75] but not female USVs [76].

Furthermore, the temporal structure of mouse vocalizations is known to be important for their perception, while the role of spectral structure is less clear. For example, playback of synthetic pup calls exhibiting naturalistic spectrotemporal features elicits robust approach behavior from lactating female mice, however playback of spectrally appropriate synthetic USVs with distorted temporal structure fails to elicit this behavior [77]. Likewise, lactating female mice approach ultrasonic white noise and tone bursts at rates indistinguishable from playback of actual pup isolation USVs as long as the synthetic sounds maintain the temporal organization of the natural calls (tone bursts [78]; bandpass noise [22]). Similarly, maternal behavior is elicited from lactating female mice in response to audible pup wriggling calls [79] as well as a series of three-harmonic stacks exhibiting a temporal configuration comparable to natural calls [19]. In summary, these studies demonstrate that most to nearly all of the spectral structure can be removed from pup isolation USVs and wriggling calls, but as long as series temporal structure is maintained, their perception is not measurably affected. Therefore, the temporal organization of mouse vocal series as a whole appears to be important for perception, rather than the fine acoustic structure of individual vocal elements.

Despite the rich bioacoustics literature on multielement calls, the relevant ecology of the mouse, and the known importance of call timing in the perception of mouse USVs, the detailed temporal structure of mouse USV series has not been examined. In this study, we present such an analysis of adult male courtship USVs, and specifically demonstrate the stability of USV temporal organization while also illustrating its intrinsic variability across individuals. Additionally, we show that temporally distinct USV classes arise from separate articulatory patterns, and provide evidence for hierarchical groupings of USVs within multielement series. We also describe the development of USV production in a cohort of male mice from birth into adulthood, and show that USV spectrotemporal structure develops continuously from birth. Finally, we compare the temporal properties of pup USVs, adult male USVs, and adult female USVs, and demonstrate that vocalization timing alone is sufficient to discriminate between these three classes of USVs.

## Materials and methods

### Animals

Nineteen male and 15 female C57Bl/6J (strain #000664; B6 henceforth) mice were used as subjects for this study. The female mice were ordered from Jackson Laboratories (Bar Harbor, USA) as adults (4+ months of age) and were housed in groups of four. Eight of the 19 male mice were ordered from Jackson Laboratories and arrived before P25, and the other 11 mice were born at Yale to dams ordered from Jackson Laboratories. All male mice were individually housed from weaning (P21) or their arrival at Yale, as we have previously observed that group-housed males vocalize less consistently than singly housed males. However, male mice were regularly socialized with female mice as described in the following sections. All mice were housed in standard caging (approximately 11.5 × 7 inches, and 5 inches high) and kept in a reverse day-night cycled room in order to match the active period of the mouse to that of the human experimenters. Following the completion of this study, animals were retired to breeding and/or used for additional experiments in the laboratory.

All procedures used in this study were approved by the Institutional Animal Care and Use Committee of Yale University, and were carried out in accordance with relevant guidelines and regulations. All procedures performed for this study caused no pain to the subject animals, as only social vocal behavior was investigated. Mice were regularly handled by experienced experimenters in order to minimize the distress of the animals during handling and testing.

### Vocalization recording

All recordings were performed in a sound-attenuated chamber (Industrial Acoustics, New York, USA) using an ultrasonic microphone (CM16/CMPA, Avisoft Bioacoustics, Berlin, Germany). During recording sessions, acoustic data were sampled at 250 kHz and digitized using an Avisoft signal conditioner and recorded with the Avisoft RECORDER software. Between sessions, the recording apparatus containing the mice was cleaned with 70% aqueous ethanol, rinsed with water, and dried.

#### Pups

To record neontal isolation calls, an individual mouse pup was placed in cylinder made of non-absorbent sound attenuating rubber. The cylinder was approximately 7 inches in diameter and 6 inches high. The cylinder was then placed under the microphone and the pup was allowed to freely move and vocalize for 3-5 minutes. Pups were recorded once a day until P16; seven pups began recording at P1 and four at P0. Pups were identified with distinctive marks on their tails made with a nontoxic permanent marker.

#### Females

Adult female mice were recorded either in isolation or while paired with another adult female mouse, and were found to vocalize for a brief period in both contexts. In either case, a female mouse was placed in a rectangular recording apparatus (approximately 11.5 × 7 inches, and 5 inches high) lined with non-absorbent rubber matting to dampen the sound of footfalls and facilitate cleaning between sessions, and placed under the microphone. When the subject female was paired with another female mouse, both mice were allowed to interact and vocalize freely, otherwise the single subject mouse was allowed to move freely and vocalize in isolation. Each female mouse was recorded 6-9 times over a 30 day period, and mice were recorded no more than twice a day.

In female dyads, it was not possible to determine which of two mice were vocalizing. However, we inspected all recordings to determine whether the animals were vocalizing at the same time. We observed exceedingly few cases of this, consistent with prior studies suggesting that one female of the dyad is responsible for the production of most observed USVs [10]. Furthermore, we found that the temporal properties of female USVs produced in dyads were similar to those produced in isolation (see Results and S10 Fig for additional information), which further suggests the female mice were not vocalizing simultaneously at a high rates.

#### Males

The eight male mice ordered from Jackson Laboratories began vocalization recording sessions at P25 and were recorded approximately three times a week until P95. The 11 mice who also had their pup vocalizations recorded were similarly tested three times a week, but began at P17 or P18 and ended at P85. As with the female mice, an individual male mouse was placed in the rectangular recording apparatus with a random female, and allowed to interact with the female mouse and vocalize freely for approximately three minutes.

We previously determined that regular socialization of male mice from a young age is critical for the development of consistent vocal behavior in adulthood [21]. In agreement with our previous findings, we observed that the male mice used in this study all stably produced high call rates of >90 USVs per minute upon reaching adulthood (P50) (S1 Fig).

Female mice are known to produce USVs when paired with male mice, but at rates much lower than male mice [4, 5, 13, 17, 20, 80–82]. As reported in Castellucci *et al*. (2016), thorough inspection of the recordings used for this study revealed few instances where female and male mice vocalized at the same time [21]. Therefore, consistent with previous reports, the vast majority of the vocalizations detected in male-female dyads indeed appeared to be produced by the male.

### USV detection

#### Adult male and female USVs

Vocalization detection and analysis was conducted using a custom Matlab (Mathworks, Natick, USA) script described previously in detail [21]. In brief, acoustic recordings were first bandpass filtered from 30 to 120 kHz using a zero phase delay finite impulse response equiripple filter. Spectral flatness was then calculated in 256 point bins across the entire recording with no overlap. Periods with a spectral flatness value less than 0.6 separated by less than 40 ms were grouped together into a potential single USV; these criteria were empirically determined in Castellucci *et al*. (2016) [21]. Furthermore, rodents produce one USV per sniff cycle [20, 83, 84], and in order to correctly delineate mouse USVs in this fashion, a minimum silent intervocalization interval (IVI) of 40 ms is required; lower values erroneously divide USVs occurring on the same exhalation [20].

Dominant frequency was then calculated in 512 point bins with 50% overlap across the potential USVs to estimate spectral shape. In order to reject periods of noise contaminating the potential USVs, intervals with unstable dominant frequency (changing by >10 kHz twice in less than 4 ms) were rejected; this algorithm defined our minimum USV duration as 3 ms. The resulting durations of stable dominant frequency and low spectral flatness were considered putative USVs. Finally, as some low frequency noise would occasionally remain in the passband after filtering, putative USVs with an overall dominant frequency less than 39 kHz were rejected.

#### Pup USVs

Pup isolation USVs were detected in a similar manner to adult USVs, except the recordings were bandpass filtered from 40 to 120 kHz, and putative USVs with a dominant frequency less than 45 kHz were rejected. These higher thresholds were used because pups produce loud audible clicks in addition to USVs [85], and these clicks often have energy in the lower ultrasonic frequency range (20-50 kHz). Finally, we empirically set a minimum IVI for pups by examining the distribution of silent periods in all recordings of pups greater than 8 ms in duration. Consistent with the similar analysis performed for adult male mice in Castellucci *et al*. (2016), we found that IVIs separating USVs occurring on separate exhalations formed a distinct distribution from the periods of silence occurring within USVs (see Results and S8 Fig for additional information), and therefore defined the minimum IVI value as lowest point in the trough between the two distributions [21]. In order to well characterize the distribution of these silent periods, we grouped together several consecutive days of recordings such that each age grouping had a sufficient and comparable number of data points.

### Vocalization analyses

#### USV duration and IVI distribution fits, and related metrics

All Gaussian fits were performed using Matlab’s ‘gmdistribution.fit’ function with a maximum of 500 iterations. USV durations greater than 300 ms were rejected to exclude outliers. The bimodality of the USV duration distributions were assessed using Ashman’s D Score [86], which was calculated using the following:

$$\begin{array}{c} D=2^{\frac{1}{2}}\times \frac{|\mu_{1}-\mu_{2}|}{\sqrt{\sigma_{1}^{2}+\sigma_{2}^{2}}} \end{array}$$

where *μ* and *σ* are the center and standard deviation of each Gaussian, respectively. Cutoff durations between short and long USVs were defined as the intercept of the two Gaussian fits to the USV duration distribution to the nearest millisecond.

Maximum IVI durations used in the analyses presented in Figs 7 and 8 were calculated by computing the IVI distribution in 10 ms bins, then finding the width at 75% of the maximum peak of the distribution and taking the upper limit of the width as the maximum IVI duration. These values were within 20 ms of the maximum IVI values defined by the analyses of respiratory behavior during USV production presented in Fig 3.

#### Normalized long USV production probabilities

In Fig 3 and S3 Fig, the probability of long USV production as function of preceding and following IVI duration was calculated for each mouse. These probabilities were then normalized within individual mice by setting the minimum value to 0 and the maximum to 1, and finally the average normalized probability for each IVI duration was computed across mice.

#### Conditional transition probabilities and preference scores

Transition probabilities between USV types was assessed with raw conditional probabilities and Preference Scores. The calculation and rationale for using Preference Scores is described in detail in Castellucci *et al*. (2016) [21]. Briefly, Preference Scores allow for a normalization of conditional probabilities across mice who produce differing numbers of short and long USVs, and therefore would be expected to produce certain transitions more often than other mice simply due to differences in inventory. A Score of 1 indicates an absolute rule that is not violated, while a Score of 0 indicates that a transition occurs at chance levels. Transitions that are dispreferred have negative Preference Scores by convention. Preference Score is calculate with the following:

$$\begin{array}{c} PreferenceScore_{preferred}=\frac{P(X|Y)-P\left(X\right)}{1-P\left(X\right)} \end{array}$$

$$\begin{array}{c} PreferenceScore_{dispreferred}=\frac{\left|P(X|Y)-P\left(X\right)\right|}{0-P\left(X\right)} \end{array}$$

Where P(X|Y) is the conditional probability of observing X given Y, and P(X) is the probability of observing X at random.

#### USV categorization

A linear classifier was designed for categorizing groups (series) of USVs (USV series defined in Fig 3 as occurring on consecutive, uninterrupted vocal sniffs) as produced by either a mouse pup, adult female mouse, or adult male mouse using three temporal criteria only: USV duration, call interval, and duty cycle. If a 75% of the USVs in a group had a call interval less than 130 ms, a duration less than 100 ms, and a duty cycle less than 50%, the group was categorized as a female call. If all the same criteria were met but 75% of the USVs had a call interval greater than 130 ms, the group was categorized as a pup call. All other groups were categorized as male calls. Individual criterion values were set empirically based on the analysis presented in Fig 8b.

As USV groups are not usually produced in isolation, we also tested the performance of the classifier if more than a single group was considered. In this situation, the classifier would categorize a set number of randomly selected groups of a single call type, then make an overall decision on the set based on which call type had the highest number of categorizations overall. If a tie between types was observed for a set, the classifier would make a random choice between the types with the highest number of categorizations.

#### Age groupings

For the analyses presented in Figs 1–7 and S1–S6 and S11 Figs, only data from male mice aged P50 to P85 were used. For the analysis in Fig 7 and S7–S9 Figs, the relevant age groups are denoted on the respective figures. Finally, all pup data, all adult female data, and all adult male data (P50-P95) were used for the classifier analysis in Fig 8 and S10 Fig.

### Respiratory recordings

#### Plethysmography

Vocalizations were recorded along with simultaneous respiratory recordings using freely moving plethysmography. Specifically, a single male mouse was placed in rectangular clear plastic chamber with a tight-fitting lid, which was itself inside of a shielded sound attenuated chamber (Gretch-Ken Industries, Lakeview, USA). The lid had a microphone jack embedded in it so the ultrasonic microphone could be placed above the mouse for vocalization recording. From the side of the chamber, a small rubber tube connected the interior of the chamber to a differential pressure transducer (Data Sciences International, Saint Paul, USA), with the room pressure serving as the reference pressure. The signal from the transducer was subsequently amplified with a single channel differential amplifier (A-M Systems, Carlsborg, USA) at 5,000x and bandpass filtered online from 1 to 100 Hz with a 2^nd^ order Butterworth filter. The amplified, filtered signal was digitized and sampled at 1 kHz using a Micro 1401 and Spike2 acquisition software (Cambridge Electronic Design Limited, Cambridge, UK). Following acquisition, the signal was again bandpass filtered from 1 to 20 Hz with a zero phase delay infinite impulse response Butterworth filter.

#### Vocalization elicitation

To elicit vocalizations from the 11 adult male mice used as subjects for the analysis of respiratory activity during vocalization (the same adult male mice who had their pup vocalizations recorded; who were older than P85 at the time of respiratory recordings), soiled female bedding was placed into the chamber with the subject mouse. The male subject mouse was then allowed to move around the chamber freely and interact with the bedding, and any resulting vocalizations were recorded with the ultrasonic microphone.

#### Respiratory analysis

To identify periods of active sniffing, we used a detection algorithm that relied on kinematic landmarks rather than local pressure minima and maxima. We found that active sniffs had a characteristic kinematic trajectory—specifically a local positive-going acceleration maximum, followed by a local positive-going velocity maximum, followed by a local negative-going acceleration maximum, then a local negative-going velocity maximum, followed finally by a local positive-going acceleration maximum. The onset of active inhalation was defined as the first positive-going acceleration maximum, and the onset of active exhalation was defined as the negative-going acceleration maximum. To reject any minute fluctuations in pressure, we discarded any potential sniffs that did not exceed an empirically defined threshold. We found this detection routine identified sniffs with a low error rate while also being robust to slow pressure fluctuations in the chamber.

The vocalization recordings were aligned to the pressure recordings using two syncing pulses, one at the beginning and one at the end of the recording, that were output from Spike2 to a buzzer that produced a broadband audible noise. The timing of individual USVs were then matched to the closest sniffs. Any potential sniffs occurring during a USV were rejected, and USVs occurring on the same sniff were joined into a single USV. Any USVs which started before an exhalation onset or ended after an inhalation onset were rejected as mislabellings and excluded from analysis. Finally, as the mice were actively interacting with bedding, some broadband noise would occasionally remain in the passband after bandpass filtering from 50-120 kHz. To ensure USV detection was not disrupted by this noise, and we visually inspected all detected USVs, and rejected periods of noise erroneously labelled as part of a USV or as an entire USV. USVs that were erroneously grouped as a single USV due to intervening periods of noise or an IVI slightly below 40 ms were likewise corrected.

### Statistical analyses

All statistical analyses were performed in Matlab or Graphpad Prism (La Jolla, USA). Data sets were tested for normality using D’Agostino-Pearson tests, and statistical significance was assessed with the appropriate parametric and non-parametric tests as indicated in **Results** and relevant figure legends. Summary statistics and statistical details for tests are presented in the S1–S32 Tables.

## Results

### USV and silent interval production

#### USV classes

**Fig 1:**
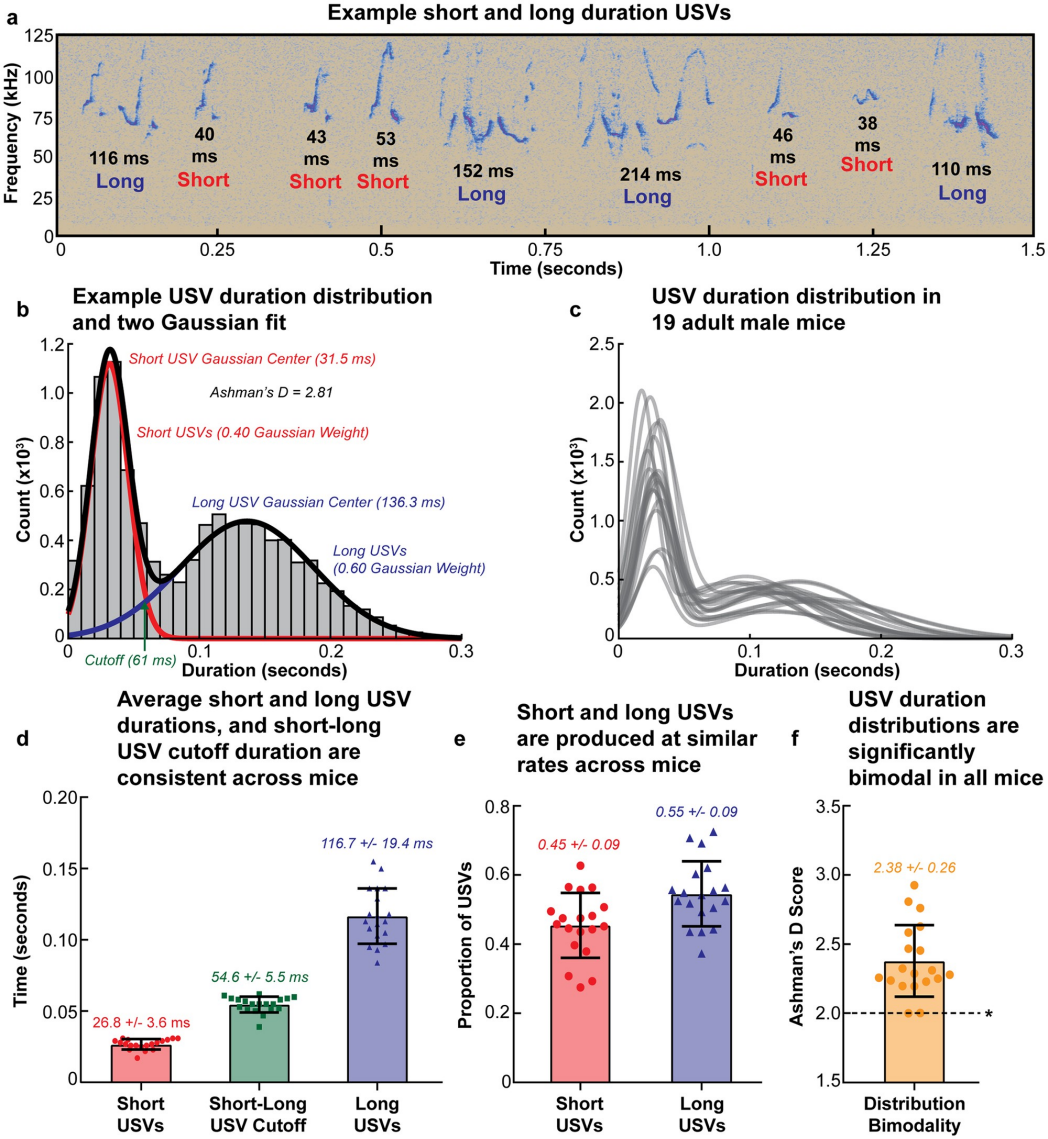
C57Bl/6J mice produce temporally distinct USV classes. **(a)** Example spectrogram showing short and long USVs in adult male ultrasonic courtship vocalizations. **(b)** Example histogram of USV duration for one mouse with the fit of two Gaussians overlaid. Also labelled are the Gaussian centers and Gaussian weights, the intercept of the two Gaussian distributions representing the cutoff between short and long USVs in the animal, and the Ashman’s D score for the distribution. **(c)** The sum of the fit of two Gaussians to the USV duration distributions from all 19 adult mice. **(d)** The average Gaussian centers from the duration distributions of short and long USVs in all 19 mice, as well as the cutoff values between the two distributions. **(e)** The average proportions of short and long USVs produced by all 19 mice. **(f)** The Ashman’s D scores for each of the 19 animals’ USV duration distributions; a score of 2 or greater represents significant separation between two distributions. In (d-f), error bars indicate the mean across mice +/-1 standard deviation, and values for individual mice are represented by the 19 overlaid points. Summary statistics for (d-f) are presented in S1 Table.

As first reported in Castellucci *et al*. (2016), we observed that adult male B6 mice produced two temporally distinct classes of courtship USVs: short duration USVs and long duration USVs [21], and that the USV duration distribution of each of the 19 mice examined was well fit by the sum of two Gaussians (Fig 1a–1c). Performing these fits allowed the average short and long USV durations, cutoff duration between the two USV types, and proportion of each USV type produced to be calculated for each mouse and the cohort as a whole. Short and long USVs were found to be 26.8 +/- 3.6 ms (mean +/- one standard deviation here and onwards) and 116.7 +/- 19.4 ms in average duration, respectively (Fig 1d), and mice produced roughly equal proportions of short and long USVs (Fig 1e). The mean cutoff duration between short and long USVs was found to be 54.6 +/- 5.5 ms, but for all subsequent analyses, the cutoff values for individual mice were used to examine each mouse’s respective data. Finally, we found that each mouse had significant separation between the distributions of their short and long USVs in the temporal domain as assessed by Ashman’s D score [86] (Fig 1c and 1f). Summary statistics for the data presented in Fig 1 are presented in S1 Table.

#### Respiratory patterns during short and long USV production

**Fig 2:**
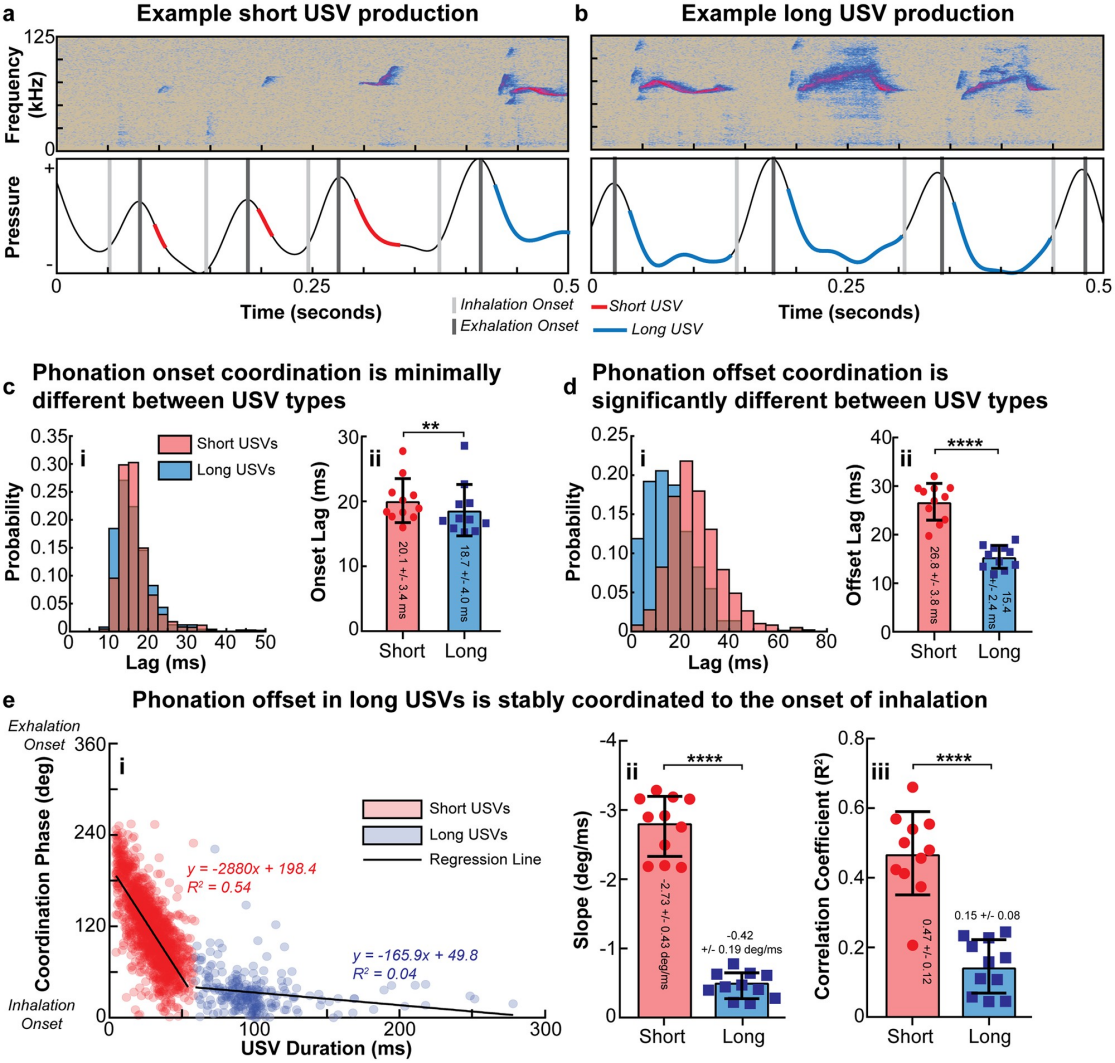
Short and long USVs arise from distinct articulatory patterns. Example spectrograms (top) and simultaneous respiratory activity (bottom) during the production of **(a)** short and **(b)** long USVs by an adult mouse. **(c.i)** Example onset lag histograms for short and long USVs in one mouse, and **(c.ii)** mean onset lags for short and long USVs across all 11 mice. **(d.i)** Example offset lag histograms for short and long USVs in one mouse, and **(d.ii)** mean offset lags for short and long USVs across all 11 mice. Additional statistic details for (c-d) are presented in S2 and S3 Tables, respectively. **(e.i)** Example scatterplot of offset coordination as a function of USV duration in 1 mouse with linear regression lines for both short and long USVs overlaid; short USVs displayed a strong correlation between offset coordination and USV duration while long USVs did not. Mean linear regression **(e.ii)** slope and **(e.iii)** correlation coefficient for short and long USVs across all 11 mice. Additional statistical details for comparisons in (e) are presented in S4 Table. In (e), linear regressions were significant for both USV types in all 11 animals; statistical details are presented in S5 Table. In (c-f), error bars indicate the mean across animals +/-1 standard deviation, and values for individual mice are represented by the 11 overlaid points.

Simultaneous recordings of respiratory activity during male mouse USV production confirmed the findings of several previous studies demonstrating that rodents, including mice, produce a single USV per sniff cycle, timed to the exhalation phase (Fig 2a and 2b) [20, 32, 83, 84, 87–90]. It is important to note that use of the terms “sniff” and “sniffing” in this study denotes the rapid inspiratory and expiratory movements occurring during active olfactory sensation and exploratory behavior, which has been thoroughly characterized in the olfactory literature [91–93]. One sniff cycle therefore refers to an inspiratory movement (colloquially known as a “sniff”) followed by an associated expiratory movement, during which a USV is produced.

We found that the onset of phonation for USV production was tightly and stably timed to the onset of exhalation for both short and long USVs (Fig 2a–2c and S2a Fig). Short USVs had a mean onset lag of 20.1 +/- 3.4 ms and long USVs had a mean onset lag of 18.7 +/- 4.0 ms; this difference was significant (p < 0.005, Wilcoxon match-pairs signed rank test) though small in magnitude. Conversely, we observed that short and long USVs differed dramatically in how the offset of phonation was coordinated to the onset of inhalation (and therefore the offset of exhalation) (Fig 2a, 2b and 2d). First, short USVs were found to have a significantly longer offset lag than long USVs (p < 0.0001, paired t-test) (Fig 2d and S2b Fig), but more importantly, the relationship between USV duration and phonation offset was found to be categorically different between the two USV types. Specifically, a significant strong linear correlation between offset coordination phase (a phase of 0 degrees indicates phonation offset was simultaneous with inhalation onset, 360 degrees indicates it was simultaneous with exhalation onset) and USV duration was observed for short USVs. However, this same strong linear relationship was not observed for long USVs, with long USVs exhibiting significantly lower correlation coefficients and regression line slopes than short USVs (both p < 0.0001, paired t-test) (Fig 2e). Therefore, short USV phonation offsets are variably timed with offset of exhalation, such that a longer short USV is simply the product of phonating for a longer portion of the exhalation phase. However, long USVs are stably coordinated with the onset of inhalation; longer long USVs are therefore the result of longer exhalations, as the portion of the exhalation phase during which phonation occurs is approximately constant in long USV production.

Summary statistics and statistical details for the analyses and statistical tests presented in Fig 2 and S2 Fig are presented in S2–S4 Tables. Statistical details for the linear regression analysis performed in Fig 2e.i are presented in S5 Table

#### Silent interval classes

**Fig 3:**
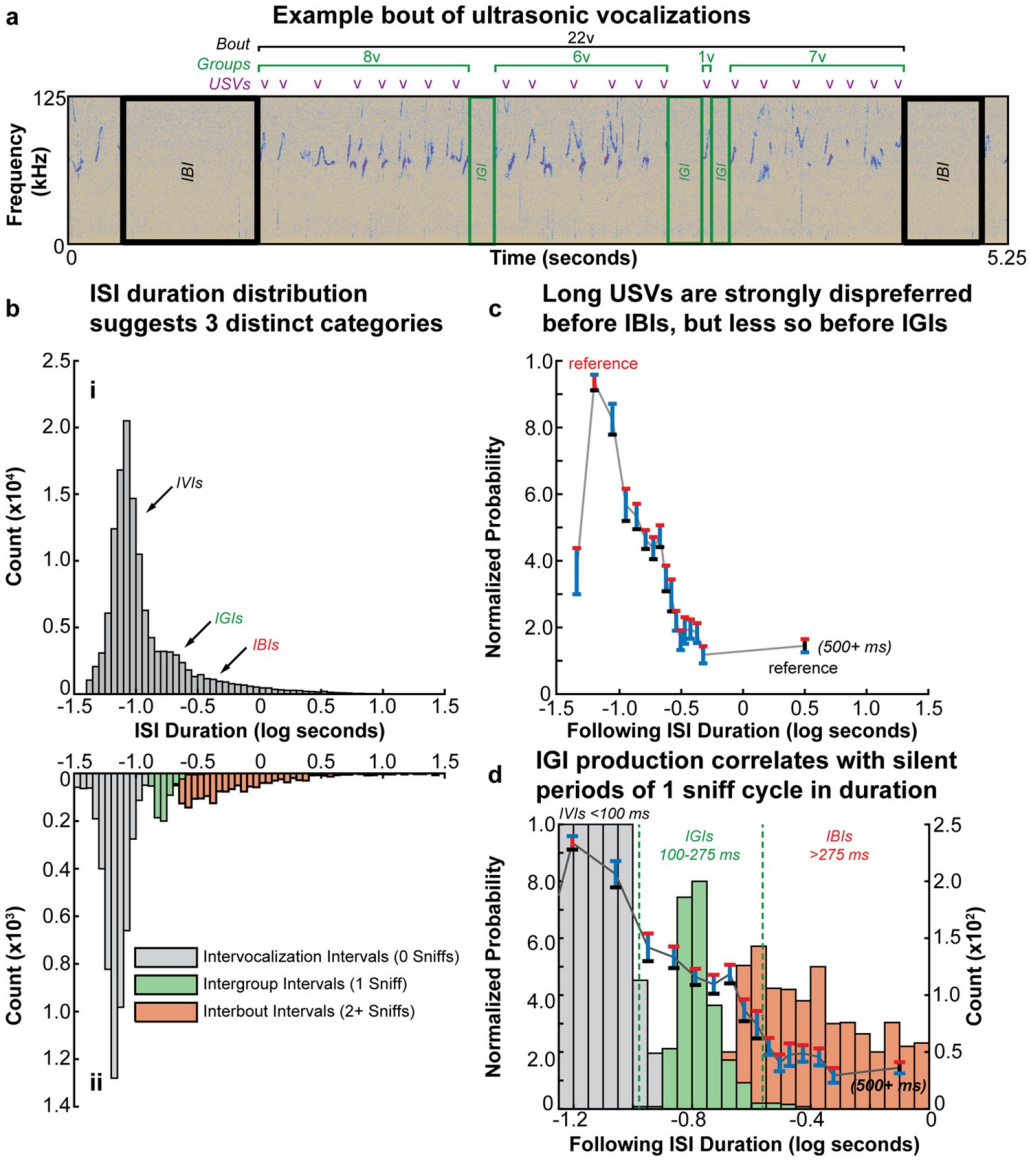
Intervening silent interval classes exhibit unique features. **(a)** Example spectrogram showing a bout of 22 USVs produced by an adult mouse. Three distinct ISI categories were observed: short duration IVIs separating individual USVs, medium duration IGIs separating groups of USVs, and long duration IBIs separating bouts of USV groups. **(b.i)** Pooled histogram of ISI durations produced by all 19 mice in all adult recording sessions. **(b.ii)** Pooled histogram of ISI durations from 11 adult mice from all freely moving plethysmography sessions. ISIs arising from silent inhalations (0 complete sniff cycles), 1 nonvocal sniff cycle, or 2+ nonvocal sniff cycles are indicated; the corresponding distributions in (b.i) are indicated with arrows. **(c)** The normalized probability of observing a long USV as a function of following ISI duration. Long USV production was most likely prior to IVIs 50-75 ms (red vertical bar) in duration and least likely prior to IBIs 500+ ms in duration (black vertical bar). However, a plateau of intermediate probability which was significantly higher than prior to IBIs (black lower error bars) and significantly lower than prior to IVIs (red upper error bars) was observed for ISIs 100-275 ms in duration, which were designated as IGIs (p < 0.05, repeated measures one-way ANOVA with Dunnett’s correction for multiple comparisons; see S6 Table for additional statistical details). **(d)** The plot in (c) superimposed over the histogram in (b.i); the plateau of intermediate long USV production probability (IGIs) corresponds to silent intervals arising from a single nonvocal sniff cycle. In (c-d), error bars indicate the mean across animals +/-1 standard error.

Individual mouse USVs are separated from one another by brief intervening silent intervals (ISIs), while USV series are separated from other series by longer ISIs (Fig 3a). Previous studies have suggested that there may be as many as three discrete classes of ISIs in male mouse courtship USVs [21, 94–96] and therefore two classes of USV series, but functional differences between the proposed classes have not been demonstrated. In agreement with these previous reports, we also observed three classes of ISIs in male USVs which constituted distinct distributions in the temporal domain (Fig 3b). We define these three ISI classes as follows: 1) short duration intervocalization intervals (IVIs), which separate individual USVs, 2) medium duration intergroup intervals (IGIs), which separate groups of USVs, and 3) long duration interbout intervals (IBIs) separating bouts of USVs (which can contain multiple groups) from other bouts (Fig 3a and 3b). Note that we specifically refer to USV series separated by IGIs as “groups” and series separated by IBIs as “bouts”, though the term “bout” is commonly used in the mouse USV literature to refer to a generic series of USV [1, 3, 10, 12, 14, 15, 21, 97].

As USV production is tightly bound to ongoing sniffing behavior [20, 87], we investigated the respiratory activity occurring during the production of silent intervals, and found that the three proposed ISI classes arise from distinct respiratory events. Specifically, short duration IVIs resulted from silent inhalations between consecutive vocal sniffs (a sniff cycle where a USV is produced on the exhalation), while medium duration IGIs arose from a silent inhalation followed by a single nonvocal sniff (a sniff cycle where no USV is produced) intervening between vocal sniffs, and long duration IBIs were the result of a silent inhalation followed by multiple consecutive nonvocal sniffs (Fig 3b.ii). Therefore, while IVIs were qualitatively different in their production than the other two proposed ISI classes, IGIs and IBIs arose simply from a continuous difference in the number of nonvocal sniffs between vocal sniffs.

The absence of a categorical difference between IGIs and IBIs called into question whether the two were functionally different from one another, and whether a bout should be defined as a series of USVs produced on consecutive sniff cycles [20] rather than a series separated by long duration ISIs (our putative IBIs), as has been common in the literature [1, 12, 15, 21, 94–96]. To further investigate this issue, we examined regularities in USV production adjacent to ISIs of continuously varying duration. We found that long USV production was more likely preceding short duration ISIs and less likely preceding long duration ISIs. However, a linear decay in long USV production probability was not observed between the point of maximal probability for ISIs of 50-75 ms and minimal probability for ISIs of 500+ ms; instead a plateau of intermediate probability was observed between for ISIs between 100-275 ms. Long USV production probability was significantly less for ISIs within this intermediate range than for short 50-75 ms ISIs and also significantly greater in probability compared to ISIs of 500+ ms (p < 0.05, repeated-measures one-way ANOVA) (Fig 3c). Furthermore, this region of intermediate probability overlapped with temporal distribution of IGI durations, which arose from a single nonvocal sniff cycle (Fig 3d). In conclusion, IGIs and IBIs, though delineated by a continuous difference in nonvocal sniffs, are distinct from one another when the properties of adjacent USVs are considered. Therefore, in the absence of respiratory data, we defined IGIs using these regularities in USV production as silent intervals of 100-275 ms in duration (as a proxy for a single nonvocal sniff cycle), IVIs as intervals of less than 100 ms in duration (as a proxy for a silent inhalation but no nonvocal sniff cycles), and IBIs as intervals of greater than 275 ms in duration (as a proxy for 2+ nonvocal sniffs cycles).

In summary, we identify three classes of ISI in male mouse courtship USVs that organize vocal units into multielement series. Consequently, bouts (separated from other bouts by IBIs) represent a hierarchical multielement series that contain a single class of subseries, groups (separated within a bout by IGIs), in which individual USVs (separated with a group by IVIs) are ordered (Fig 3a). To our knowledge, this is first evidence of hierarchical organization in mouse USVs.

It is worth noting that we also observed a significant reduction in long USV production probability preceding ISIs of 40-50 ms in comparison to ISIs of 50-75 ms (p < 0.0001, repeated measures one-way ANOVA). We hypothesize that this is due to a lengthened inhalation directly following long USVs, as the expiratory duration required for long USV production is dramatically increased compared to short USV production and nonvocal sniffing. This hypothesis is supported by the fact that a similar reduction in long USV production is not observed following ISIs of 40-50 ms (p = 0.0573, repeated measures one-way ANOVA), though a trend for reduced probability is observed (S3 Fig). This trend is most likely the result of the tendency to produce long USVs in series (Fig 5), and therefore long USVs would be less likely to follow a short duration IVIs of 40-50 ms than a longer duration IVI.

Statistical details and summary statistics for the analyses described in Fig 3 and S3 Fig are presented in S6 and S7 Tables, respectively.

### Multielement call production

#### USV series properties

**Fig 4:**
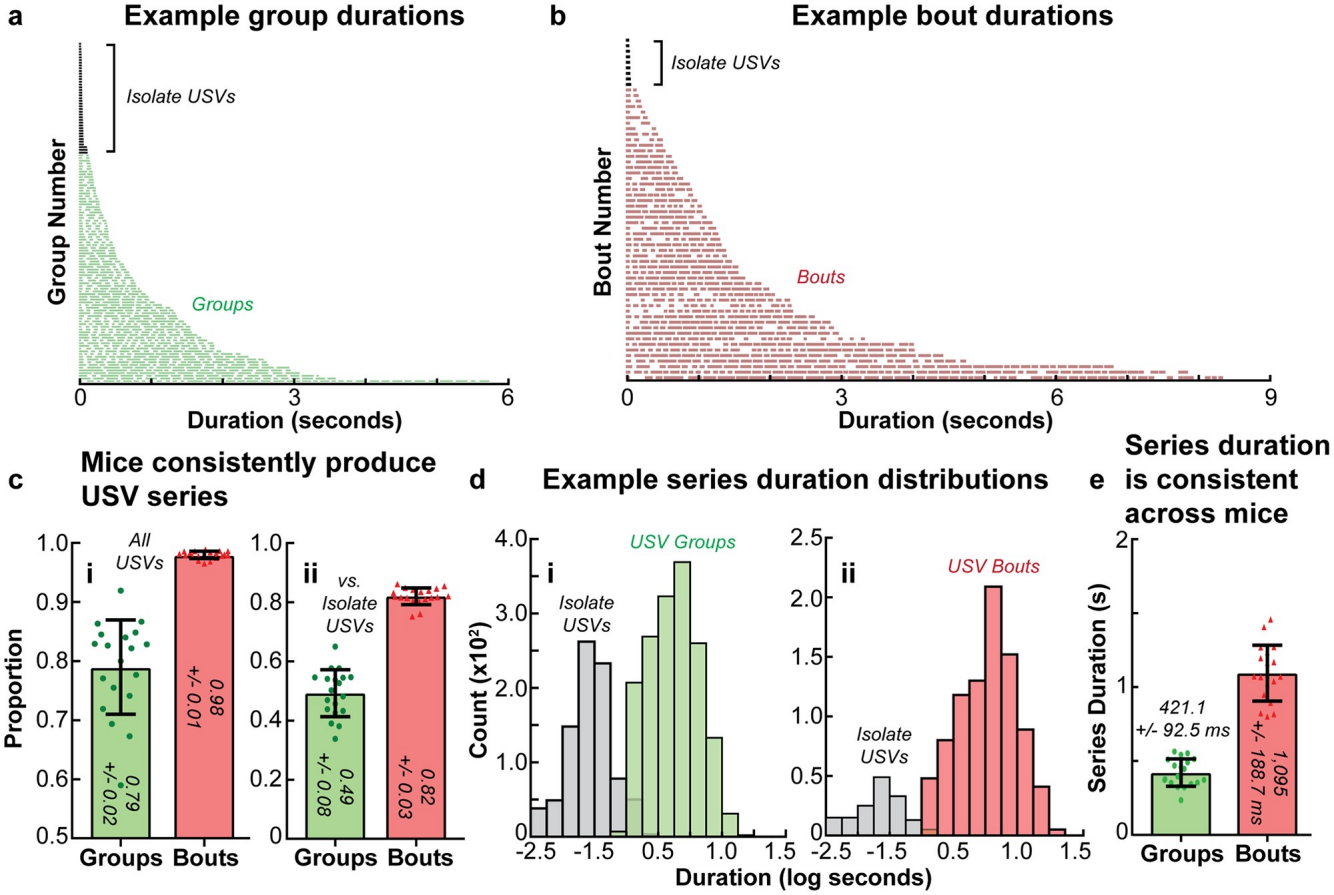
USV series production is consistent across mice. Example **(a)** group and **(b)** bout durations in a single recording session of an adult mouse, with isolate USVs indicated. Series and isolate USVs are stacked according to duration, with the shortest on top and the start of the vocal unit on the left. Each individual line represents a USV and white spaces between lines represent ISIs, with the size of the lines and white spaces representing to scale the duration of the USVs and ISIs, respectively. The mean proportion of **(ci)** of USVs produced in groups and bouts, and **(cii)** groups and bouts as opposed to isolate USVs. Additional statistical details are presented in S8 Table. Histograms of isolate USV and **(di)** group and **(dii)** bout duration from all recording sessions of one adult mouse; isolate USVs and USV series form distinct distributions. **(e)** Mean duration of groups and bouts across all 19 mice. Additional statistical details are presented in S9 Table. In (e), mean durations are transformed from log seconds to represent series duration with a more familiar measure. In (c) and (e), error bars indicate the mean across animals +/-1 standard deviation, and values for individual mice are represented by the 19 overlaid points.

Having empirically defined two classes of hierarchically ordered multielement USVs series—USV groups, which are ordered into longer bouts (Fig 3a)—we next examined the production of these series in the courtship USVs of adult male mice. Mice were found to produce the vast majority of the USVs in series (Fig 4a and 4b), with exceedingly few USVs produced in isolation (“isolate USVs”). Specifically, 79 +/- 2% and 98 +/- 1% of USVs were produced in groups or bouts, respectively (Fig 4c.i). When considering all vocal units (both USV series and isolate USVs) delineated by at least the minimum IGI duration (as the first and last groups in a bout are preceded and followed by an IBI, as illustrated in Fig 3a), 49 +/- 8% of these vocal units were multi-USV groups, which is consistent with a previous study that defined a series as USVs produced on successive vocal sniffs [20]. Likewise, 82 +/- 3% of vocal units separated by IBIs were multi-USV bouts (Fig 4c.ii). This 4:1 ratio of bout to isolate production was extremely consistent across mice, with a coefficient of variation (CV) of only 3.47% across mice (S8 Table), and was in agreement with a previous report of bout production during courtship behavior [15]. Note that we consider all vocal units separated by IGIs within a bout, both USV groups and isolate USVs, as the constitutive groups of that bout. Therefore, a true isolate USV would be one that is flanked by IBIs, as isolate USVs adjacent to IGIs are still produced in a series (*i.e*. in a bout).

Both classes of series displayed an approximately exponential duration distribution (Fig 4a and 4b). On a logarithmically spaced time scale, isolate USV and USV series formed well-separated distributions in the temporal domain (Fig 4d). Groups had an average duration of 421.1 +/- 92.5 ms and bouts had an average duration of 1,095 +/- 188.7 ms (Fig 4e) (values transformed from log seconds for descriptive purposes). The median number of USVs per group was 3 and per bout was 5, while the median number of groups per bout was 2 (S4 Fig). Summary statistics for the data presented in Fig 4 and S4 Fig are available in S8–S10 Tables.

#### Series rhythmic structure

**Fig 5:**
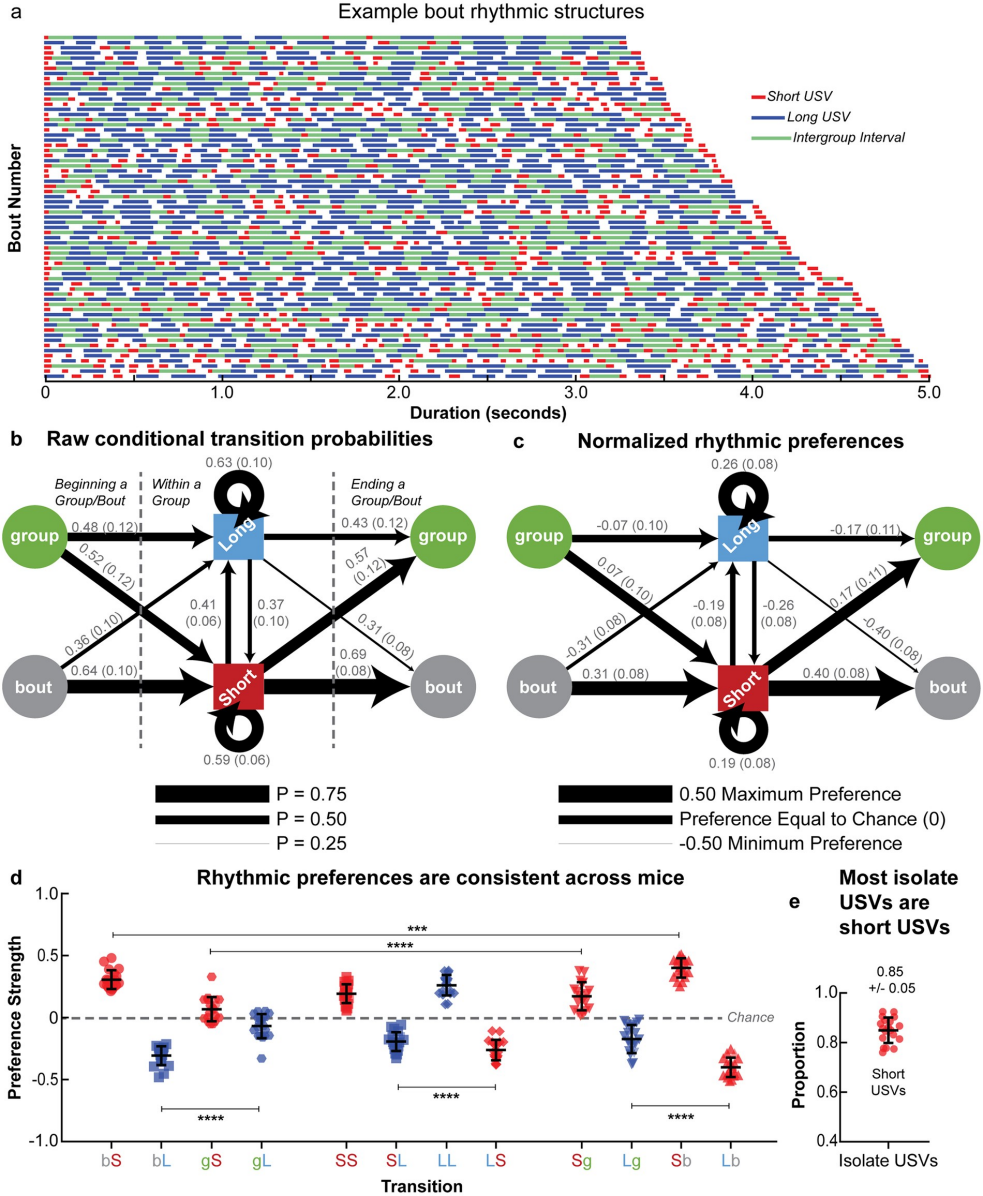
Series rhythmic structure is consistent across mice. **(a)** Example bouts produced by an adult mouse. Bouts are stacked according to duration, with the shortest on top and the start of the bouts on the left. Red lines indicate short USVs, blue lines indicate long USVs, green lines indicate IGIs, and white spaces indicate IVIs, with the length of the lines and spaces representing to scale the duration of the USVs and ISIs. Mean **(b)** raw conditional probabilities and **(c)** normalized Preference Scores for each transition across all 19 mice. The numbers adjacent to the bars indicating the transition represent the mean across mice with the standard deviation in parentheses. Additional statistical details are presented in S11 and S12 Tables for (b) and (c), respectively. **(d)** Average Preference Scores for each transition in all 19 adult mice, depicting the values for individual mice. **(e)** Proportion of isolate USVs observed to be short USVs. In (d) and (e), error bars indicate the mean across animals +/-1 standard deviation, and values for individual mice are represented by the 19 overlaid points. In (b-c), IGIs are represented by the green circles while IBIs are indicated by the grey circles. In (d), “b”, “g”, “S”, and “L” correspond to IBIs, IGIs, short USVs, and long USVs, respectively.

We next investigated how USVs were ordered within series to assess the gross rhythmic structure of groups and bouts. Both types of series were found to exhibit a general rhythmic pattern where consecutive short USVs were produced at series onset and offset, and long USVs were produced in succession series-internally, however consecutive short USV production was observed at bout-internal group onset and offset with reduced regularity compared to bouts (Fig 5a and 5b). Furthermore, the observed conditional transition probabilities were similar across mice, demonstrating that this rhythmic pattern is generalized feature of B6 adult male courtship USVs (S5 Fig).

From raw conditional transition probabilities (Fig 5b), we then calculated Preference Scores, which allow for a normalization of conditional transition probabilities across mice [21], as individual mice varied in their production rates of both USVs and ISI classes. Such individual differences in production could therefore skew the observed transition probabilities; for example, a mouse that produces mostly short USVs would have a higher probability of producing a short to short transition than a mouse that produces mostly long USVs, although both animals exhibit a tendency to produce short USVs successively. Furthermore, Preference Scores allow for a comparison between the observed conditional transition probabilities and what would be expected if the mice were producing transitions as inviolable “rules”, as a rule that is never violated would have a Score approaching 1 and a transition that is not permitted would have a Score approaching -1. It is worth noting that use of the words “preference” and “dispreference” is not meant to suggest a conscious preference or avoidance on behalf of the mouse, but rather reflects the degree to which a given transition is observed in relation to an alternative transition, which is similar to how this terminology is used in the linguistics literature.

After converting the raw conditional transition probabilities to Preference Scores, the same general rhythmic structure of USV series was apparent (Fig 5c), in agreement with our analysis in Fig 5b and the findings of Castellucci *et al*. (2016) [21]. This structure was found to be highly consistent across mice, as the same transitions were preferred in the USV series of all mice (except for the preference to start groups with short USVs, which was essentially at chance levels); for example, short USVs were preferred at the beginning of bouts for all mice, and long USVs were always dispreferred in this position (Fig 5d). In addition, we also observed that the preference to end groups and bouts with short USVs was significantly stronger than to begin groups and bouts with short USVs (p < 0.0001 and p = 0.0002, respectively, repeated measures one-way ANOVA). The production of long USVs was likewise significantly more dispreferred bout-initially and bout-finally compared to group-initially and group-finally (both p < 0.0001, repeated measures one-way ANOVA), and transitioning from a short to a long USV was significantly more preferred than transitioning from a long USV to a short USV (p < 0.0001, repeated measures one-way ANOVA).

Finally, most isolate USVs were found to be short USVs, as short USVs constituted 85 +/- 5% of all observed isolates across mice (Fig 5e, S11 Table). Additional summary statistics for the analyses illustrated in Fig 5 and S5 Fig are presented in S11 and S12 Tables, and statistical details for the ANOVA performed in Fig 5d are presented in S13 Table.

#### Fine-scale series temporal structure

**Fig 6:**
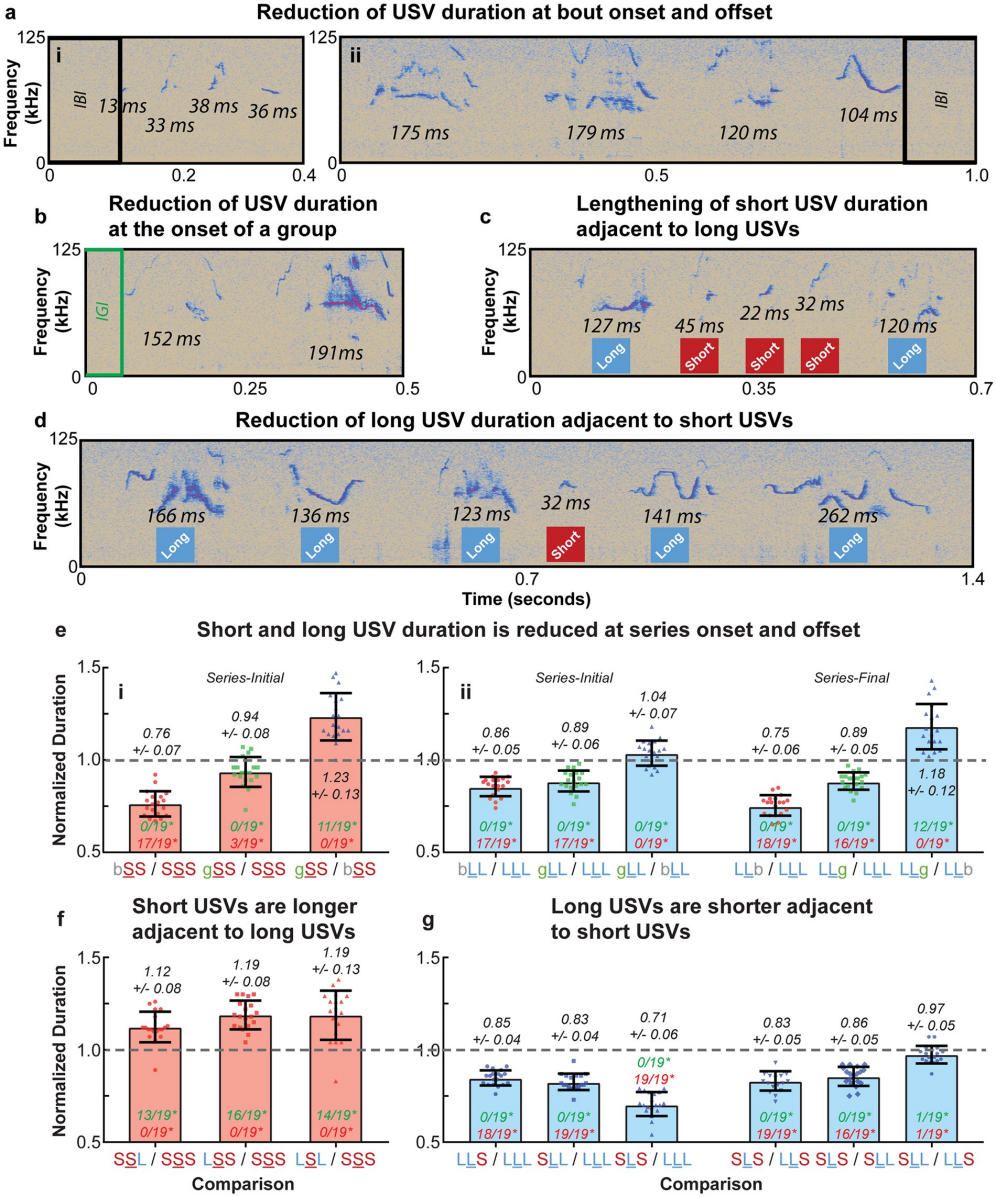
USV series exhibit consistent fine-scale temporal structure. **(a)** Example spectrogram from an adult male mouse showing reduction of USV duration (a.i) bout-initially (a.ii) bout-finally. **(b)** Example spectrogram from an adult mouse depicting reduction of long USV duration group-initially. Example spectrograms of USVs produced by an adult mouse demonstrating **(c)** the reduction of long USV duration adjacent to a short USV, and **(d)** the lengthening of short USV duration adjacent to long USVs. **(e.i)** Average normalized durations of short USVs at series onset across all 19 mice. Short USVs were significantly shorter at bout onset in a majority of mice, but not group onset (see S14 and S15 Tables for statistical details). **(e.ii)** Average normalized durations of long USVs series-initially (left) and series-finally (right). In most mice, long USVs were significantly shorter at the onset and offset of both groups and bouts, and were significantly shorter at bout offset compared to group offset (see S14 and S16 Tables for statistical details). **(f)** Average normalized durations of short USVs adjacent to long USVs across all 19 adult mice. In a majority of mice, short USVs were significantly longer when adjacent to long USVs. See S18 and S19 Tables for statistical details. **(g)** Average normalized durations of long USVs adjacent to short USVs across all adult 19 mice. In all environments examined, long USVs were significantly shorter when adjacent to short USVs in nearly all mice. See S18 and S20 Tables for statistical details. In (e-g), “b”, “g”, “S”, and “L” correspond to IBIs, IGIs, short USVs, and long USVs, respectively. Values on the plots are represented as normalized durations between the two trigrams listed on the x-axis. For example “bSS/SSS” indicates the mean duration of bout-initial short USVs (S) preceding a short USV normalized to the mean duration of short USV (S) flanked by short USVs. Error bars indicate the mean across animals +/-1 standard deviation, and values for individual mice are represented by the 19 overlaid points. The number of mice showing a significant reduction or lengthening of USV duration for each comparison is written in red and green, respectively. Summary statistics for (e) and (f-g) are presented in S17 and S21 Tables, respectively.

Having determined that USV series exhibit a stereotyped rhythmic pattern of short and long USVs, we next examined the fine-scale temporal organization of USV series to determine whether the timing of individual USVs display similar regularities. We first investigated how USVs were produced at series onset and offset, and found that USV duration was dramatically reduced at the onset and offset of both groups and bouts (Fig 6a and 6b). Specifically, a significant reduction in short USV duration at bout onset was observed in 17/19 mice (p < 0.05, one-way Kruskal-Wallis test); however, no consistent reduction in duration was observed at group onset (Fig 6e.i) or at the offset of either series type (S6a Fig). Long USVs also displayed a consistent reduction in their duration at series onset and offset, with 17/19 and 18/19 mice exhibiting significantly reduced long USV duration at bout onset and offset, respectively (p < 0.05, one-way Kruskal-Wallis test). Similarly, 17/19 and 16/19 mice demonstrated a significant reduction in long USV duration at group onset and offset, respectively (p < 0.05, one-way Kruskal-Wallis test). In addition, long USV duration was significantly more reduced at bout offset in comparison to group offset in 12/19 mice (p < 0.05, one-way Kruskal-Wallis test), however no such effect was observed series-initially (Fig 6e.ii).

The fine-scale timing of individual USVs was also found to be affected by the temporal features of adjacent USVs (Fig 6c and 6d). Specifically, short USVs adjacent to long USVs were found to be significantly longer than short USVs adjacent to other short USVs in the majority of the 19 subject mice (p < 0.05, one-way Kruskal-Wallis test); no mouse displayed a significant reduction in duration for short USVs adjacent to long USVs (Fig 6f). This increase in duration was not observed to be additive as short USVs adjacent to 2 long USVs were not consistently longer than short USVs adjacent to a single long USV, and no consistent difference in duration between short USVs following a long USV and short USVs preceding a long USV was observed (S6b Fig). For long USVs, we observed a consistent reduction in duration when adjacent to short USVs, with nearly every mouse exhibiting a significant decrease in long USV duration when adjacent to short USVs in any configuration (p < 0.05, one-way Kruskal-Wallis test); no mice displayed an increase in long USV duration in this context. The reduction in duration for long USVs was found to be additive, as the duration of long USVs adjacent to 2 short USVs was significantly shorter than the duration of long USVs adjacent to a single short USVs in nearly all mice (p < 0.05, one-way Kruskal-Wallis test). However, like short USVs, long USVs preceding a short USV were not consistently different in duration from long USVs following a short USV (Fig 6g).

Finally, we observed that temporal regularities observed at series onset and offset were combined with those occurring at transitions between USV classes when such transitions occurred series-initially or series-finally. Specifically, we observed that the short USVs of 5/19 and 17/19 mice were significantly longer at the bout onset and offset, respectively, when adjacent to long USVs in comparison to those adjacent to a short USV (p < 0.05, one-way Kruskal-Wallis test). Similarly, the bout-initial and bout-final long USVs of 12/19 and 9/19 mice, respectively, were significantly shorter when adjacent to a short USV than when adjacent to another long USV (p < 0.05, one-way Kruskal-Wallis test) (S6c Fig).

Descriptive statistics for the data described in Fig 6e and S6a Fig are presented in S14 Table, and additional summary statistics are available in S17 Table. Statistical details for the Kruskal-Wallis tests performed for the analyses in Fig 6e and S6a Fig are presented in S15 and S16 Tables. Descriptive statistics for the data presented in Fig 6f and 6g and S6b Fig are available in S18 Table, and additional summary statistics are available in S21 Table. Statistical details for the Kruskal-Wallis tests performed for the analyses in Fig 6f and 6g and S6b Fig are presented in S19 and S20 Tables. Descriptive statistics and supplementary summary statistics for the data described in S6c Fig are presented in S22 and S24 Tables, respectively. Finally, statistical details for the Kruskal-Wallis tests performed for the analysis in S6c Fig are available in S23 Table.

### Sex and age-related temporal patterns

#### Development of USV spectrotemporal features in male mice

We next investigated the development of USV temporal organization by tracking the vocal development of individual male mice from birth into adulthood. Pup isolation calls were recorded from 11 mice starting at P0 or P1 until P16, and male courtship vocalizations were recorded from P17 or P18 until P85. In eight additional mice, male courtship vocalizations were recorded from P25 until P95. For statistical analysis, the data were then split into six pup age groups: P0-P3, P4-P5, P6-P7, P8-P9, P10-11, and P12-16, and two juvenile age groups: P17-P34, and P35-P49, and finally two adult age groups: P50-P65 and P66-P95. The cutoff of P35 between juvenile groups was selected because male mice generally develop mature spermatozoa by this age [98], and rapid puberty-related weight gain is largely complete by P35 [99]. Likewise, USV production rates stabilize by P50 [21] (S1 Fig), therefore this age served to delineate juvenile from adult age groups. P65 was selected to divide the adult age groups simply to provide another a third approximately 15 day interval to compare adult and juvenile vocalizations. We did not further divide the oldest adult age group as some mice completed recording sessions at P85 and others P95 (see Methods for additional information).

**Fig 7:**
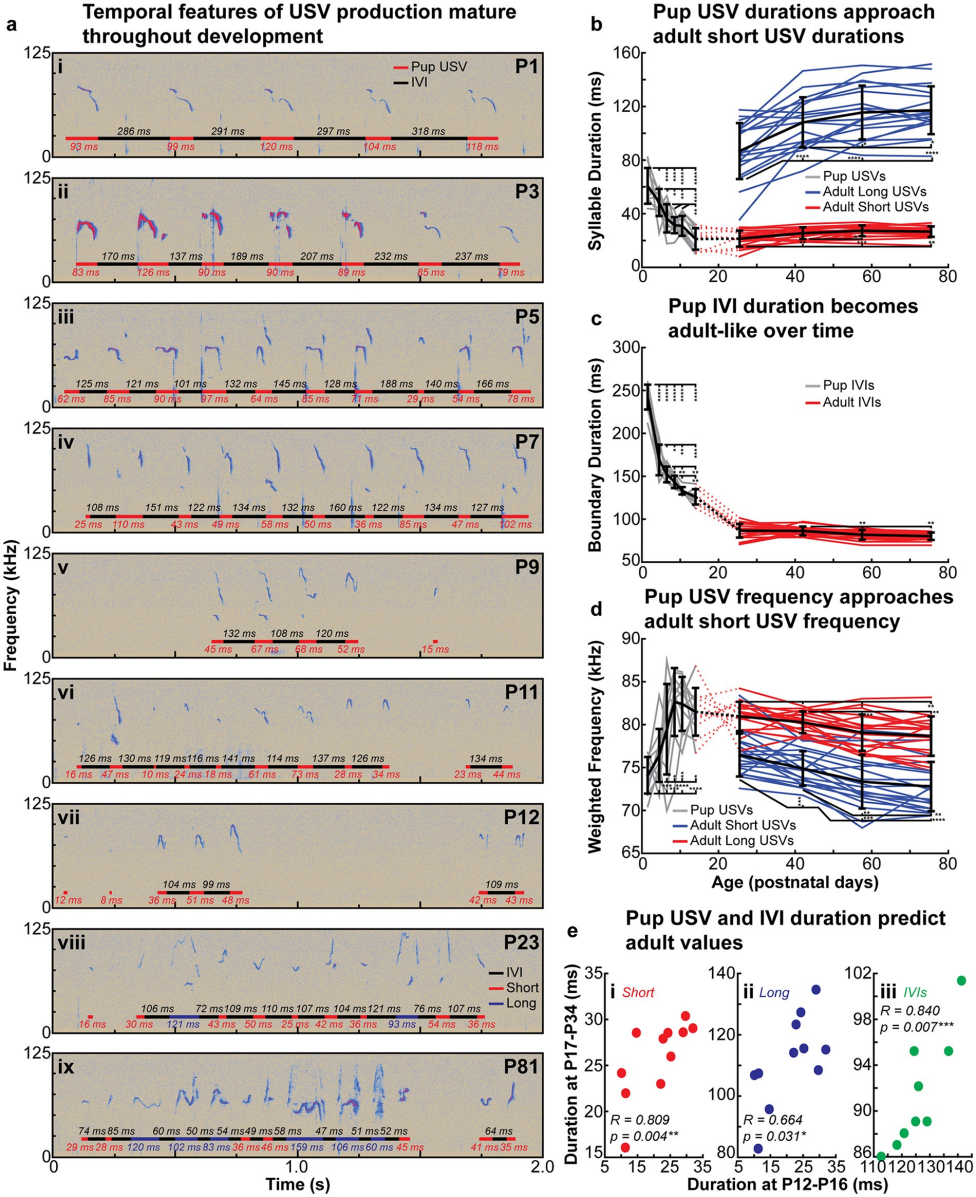
**(a)** Example spectrograms of USVs produced by a single mouse throughout development. **(b)** Mean pup USV, short USV, and long USV duration across mice over time. **(c)** Mean IVI duration across mice throughout development. **(d)** Mean weighted frequency of pup USVs, short USVs, and long USVs over time. **(e)** Mean pup USV duration at P12-P16 was significantly correlated with mean (e.i) short USV and (e.ii) long USV duration at P17-P34; (e.iii) median IVI duration at P12-P16 was also significantly correlated with median IVI at P17-P34. In (b-d), significance is assessed with repeated-measures one-way ANOVAs with Tukey’s correction for multiple comparisons; see S27 and S28 Tables for statistical details. Summary statistics are presented in S25 and S26 Tables. Error bars indicate the mean value across animals +/- 1 standard deviation. Individual lines indicate the values for each mouse. In (e), Spearman’s R and p-values for the correlation are labelled on the respective plots.

We found that the general spectrotemporal structure of adult USVs continuously developed from birth, and that several features of pup isolation USVs approached those of juvenile courtship USVs (Fig 7a). Specifically, the average pup isolation USV duration was found to significantly decrease with age (p < 0.0005, repeated measures one-way ANOVA) until it approximated the average duration of the short USVs produced by the youngest age group of juvenile males. As the juvenile mice developed into adults, both mean short USV and long USV duration (as measured using the fitting analysis described in Methods and Fig 1) was found to significantly increase with age (p < 0.01, repeated measures one-way ANOVA), but not after P50 (Fig 7b). Likewise, Ashman’s D scores for USV duration distributions of the courtship USVs also increased with age until P50 (p < 0.05, repeated measures one-way ANOVA) (S7a Fig), and the production of long USVs was found to significantly increase with age until P50 (p < 0.005, repeated measures one-way ANOVA) (S7b Fig). The only metric found to change significantly with age in adulthood beyond P50 was long USV duration variance, which significantly increased between P50-P65 and P66-P95 (p < 0.001, repeated measures one-way ANOVA); short USV variance did not change significantly with age (S7c Fig). We also observed that the median duration of IVIs (see Methods and S8 and S9 Figs for minimum and maximum IVI duration determination) significantly decreased in pup isolation USVs with age (p < 0.05, repeated measures one-way ANOVA) and approached adult-like values, but was relatively stable in the juvenile and adult USVs over time (Fig 7c).

Interestingly, the duration of pup USVs produced by the oldest pup age group was found to be significantly correlated with both short and long USV duration in the youngest juvenile age group (Spearman R = 0.809, p = 0.004 and Spearman R = 0.664, p = 0.031, respectively). Pup IVI duration was similarly significantly correlated with juvenile IVI duration (Spearman R = 0.840, p = 0.007) (Fig 7e). Therefore, the major temporal features of a mouse’s courtship USVs are related to the features of its pup isolation USVs, suggesting that the two call types are not independent of one another, and may rely on shared biological mechanisms.

We lastly examined how the gross spectral structure of the male USVs developed with age by analyzing weighted frequency (the weighted average frequency as calculated from the power spectrum of a USV) of pup and adult calls. We found that the weighted frequency of pup USVs significantly increased with age (p < 0.05, repeated measures one-way ANOVA) until the average short USV weighted frequency in the youngest adult age group was approximated. Short and long USV weighted frequency then significantly decreased with age (p < 0.05, repeated measures one-way ANOVA), but not after P50 (Fig 7d). Unlike pup USV duration and IVI duration, pup USV weighted frequency was not correlated with the weighted frequency of juvenile short or long USVs (Spearman R = -0.445, p = 0.173 and Spearman R = -0.245, p = 0.468, respectively) (S7d Fig).

Summary statistics for pup and adult developmental data is presented in S25 and S26 Tables, respectively. Statistical details for the ANOVAs performed for the analyses in Fig 7 and S7 Fig are presented in S27 and S28 Tables for pups and adults, also respectively.

#### Discrimination of USV type by temporal features

**Fig 8:**
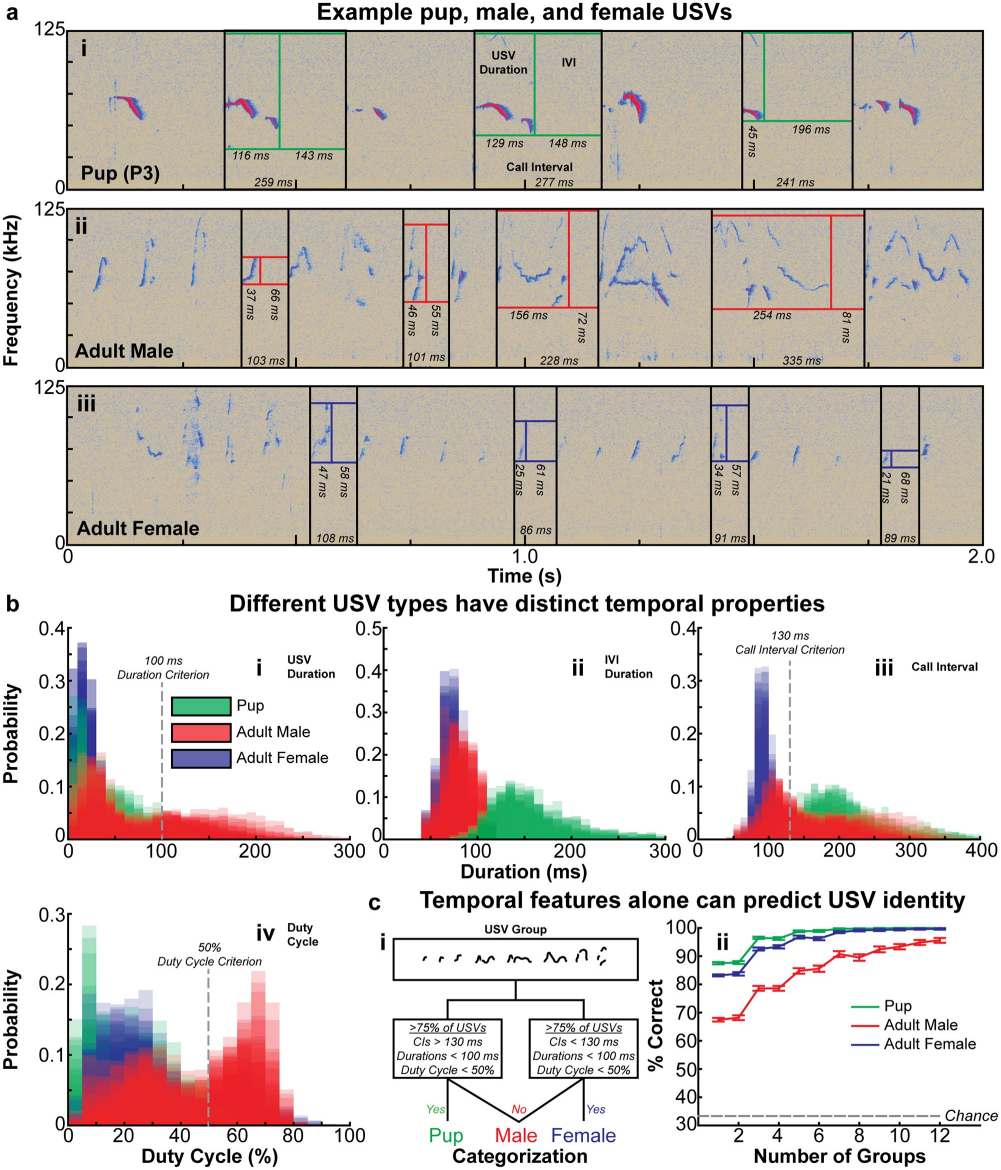
Call temporal features discriminate different USV types. **(a)** Example spectrograms of USVs produced by (a.i) a mouse pup, (a.ii) an adult female mouse, and (a.iii) an adult male mouse, with USV duration, IVI duration, and call interval values labelled. **(b)** Histograms depicting the distribution of (b.i) USV duration, (b.ii) IVI duration, (b.iii) call interval, and (b.iv) duty cycle values from 11 pups (P0-P16), 19 adult males (P50-P95), and 15 adult female mice (all >4 months). For each USV type, histograms from individuals are transparent and plotted on the same axes to illustrate the variability within and across mice. **(c.i)** Overview of the classifier used to categorize groups of USVs produced by pups, adult females, or adult males. **(c.ii)** Performance of the classifier on a subset of 250 randomly selected groups of USVs produced by pups, adult females, or adult males over 100 iterations as a function of the number of groups used for classification. Error bars indicate the mean across iterations and the 95% confidence interval (see S29 Table for summary statistics and statistical details).

Finally, since pup and adult male USVs displayed systematic differences in their temporal properties (though to varying degrees depending on the age of the animals), we wanted to determine whether the temporal structure of mouse multielement calls alone could be informative in determining the identity of the caller. For this analysis, we included USVs produced by adult female mice in addition to adult male courtship USVs and pup isolation calls. We observed that the gross temporal structure varied considerably between the three call types (Fig 8a). Specifically, we found that, while adult males produced both short and long duration USVs, pups and female mice nearly exclusively produced short USVs less than 100 ms in duration (Fig 8b.i). Furthermore, IVI duration (see Methods, S8, S9 and S10c Figs for minimum and maximum IVI determination) was also dissimilar between pups, who mostly produced IVIs of 100 ms and longer, and adult males and females who produced short duration IVIs less than 100 ms (Fig 8b.ii). Likewise, the three call types were also well delineated in terms of call interval (time between successive USV onsets in a group [USV duration added to following IVI duration]) and duty cycle (percentage of the call interval occupied by the USV) values (Fig 8b.iii and 8b.iv).

Based solely on the distributions of USV duration, call interval, and duty cycle values for each type of USV, we then designed a linear classifier to evaluate the effectiveness of these temporal measures in discriminating between the different USV types (we did not use IVI duration as a criterion since the different USV types had differing maximum IVI durations). The classifier was set to categorize a USV group (a series delineated by an ISI greater than the maximum IVI) as produced by a pup if 75% of the call intervals in the group were >130 ms, 75% of the USV durations were <100 ms, and 75% of the duty cycle values were <50%, and as produced by a female if the same criteria were met but 75% of the call intervals were <130 ms. All other groups were classified as adult male USVs (Fig 8c.i).

When presented 100 iterations of 250 random groups of each USV type, the classifier correctly identified 87.5 +/- 1.9% of pup USVs, 83.2 +/- 1.8% of adult female USVs, and 67.5 +/- 3.3% of adult male USVs. These values were significantly higher than the chance value of 33.3% as assessed by calculating 95% confidence intervals (Fig 8c.ii, S29 Table). However, as USV groups are generally not produced in isolation, we tested the classifier’s performance when it considered 2-12 groups and made a decision based on which categorization was most common in the set of groups it considered (see Methods for more details). The classifier’s performance increased to over 90% correct for pup and adult female USVs after considering 3 groups, and for adult males after considering 7 groups (Fig 8c.ii). The success of this simple classifier demonstrates the potential utility of USV temporal features in determining the identity of a vocalization’s producer.

## Discussion

In summary, we found that male B6 mice produced two classes of USVs that differed in their temporal features (Fig 1) and articulatory properties (Fig 2). We observed that nearly all USVs were produced in series (bouts) (Fig 4), which were comprised of subseries (groups) consisting of USVs produced on consecutive exhalations (Fig 3). USV series were found to exhibit a characteristic rhythmic structure (Figs 5 and 6) that was sufficient for mathematical discrimination between male courtship USVs, pup isolation USVs, and USVs produced by adult female mice (Fig 8). Finally, we observed that the spectrotemporal features of male courtship USVs developed continuously from birth into adulthood, and found that the temporal properties of a given pup’s USVs were predictive of the same features in its adult USVs (Fig 7).

### USV production

Male B6 mice were found to produce two classes of USVs with distinct durations: short USVs and long USVs, replicating the findings of Castellucci *et al*. (2016). In addition to their unique temporal features, these two USV classes were produced with distinct articulatory strategies (*i.e*. patterns of coordination between respiration and phonation), suggesting that they arise from separate motor commands. Specifically, whereas phonation onset is consistently timed to exhalation onset for both USV classes, phonation offset for short USVs is variably timed to exhalation offset while the phonation offset for long USVs is stably coordinated to exhalation offset. In addition to the differences in vocal-respiratory coordination, long USVs also require a dramatic slowing of the sniff cycle for their production. For example, consistent with previous reports, we observed sniff rates between 8-12 Hz during olfactory exploration [93] and short USV production [20], but this rate could decrease to lower than 3 Hz for a 250+ ms long USV.

The neural mechanisms underlying long USV production and the requisite respiratory control is unknown, as is the function of the two USV types. Additionally, it is unclear how common the production of short and long USVs is across mouse strains and rodents in general. For example, the data presented in previous studies imply that adult male CBA/CaJ mice either produce only short duration USVs or their short and long USVs are not as temporally distinct as B6 mice [1, 100]. However, a study examining the 22 kHz alarm USVs of rats (*Rattus norvegicus*) reported a bimodal USV duration distribution comparable to what we observed for B6 mice [101], suggesting that other rodent species may also produce of temporally distinct USV classes via similar mechanisms.

The identification of distinct, stable articulation strategies for short and long USVs represents a significant advancement in the study of mouse USVs, as it is evidence that the production of these two USV types is a controlled aspect of the mouse vocalization system in B6 mice. Unlike rat USVs, whose articulatory properties have been thoroughly examined [20, 83, 87–90, 102] and whose various subtypes are known to arise from distinct, consistent laryngeal and respiratory articulatory patterns [83, 90], studies of articulation during mouse USV production have largely been restricted to examinations of respiratory activity during vocalization [20] as well as investigations of the precise USV phonation mechanism [103–107]. Consequently, the spectrotemporal features of mouse USVs which arise from distinct articulatory patterns are unknown, although this information is crucial for interpreting the effects of experimental manipulations on USV acoustic structure.

For example, numerous mouse models of human communication disorders as well as conditions affecting speech production have been reported to produce abnormal USVs [21, 85, 95, 96, 108–117]. However, it is unclear whether the USV production system itself has been affected in these animals, or instead an unrelated alteration is responsible for the observed vocalization deficits. For instance, homozygous *Foxp2* knockout pups display stark vocal deficits [118], however these animals also exhibit severe, eventually fatal, lung deformities that affect respiration [119] and consequently USV production. Likewise, mice with mutations resulting in mild laryngeal structural abnormalities produce pup isolation USVs with fine spectral defects, while gross spectrotemporal features such as mean frequency and call duration are unaffected [120].

Similarly, small variations in laryngeal configuration and respiratory activity have also been found to result in nonlinear, abrupt changes in the spectrotemporal structure of USVs [103, 105]. Therefore, the motor behavior of a mouse during USV production may affect USV acoustic structure if it passively alters vocal tract configuration or respiratory dynamics although the vocalization system itself is unchanged, as has been suggested previously [121]. Such an effect has been observed in rats, whose frequency modulated 22 kHz calls were found to be produced nearly exclusively while the animal is visibly moving the tongue or mouth, for example during grooming, suggesting that these calls are at least partially a byproduct of non-vocal oromotor activity [122]. Similarly, Mongolian gerbils produce most of their USVs during hopping or locomotion, and the magnitude of the movement executed during USV production is correlated with the amplitude of the call itself [123].

Respiratory movements are specifically known to be tightly coupled to locomotor movements in mammals [124], and USV production has likewise been shown to be loosely coupled to stride kinematics in rats [87]. Consequently, simultaneous locomotor activity may result in additional acoustic structure and variability in USV production as the thorax and airways are compressed and deformed during movement. This hypothesis is supported by the observation that USV call rate in rats in positively correlated with running speed, with more acoustically complex USVs being produced at faster running speeds [88]. In addition, USV production has been shown to be tightly coordinated to both whisking [91, 125] and head movements during sniffing [87] in rats, suggesting the active olfactory and tactile exploration that a rodent engages in during USV production, in addition to general locomotion, may also affect USV acoustic structure. This notion is consistent with the finding that that male mice produce more variable and acoustically complex courtship vocalizations while actively interacting with a female in comparison to sniffing female urine alone [94] and while attempting to mount a female in comparison to sniffing a female or female odorants [126]. Interestingly, male mice have also been shown to produce USVs with more complex spectrotemporal structure in response to female odorants when also paired with another awake male, but not when presented with male olfactory cues or playback of male USVs, and this effect was found to be weaker when the vocalizing male was paired with an anesthetized male [127]. However, this study also reported that the male mice spent most of their time vocalizing while physically interacting with the awake male partner, consistent with the hypothesis that differences in exploratory behavior and locomotor activity could have resulted in at least some of the observed differences in USV production.

Furthermore, it is possible that global arousal levels in the examples discussed above also affected USV acoustics. For example, the USVs of prairie vole pups become higher in frequency, shorter, and less frequency modulated as heart rate increases [128]; similar arousal-dependent effects on vocalization acoustics have been observed in marmosets (*Callithrix jacchus*) [129] and human infants (*Homo sapiens*) [130]. Consequently, as male mice produce the majority of their courtship USVs during active pursuit of and physical contact with female mice and male competitors [13, 14, 127, 131–133], any experimental manipulation affecting not only the ability of a mouse to engage in this behavior but also its arousal levels during courtship may result in unintended acoustic consequences in its USVs.

In conclusion, additional studies are required to investigate the production mechanism of mouse USVs in order to identify which acoustic features are under active control of the vocalization system, and which features are instead readouts of ongoing nonvocal motor behavior or global arousal. While the subtle spectrotemporal properties of short and long USVs vary across animals (as they likely do in different behavioral contexts), the production of the two USV classes and their underlying articulatory patterns is consistent, as these features appear to be an intrinsic and controlled aspect of male courtship USVs in at least B6 mice. Therefore, manipulations that alter the overall production of the two USV classes, as is observed in *Foxp2* heterozygous knockout mice which do not produce long USVs [21], would be expected to have affected the vocalization production system itself.

### Series production

The vast majority of USVs were found to be produced in series, with short duration silent IVIs (less than 100 ms) delineating one USV from another. IVIs arose from silent inhalations between the exhalations of sequential vocal sniffs (one USV is produced per exhalation phase of the sniff cycle), in agreement with previous findings [20]. Groups of USVs produced on consecutive vocal sniffs were separated from other groups by medium duration IGIs (between 100 and 275 ms), which were found to result from a single nonvocal sniff cycle. One or more USV groups comprised bouts, which were delineated by long duration IBIs (more than 275 ms) consisting of multiple nonvocal sniff cycles. In addition to their distinct temporal and respiratory properties, the three ISI types also differed in the properties of USVs adjacent to them. Specifically, long USV production was most preferred preceding IVIs and least preferred prior to IBIs, with long USV production preference at an intermediate level prior to IGIs. In addition, USVs were significantly shorter when preceding IBIs in comparison to when preceding IGIs. These results provide evidence for different classes of multielement series in mouse USVs as well as justification for defining mouse USV series either as USVs produced on sequential vocal sniffs [20] or as series separated by long duration silent intervals comparable to the IBIs described in this study [21, 95, 96]. However, we demonstrate that these two alternative definitions are actually distinct types of vocal series that are hierarchically organized.

After empirically defining two classes of USV series, we were then able to evaluate their production by male mice. We found that all mice produced both groups and bouts at consistent rates compared to isolate USVs, with approximately 50% and 80% of vocal units separated by the minimum IGIs and IBI durations, respectively, being multielement series. These values are in agreement with previous reports that defined their USV series such that they approximated the groups and bouts defined in this study [15, 20]. Furthermore, the proportion of USV bouts versus isolate USVs produced by mice in courtship contexts is comparable to what is observed for harp seals, who produce over 90% of their vocalizations as multielement calls during breeding season [46]. This suggests the production of multielement calls in mice and seals may indeed be a response to similar ecological factors, specifically the need to compete with a high degree of environmental noise when vocalizing. Finally, we observed that 98 +/- 1% of all USVs were produced in bouts, and this ratio was the most consistent feature of male mouse courtship USVs we measured, with all mice producing between 96.5 and 98.9% of their USVs in bouts (CV = 0.66%), further underscoring the multielement nature of mouse USVs.

USV series were found to display a stereotyped rhythmic structure where consecutive short USVs were preferred at the onset and offset of series, and consecutive long USVs were preferred series-internally; this organization was more rigid at the onset and offsets of bouts compared to bout-internal groups. Our present account of USV series rhythmic structure is consistent with a previous description of B6 male courtship USVs [21], underscoring the stability of this temporal organization across B6 mice. In addition to this gross rhythmic pattern of USV series, we also observed consistent fine-scale temporal structure exhibited by individual USVs in series. Specifically, USV duration at series onset and offset was found to be significantly reduced compared to series-internal USVs (*e.g*. a long USV at series onset is shorter than a long USV series-internally). The fine-scale temporal features of individual USVs were also found to be affected by the temporal features of adjacent USVs, such that long USVs were shorter when adjacent to short USVs and short USVs were longer when adjacent to long USVs. It is important to note that both the gross-scale and fine-scale temporal organization of USV series described in this study was highly probabilistic in nature, and not comparable to the rule-like syntactic structure observed in human speech or the vocalizations of many bird species (*e.g*. zebra finches).

To our knowledge, this is the first detailed examination of the temporal organization of mouse USV series. However, previous studies have examined the organization of USVs containing different numbers of pitch jumps (instantaneous nonlinear changes in dominant frequency) within USV series. These studies have consistently reported that USVs with no pitch jumps are most commonly produced at series onset and offset, and USVs containing multiple pitch jumps tend to occur series-internally [12, 94, 95]. We found that there was a significant linear correlation between the number of pitch jumps in a USV (instantaneous dominant frequency changes of 10 kHz or more) and USV duration in all 19 adult mice used for this study (S11 Fig, S30 and S31 Tables). Therefore, as USVs lacking pitch jumps would tend to be short USVs and USVs containing pitch jumps would tend to be long USVs, we can deduce that the rhythmic pattern we describe in this study is consistent with previous reports of USV series structure.

### Vocal development

The gross spectrotemporal structure of adult courtship USVs was found to develop continuously from birth and stabilize by P50. Specifically, the duration of pup isolation USVs was found to decrease until it approached the duration of short USVs in juvenile courtship vocalizations; the duration of juvenile short and long USVs then increased with age until P50. Similarly, the IVI duration of pup USVs was found to decrease and approach the IVI duration observed in juvenile courtship USVs, which varied little between the juvenile and adult age groups. The frequency of pup USVs also increased until it approximated the frequency of juvenile short USVs, and both short and long USV frequency decreased until P50. In general, both USV spectrotemporal structure and call rates vary little after P50, suggesting vocal development is mostly complete by this age, consistent with a previous study examining vocal development in *Foxp2* heterozygous knockout male mice and their wildtype B6 littermates [21].

As the spectrotemporal features of pup isolation calls approach juvenile courtship USVs, it suggests that pup USV and adult USV production may not be independent of one another. In support of this hypothesis, we found that pup USV duration and pup IVI duration was significantly correlated with USV duration and IVI duration in juvenile mice. Therefore, mice appear to be similar to marmosets and several species of bats, whose infant calls slowly develop into adult calls (bats [30, 134, 135]; marmosets [136–138]), but not neotropical singing mice, whose adult multielement songs appear *de novo* in juvenile animals and seem largely unrelated to the production of their infant calls [27]. Further research is necessary to establish the mechanisms of vocal ontogeny in mice, and whether this development involves learning of any kind (*e.g*. experience-based motor learning or non-template vocal learning) or is simply peripheral in nature. Furthermore, why long USVs appear to arise *de novo* in male courtship vocalizations is unknown.

Our observations regarding pup vocal development are partially consistent with results of previous studies examining the relationship between pup and adult calls. For example, Hahn *et al*. (1998) reported that both pup USV duration and frequency decrease as a function of age in multiple laboratory mice strains [139]. In addition, Liu *et al*. (2003) found that pup call interval in CBA/CaJ mice decreased with age and approached adult levels [100], which we also observed indirectly as both pup USV duration and IVI duration decreased over time; however, Liu and colleagues also reported that USV duration increased with age in pups and did not approach adult-like values [100]. Conversely, Grimsley *et al*. (2011) examined vocal development in CBA/CaJ mice, and found that overall pup USV duration declined only slightly while USV duration decreased over time and approached adult-like values in several, but not all, of the USV subtypes which the authors defined using spectrotemporal features; this study also reported a general overall decrease in pup USV frequency with age, with pup USVs having a higher peak frequency compared to those produced by adults [1]. However, Liu and colleagues did not report a consistent change in peak frequency in pup USVs over time, and further reported that pup USV peak frequency was lower than the peak frequency of adult USVs [100]. The reasons for the discrepancies between these two studies in relation to each other as well as our own study are unclear, but may be the result of strain differences or dissimilar methodologies. For example, we examined pup and adult USVs from the same group of male mice from birth into adulthood, and specifically tracked the development of each mouse individually rather than as a group. However, Grimsley *et al*. (2011) assessed male and female pup isolation USVs from P5 to P13 and adult vocalizations produced by both female and males mice between P91 and P140 [1], and Liu *et al*. (2003) examined female and male pups from P5 to P12 and adult courtship USVs produced by male mice between P42 and P54 [100]. Furthermore, as the isolation USVs produced by female and male pups have been shown to differ significantly [139], the usage of both male and female pups in previous studies may have contributed to the differences between reports of mouse vocal development.

### Temporal organization and call discrimination

The gross temporal properties of pup, adult female, and adult male USVs were found to differ dramatically. Specifically, male mice were found to produce both short and long USVs and call intervals, while female mice produced mostly short USVs with short call intervals, and pups produced mostly short USVs with long call intervals. Using only three temporal criteria reflecting the largest temporal distinctions between call types, we designed a simple linear classifier to discriminate groups of USVs (in the sense of Fig 3) produced by pups (all ages), adult males, and adult females. The classifier correctly identified 87.5 +/- 1.9% of pup USVs, 83.2 +/- 1.8% of adult female USVs, and 67.5 +/- 3.3% of adult male USVs when a single group was considered, and correctly identified over 90% of calls after considering three groups of pup and female USVs, and seven groups of adult male USVs. The median pup, female, and male group duration, calculated across all calls considered for the classifier analysis, was found to be 0.407, 0.247, and 0.445 seconds (transformed from log seconds; S32 Table), respectively; therefore, assuming average group durations and a typical IGI duration of 0.2 seconds, less than 5 seconds of group production would be required for 90% of calls to be correctly discriminated.

The success of this classifier is comparable to a previous analysis by Liu *et al*. (2003) that found individual CBA/CaJ pup and adult male USVs could be correctly discriminated using call interval alone over 80% of the time; considering USV duration and peak frequency in addition to call interval increased the rate of correct discrimination to 97.4% [100]. These results further underscore the importance of call temporal structure in the perception of mouse USVs, but also suggest additional spectrotemporal information is informative and may be utilized in call discrimination. For example, in agreement with previous reports [1, 100], we observed that pup USVs differ substantially from adult USVs in the frequency domain; therefore USV frequency may provide supplemental information differentiating pup and adult USVs. Additionally, we also observed that male mice produced significantly longer USV groups than female mice (p < 0.0001, one-way Kruskal-Wallis test; S32 Table), further distinguishing the two USV types. Similar sex differences in series length has also been observed in Alston’s brown mouse [23], suggesting that multielement call duration may be used by multiple species of rodent to differentiate male calls from female calls.

Although spectral features may provide redundant information that aids in the discrimination of USV subtypes, it is clear that the information provided by the temporal features dominates during USV perception in mice. For example, female mice approach pup USVs, 40-60 kHz bandpass noise, and ultrasonic tone bursts at identical rates as long as the temporal structure is within the range observed for actual pup calls [22, 78]. Furthermore, female mice will approach synthetic pup calls with naturalistic spectral features only if their timing is also naturalistic [77]. Therefore, temporal features alone are necessary and largely sufficient for at least pup call perception, while spectral features are not. This dependence of call discrimination on temporal features may represent an adaptation that takes specific advantage of the regular temporal structure exhibited by repetitive USV series, as illustrated by this study and others. For example, Mahrt *et al*. (2013) observed considerable variability in the acoustic features of individual male courtship USVs across CBA/CaJ mice, but found IVI duration to highly stable across individuals and experimental manipulations [140]. Similarly, a study examining spectrotemporal variability in pup isolation USVs and male courtship USVs found that USV duration was the most consistent feature across recording sessions and individuals [141]. Furthermore, the size of neural representations in rat auditory cortex were found to increase for tones repeated at the same call interval as rat pup isolation USVs [26], suggesting that the rodent auditory cortex has adapted to facilitate the perception of USVs by leveraging their consistent temporal structure.

In conclusion, we find that the gross temporal structure of USV series is sufficient for call discrimination in mice, in agreement with the results of previous studies [22, 77, 78, 100], and that call spectral structure most likely serves as a source of redundant information to facilitate perception. However, it is worth noting that the fine-scale acoustic structure of individual USVs may be utilized by mice when discriminating between USVs of a single type. For example, substantial differences are observed in the spectrotemporal structure of male courtship USVs across strains of mice, and female mice appear to be sensitive to these differences as they approach the USVs of some strains at higher rates than others [81]. Similarly, the spectral structure of male courtship USVs in wild mice was found to be more similar among kin than unrelated mice [142], and female mice approach non-kin USVs at higher rates that those produced by kin [143]. Mouse pups have also been shown to prefer the USVs of their mother in comparison to other those of other females, and likewise female mice respond more quickly to the isolation calls of their own pups than unrelated pups [144].

Further studies are required to determine the precise spectrotemporal features driving the discrimination of USVs within a single call category, as little is known about the acoustic features of individual USVs that are perceived by mice. In general, mice have been observed to discriminate USVs that are spectrotemporally distinct from one another [145], but are largely unable to discriminate USVs that overlap in frequency space, for example upsweeps and downsweeps are not well discriminated by mice [146]. Furthermore, measurable differences in the acoustic structure of animal vocalizations are often unimportant to the animals themselves; for example, certain subpopulations of grey seals (*Halichoerus grypus*) do not discriminate the distinct vocalizations of their own pup from those produced by other pups [147, 148], bottlenose dolphins (*Tursiops truncatus*) do not use the acoustic features of non-signature whistle calls to determine conspecific identity [149], and female red deer (*Cervus elaphus*) do not display a preference for pitch differences in the roars of bucks [150].

## Conclusion

Male B6 mice produced two USV classes that exhibit distinct temporal features and articulatory properties. Individual USVs were consistently produced in hierarchical multielement series (bouts) containing a single class of subseries (groups); these USV series were produced with a stereotyped temporal structure that was highly consistent across mice. The gross spectrotemporal features of male USVs were also observed to develop continuously from birth and stabilize by P50. Finally, USVs produced by pup, adult male, and female mice were found to display dissimilar temporal properties that were sufficient to mathematically discriminate between call types, suggesting that call timing is important for USV perception. This study furthers our understanding of USV production in mice and its relation to multielement call production in other animals, and furthermore provides an important point of reference for future studies assessing the USVs of B6 mice, a commonly used genetic background for mouse models of human diseases.

## Supporting information

S1 Fig Male mice consistently produce a high call rate by P50. Call rate during individual recording sessions for each of the 19 mice from the first recording session (P17-P25) until P85. Each line represents an individual mouse.
(TIF)

S2 Fig Short and long USVs display different onset and offset coordination phases. **(a)** Onset coordination phase for short and long USVs (0 degrees indicates phonation begins at exhalation onset, 360 degrees indicates phonation begins at inhalation onset); short USVs had a significantly greater onset coordination phase than long USVs (p = 0.001, Wilcoxon match-pairs signed rank test). **(b)** Offset coordination phase for short and long USVs (0 degrees indicates phonation ends at inhalation onset, 360 degrees indicates phonation ends at exhalation onset); short USVs had a significantly greater offset coordination phase than long USVs (p < 0.0001, paired t-test). Error bars indicate the mean across animals +/-1 standard deviation, and values for individual mice are represented by the 11 overlaid points. See S2 and S3 Tables for additional statistical details for the comparisons in (a) and (b), respectively.
(TIF)

S3 Fig Long USV probability as a function of preceding IVI duration. The average normalized probability of observing a long USVs as a function of preceding ISI duration. Red upper error bars indicate a significant difference in probability compared to IVIs (75-100 ms reference), and black lower error bars indicate a significant difference compared to IBIs (500+ ms reference) (p < 0.05, repeated measures one-way ANOVA with Dunnett’s correction for multiple comparisons; see S7 Table for additional statistical details). Error bars indicate the mean across all 19 animals +/-1 standard error.
(TIF)

S4 Fig The number of USVs/groups per series is consistent across mice. **(i)** The median number of USVs per group and bout in all 19 adult mice, and **(ii)** the median number of groups per bout in all 19 adult mice. Bars represent the population median, and values for individual mice are represented by the 19 overlaid points. See S10 Table for additional statistical details.
(TIF)

S5 Fig Raw transition probabilities across mice. The average conditional probability of each transition across all 19 adult mice; error bars indicate the mean across animals +/-1 standard deviation, and values for individual mice are represented by the 19 overlaid points. Additional statistical details are presented in S11 Table.
(TIF)

S6 Fig Supplementary analysis of fine-scale temporal structure. >**(a)** Average normalized duration of short USVs series-finally. See S14 and S15 Tables for statistical details; summary statistics are presented in S17 Table. **(b)** Average normalized durations for short USVs adjacent to either 1 or 2 long USVs. Statistical details are presented in S18 and S19 Tables; summary statistics are presented in S21 Table. **(c)** Average normalized durations for series-initial and series-final USVs adjacent to USVs of a different class across all adult mice. See S22 and S23 Tables for statistical details, and S24 Table for summary statistics. Error bars indicate the mean across animals +/-1 standard deviation, and values for individual mice are represented by the 19 overlaid points. The labelling scheme in this figure is identical to the one used in Fig 6 and described in the corresponding figure legend.
(TIF)

S7 Fig Temporal features of adult courtship USVs develop through maturation. **(a)** Mean Ashman’s D scores of courtship USV duration distributions across all 19 mice over time. Scores significantly increase with age. **(b)** Average proportion of long USVs produced over time across all mice. Production of long USVs significantly increased with age. **(c)** Mean short and long USV duration variance across mice over time; only long USV variance significantly increased with age. **(d)** Mean weighted frequency of pup USVs at P12-P16 did not correlate with the weighted frequency of short or long USVs at P17-P34. In (a-c), significance is assessed with repeated-measures one-way ANOVAs with Tukey’s correction of multiple comparisons; see S27 and S28 Tables for statistical details. Summary statistics are presented in S25 and S26 Tables. Error bars indicate the mean value across animals +/- 1 standard deviation. Individual lines indicate the values for each mouse.
(TIF)

S8 Fig Minimum IVI decreases during early postnatal development. Histogram of silent intervals longer than 8 ms in all recording sessions of all 11 pups from **(a)** P0-P3, **(b)** P4-P5, **(c)** P6-P7, **(d)** P8-P9, **(e)** P10-P11, and **(f)** P12-16. Minimum IVI threshold for each age group is indicated on the respective histograms.
(TIF)

S9 Fig Maximum IVI durations for adult and pup age groups. Histograms of the pooled distributions of intervening silent interval durations across all 11 pups and 19 adult mice at **(a)** P0-P3, **(b)** P4-P5, **(c)** P6-P7, **(d)** P8-P9, **(e)** P10-P11, **(f)** P12-P16, **(g)** P17-P34, **(h)** P35-P49, **(i)** P50-P65, **(j)** P66-P95. The width of the distribution in 10 ms bins at 75% maximum height (red horizontal bars) was determined to assign a maximum IVI duration for each age group, which was defined as the upper limit of the 75% maximum height range (gray vertical lines). Data falling within the upper limit of the width at 75% maximum height and the minimum IVI value (see S8 Fig) were fit with a Gaussian to determine the mean and variance of each IVI duration distribution for descriptive purposes.
(TIF)

S10 Fig Female USVs produced in isolation or in social contexts have similar temporal properties. **(a)** Histogram of USV duration from all recordings of female mice in (a.i) isolation or (a.ii) in female-female dyads. **(b)** Histogram of ISI duration from all recordings of female mice in (b.i) isolation or (b.ii) in female-female dyads. **(c)** Histogram of pooled ISI durations across all recordings of adult female mice. The width of the distribution in 10 ms bins at 75% maximum height (red horizontal bars) was determined to assign a maximum IVI duration value, which was defined as the upper limit of the 75% maximum height range (gray vertical line). Data falling within the upper limit of the width at 75% maximum height and the minimum IVI value (40 ms) were fit with a Gaussian to determine the mean and variance of the IVI duration distribution for descriptive purposes.
(TIF)

S11 Fig Number of pitch jumps is correlated to USV duration. **(a)** Scatterplot showing the number of pitch jumps in a USV as function of USV duration in one adult mouse. The linear regression line of the relationship is plotted in red; the slope, intercept, and correlation coefficient of this line is also reported in red. **(b)** Linear regression lines for all 19 adult male mice, with each thin line representing an individual animal. The thick black line is the average regression line; its slope, intercept, and correlation coefficient is also reported in black. All mice displayed a significant linear relationship between number of pitch jumps in a USV and USV duration (all p < 0.0001, see S30 Table for statistical details). See S31 Table for summary statistics.
(TIF)

S1 Table Summary statistics for USV duration distribution fits (n = 19 mice). (PDF)

S2 Table Statistics for onset lag comparisons (n = 11 mice). (PDF)

S3 Table Statistics for offset lag comparisons (n = 11 mice). (PDF)

S4 Table Statistics for offset coordination linear fit comparisons (n = 11 mice). (PDF)

S5 Table Offset coordination linear fit statistics (n = 11 mice). (PDF)

S6 Table Multiple comparisons statistics for long USV probability as a function of following ISI duration (n = 19 mice). (PDF)

S7 Table Multiple comparisons statistics for long USV probability as a function of preceding ISI duration (n = 19 mice). (PDF)

S8 Table Summary statistics for series proportions (n = 19 mice). (PDF)

S9 Table Summary statistics for series durations (n = 19 mice). (PDF)

S10 Table Summary statistics for number of USVs per series and groups per bout (n = 19 mice). (PDF)

S11 Table Summary statistics for raw transition probabilities (n = 19 mice). (PDF)

S12 Table Summary statistics for preference scores (n = 19 mice). (PDF)

S13 Table Multiple comparisons statistics for preference scores (n = 19 mice). (PDF)

S14 Table Descriptive statistics for series onset and offset temporal regularities. (PDF)

S15 Table Multiple comparisons statistics for series onset and offset temporal regularities, short USVs (one way Kruskal-Wallis test). (PDF)

S16 Table Multiple comparisons statistics for series onset and offset temporal regularities, long USVs (one-way Kruskal-Wallis test). (PDF)

S17 Table Summary statistics for series onset and offset normalized durations (n = 19 mice). (PDF)

S18 Table Descriptive statistics for adjacency-related temporal regularities. (PDF)

S19 Table Multiple comparisons statistics for adjacency-related temporal regularities, short USVs (one-way Kruskal-Wallis test). (PDF)

S20 Table Multiple comparisons statistics for adjacency-related temporal regularities, long USVs (one-way Kruskal-Wallis test). (PDF)

S21 Table Summary statistics for adjacency-related normalized durations (n = 19 mice). (PDF)

S22 Table Descriptive statistics for combined fine-scale temporal regularities. (PDF)

S23 Table Multiple comparison statistics for combined fine-scale temporal regularities (one-way Kruskal-Wallis test). (PDF)

S24 Table Summary statistics for combined fine-scale normalized durations (n = 19 mice). (PDF)

S25 Table Summary statistics for pup vocal development (n = 11, *n = 9 mice). (PDF)

S26 Table Summary statistics for adult vocal development (n = 19 mice). (PDF)

S27 Table Statistics for pup age group comparisons. (PDF)

S28 Table Statistics for adult age group comparisons. (PDF)

S29 Table Summary statistics for USV group classifications (100 iterations of 250 random groups each). (PDF)

S30 Table Pitch jumps per USV-USV duration linear fit statistics (n = 19 mice). (PDF)

S31 Table Summary statistics for pitch jump-USV duration linear regressions (n = 19 mice). (PDF)

S32 Table Summary statistics and multiple comparisons for group durations. (PDF)
