# Supplementary figures and images for: The temporal organization of mouse ultrasonic vocalizations
Source: PLoS One. 2018 Oct 30;13(10):e0199929. doi: 10.1371/journal.pone.0199929 (PMC6207298; doi:10.1371/journal.pone.0199929)

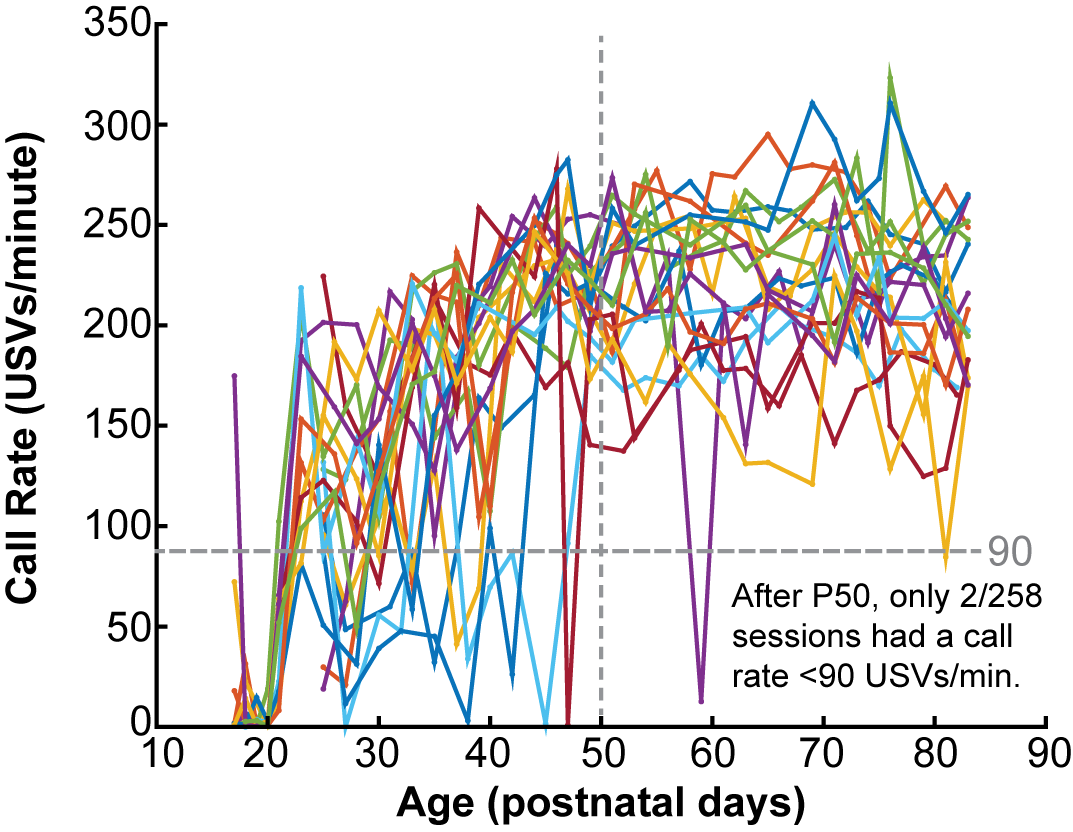

Supplement: S1 Fig — Call rate during individual recording sessions for each of the 19 mice from the first recording session (P17-P25) until P85. Each line represents an individual mouse. (TIF) [file pone.0199929.s001.tif]

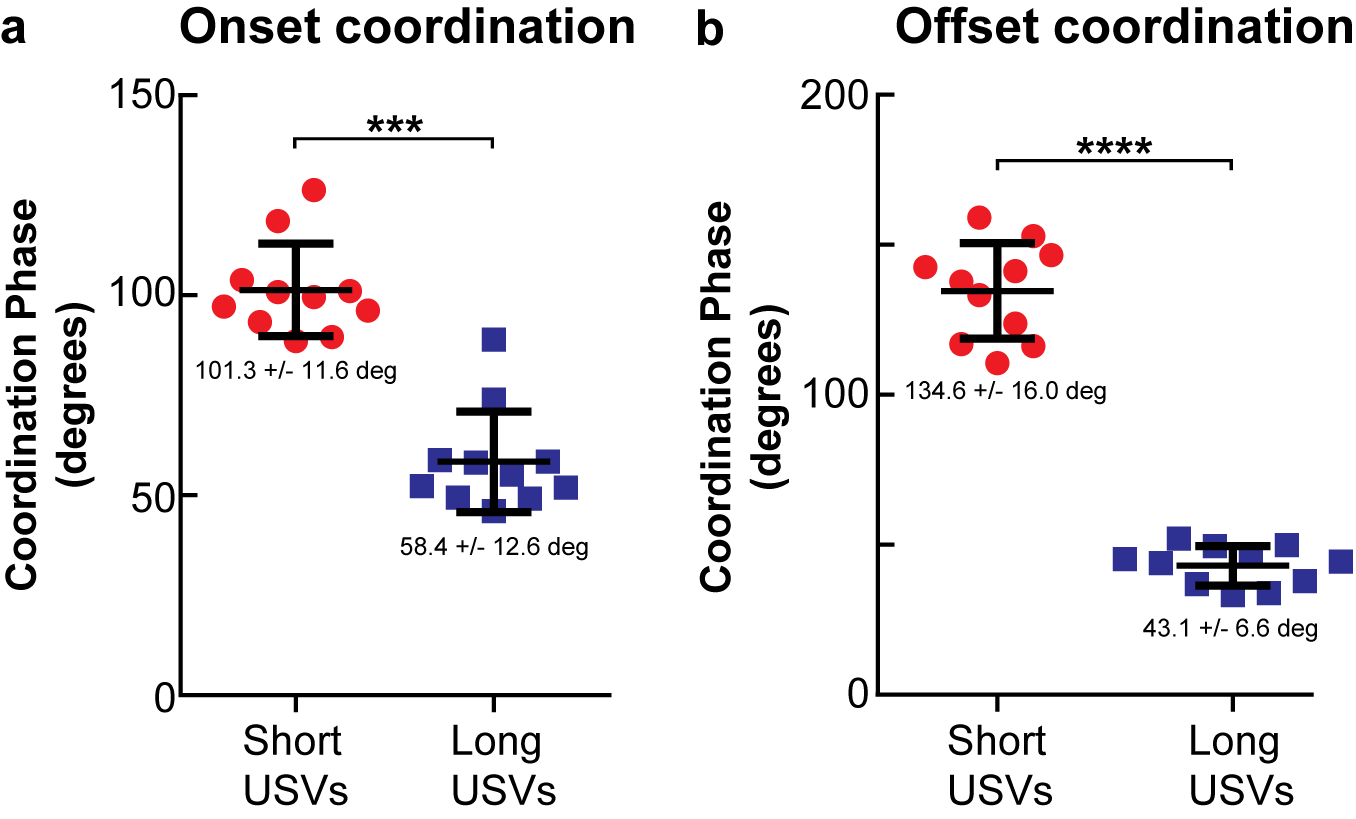

Supplement: S2 Fig — (a) Onset coordination phase for short and long USVs (0 degrees indicates phonation begins at exhalation onset, 360 degrees indicates phonation begins at inhalation onset); short USVs had a significantly greater onset coordination phase than long USVs (p = 0.001, Wilcoxon match-pairs signed rank test). (b) Offset coordination phase for short and long USVs (0 degrees indicates phonation ends at inhalation onset, 360 degrees indicates phonation ends at exhalation onset); short USVs had a significantly greater offset coordination phase than long USVs (p < 0.0001, paired t-test). Error bars indicate the mean across animals +/-1 standard deviation, and values for individual mice are represented by the 11 overlaid points. See S2 and S3 Tables for additional statistical details for the comparisons in (a) and (b), respectively. (TIF) [file pone.0199929.s002.tif]

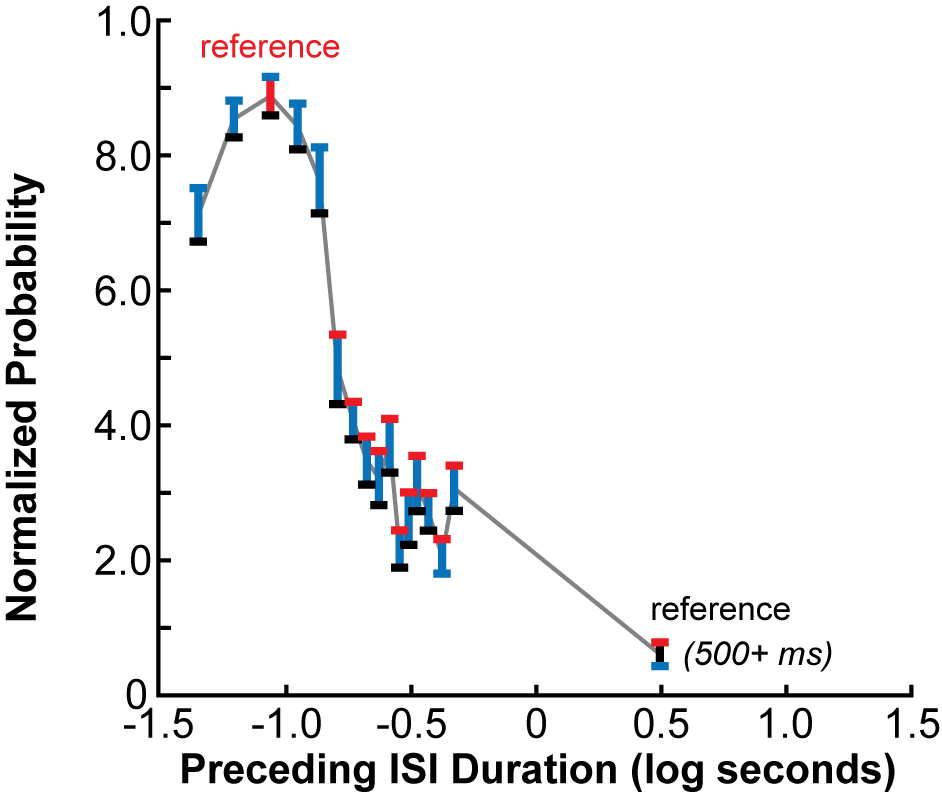

Supplement: S3 Fig — The average normalized probability of observing a long USVs as a function of preceding ISI duration. Red upper error bars indicate a significant difference in probability compared to IVIs (75-100 ms reference), and black lower error bars indicate a significant difference compared to IBIs (500+ ms reference) (p < 0.05, repeated measures one-way ANOVA with Dunnett’s correction for multiple comparisons; see S7 Table for additional statistical details). Error bars indicate the mean across all 19 animals +/-1 standard error. (TIF) [file pone.0199929.s003.tif]

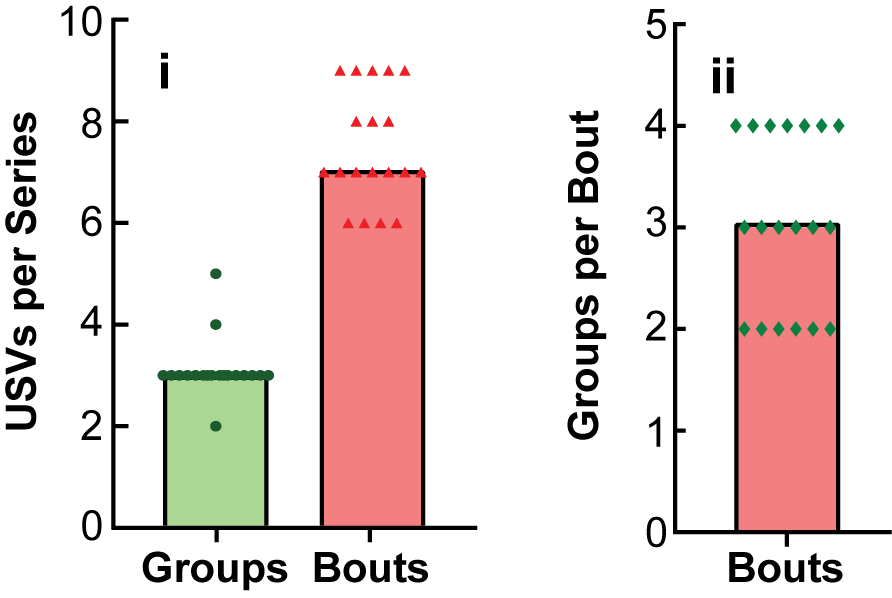

Supplement: S4 Fig — (i) The median number of USVs per group and bout in all 19 adult mice, and (ii) the median number of groups per bout in all 19 adult mice. Bars represent the population median, and values for individual mice are represented by the 19 overlaid points. See S10 Table for additional statistical details. (TIF) [file pone.0199929.s004.tif]

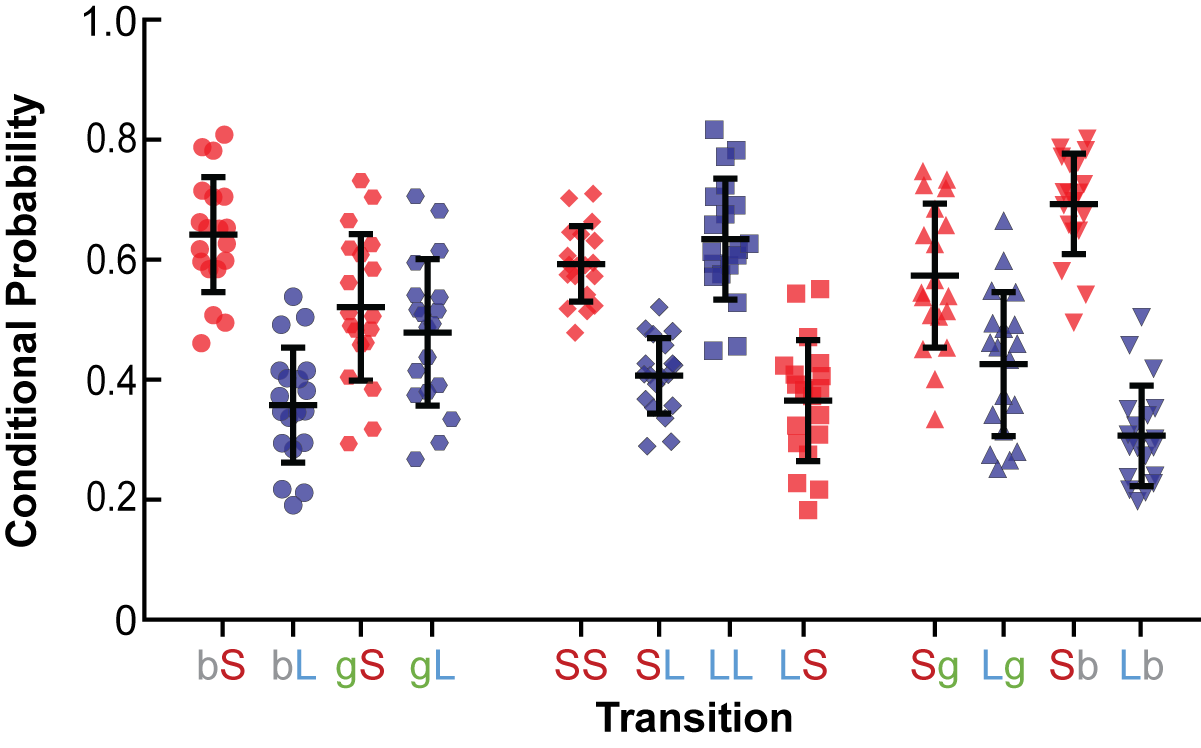

Supplement: S5 Fig — The average conditional probability of each transition across all 19 adult mice; error bars indicate the mean across animals +/-1 standard deviation, and values for individual mice are represented by the 19 overlaid points. Additional statistical details are presented in S11 Table. (TIF) [file pone.0199929.s005.tif]

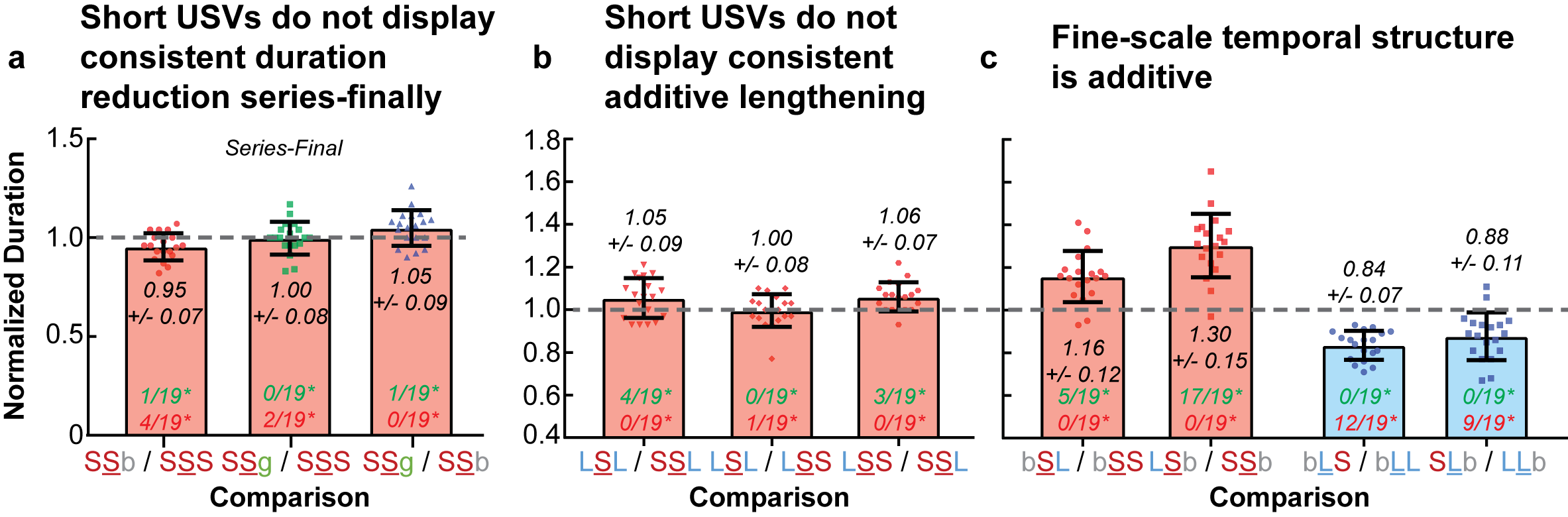

Supplement: S6 Fig — >(a) Average normalized duration of short USVs series-finally. See S14 and S15 Tables for statistical details; summary statistics are presented in S17 Table. (b) Average normalized durations for short USVs adjacent to either 1 or 2 long USVs. Statistical details are presented in S18 and S19 Tables; summary statistics are presented in S21 Table. (c) Average normalized durations for series-initial and series-final USVs adjacent to USVs of a different class across all adult mice. See S22 and S23 Tables for statistical details, and S24 Table for summary statistics. Error bars indicate the mean across animals +/-1 standard deviation, and values for individual mice are represented by the 19 overlaid points. The labelling scheme in this figure is identical to the one used in Fig 6 and described in the corresponding figure legend. (TIF) [file pone.0199929.s006.tif]

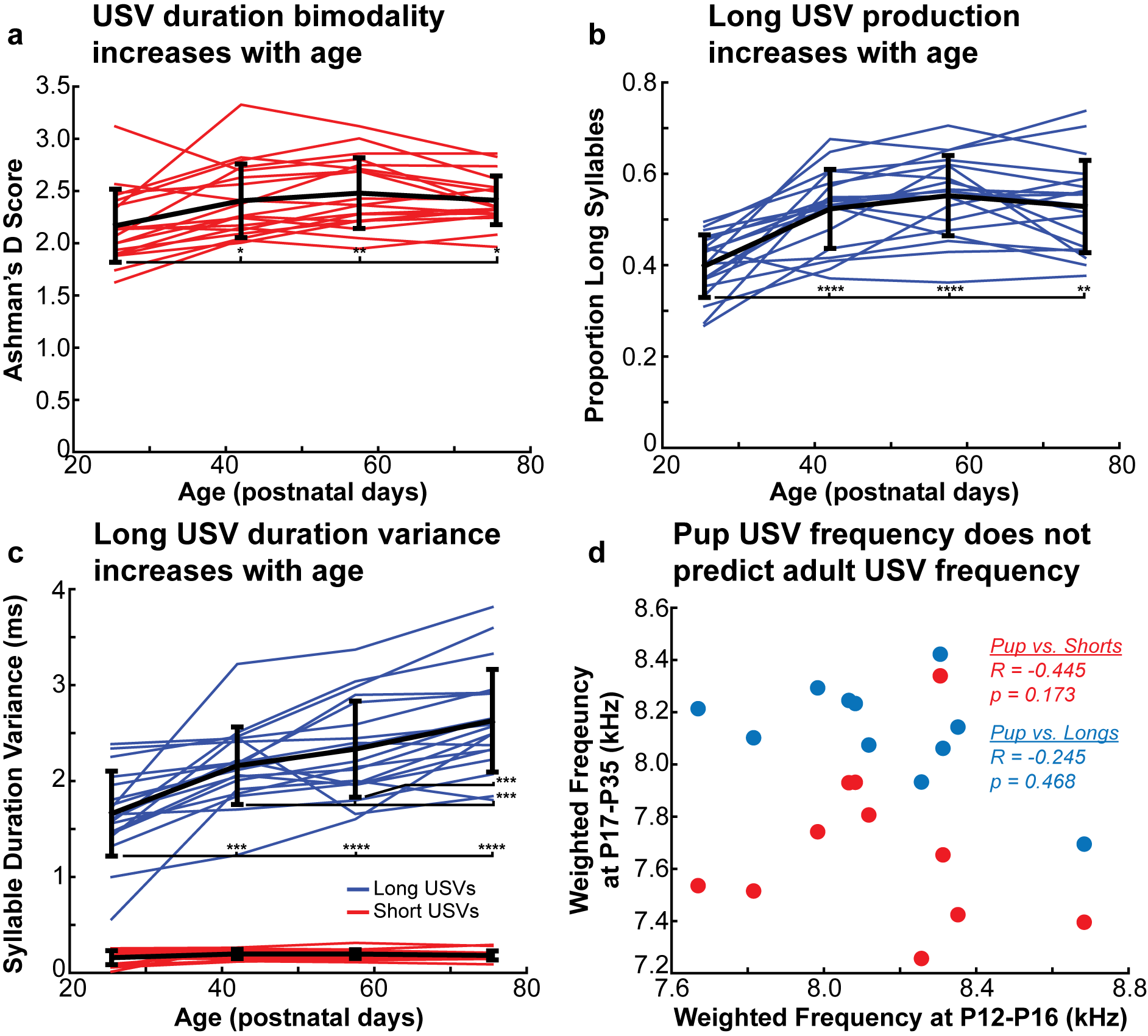

Supplement: S7 Fig — (a) Mean Ashman’s D scores of courtship USV duration distributions across all 19 mice over time. Scores significantly increase with age. (b) Average proportion of long USVs produced over time across all mice. Production of long USVs significantly increased with age. (c) Mean short and long USV duration variance across mice over time; only long USV variance significantly increased with age. (d) Mean weighted frequency of pup USVs at P12-P16 did not correlate with the weighted frequency of short or long USVs at P17-P34. In (a-c), significance is assessed with repeated-measures one-way ANOVAs with Tukey’s correction of multiple comparisons; see S27 and S28 Tables for statistical details. Summary statistics are presented in S25 and S26 Tables. Error bars indicate the mean value across animals +/- 1 standard deviation. Individual lines indicate the values for each mouse. (TIF) [file pone.0199929.s007.tif]

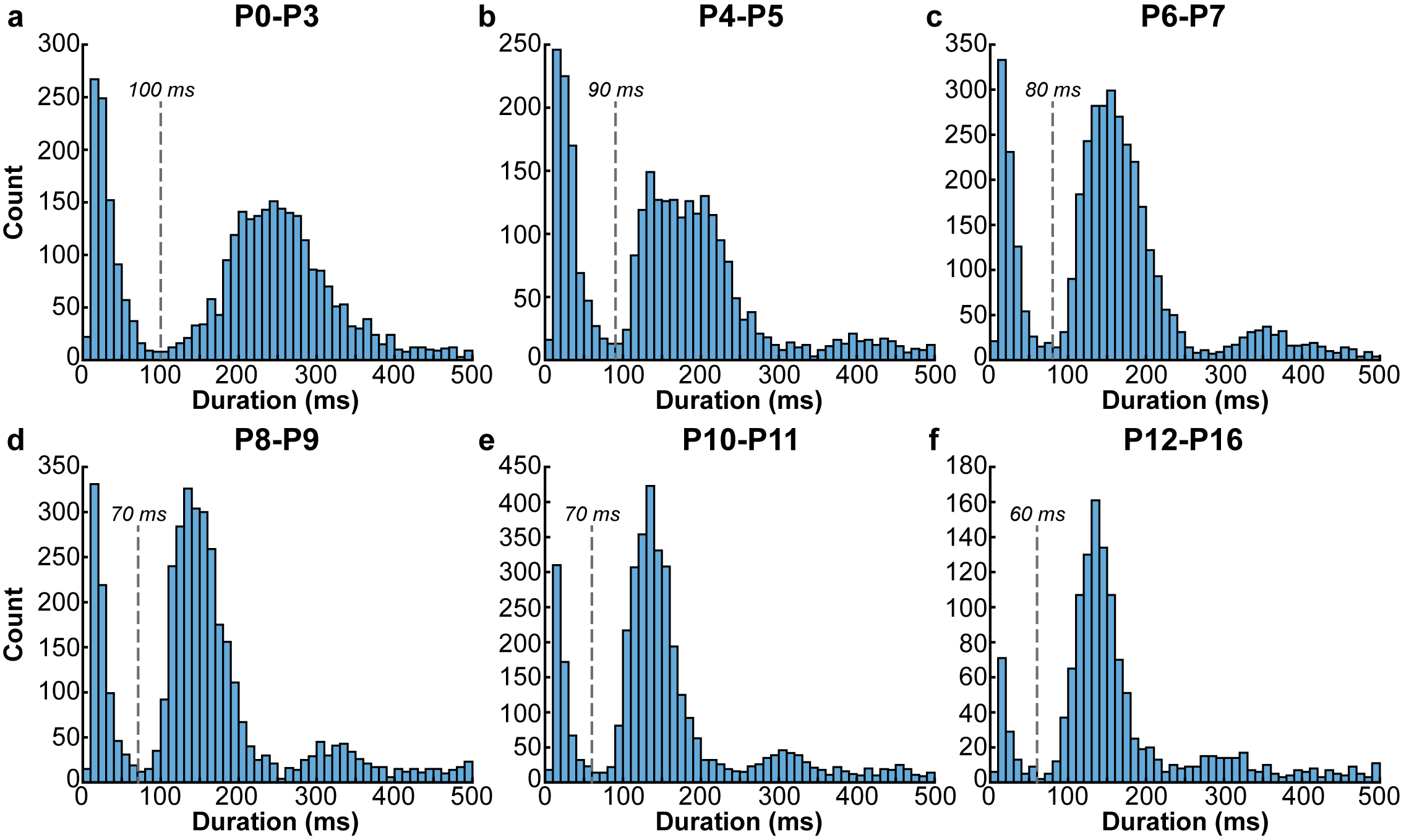

Supplement: S8 Fig — Histogram of silent intervals longer than 8 ms in all recording sessions of all 11 pups from (a) P0-P3, (b) P4-P5, (c) P6-P7, (d) P8-P9, (e) P10-P11, and (f) P12-16. Minimum IVI threshold for each age group is indicated on the respective histograms. (TIF) [file pone.0199929.s008.tif]

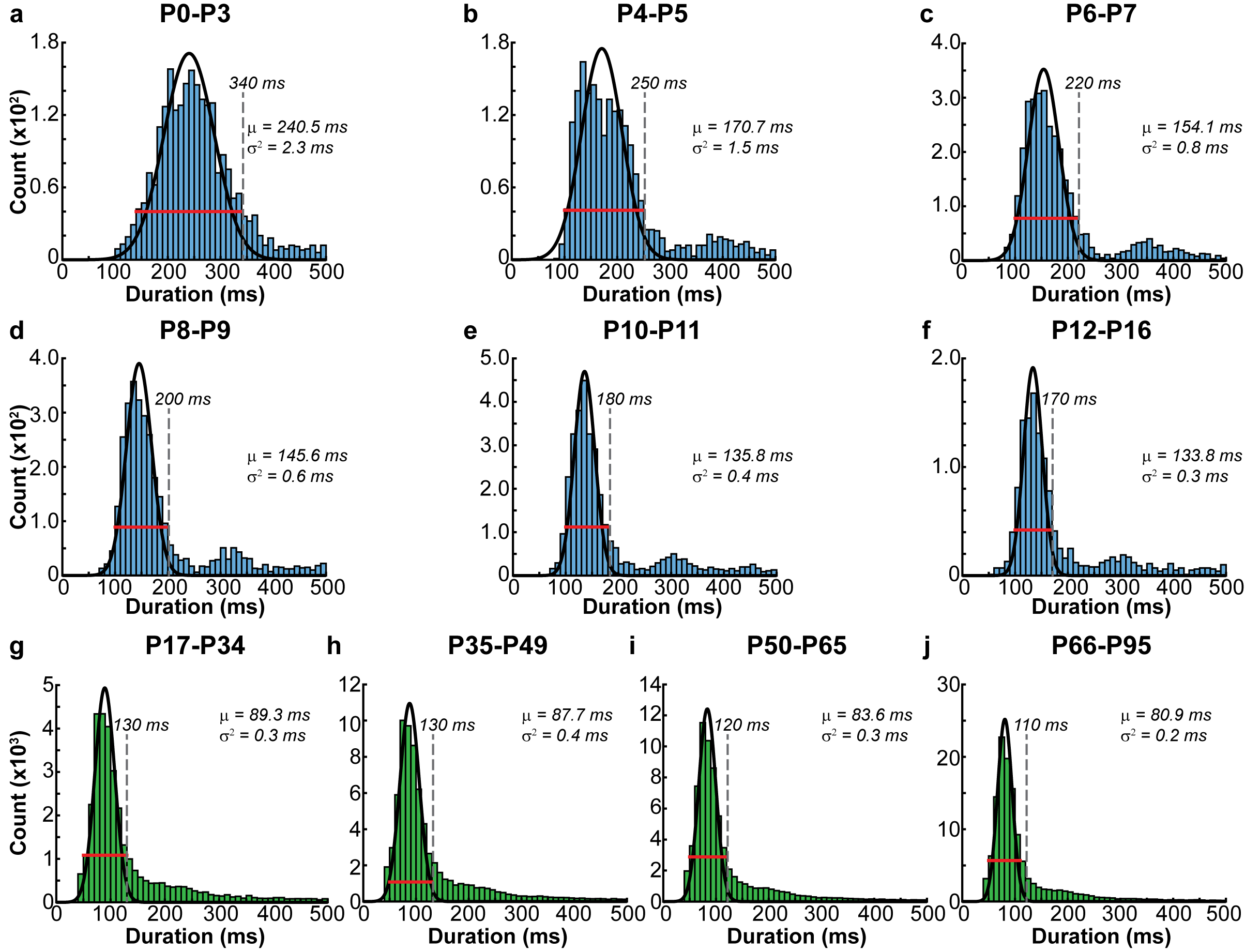

Supplement: S9 Fig — Histograms of the pooled distributions of intervening silent interval durations across all 11 pups and 19 adult mice at (a) P0-P3, (b) P4-P5, (c) P6-P7, (d) P8-P9, (e) P10-P11, (f) P12-P16, (g) P17-P34, (h) P35-P49, (i) P50-P65, (j) P66-P95. The width of the distribution in 10 ms bins at 75% maximum height (red horizontal bars) was determined to assign a maximum IVI duration for each age group, which was defined as the upper limit of the 75% maximum height range (gray vertical lines). Data falling within the upper limit of the width at 75% maximum height and the minimum IVI value (see S8 Fig) were fit with a Gaussian to determine the mean and variance of each IVI duration distribution for descriptive purposes. (TIF) [file pone.0199929.s009.tif]

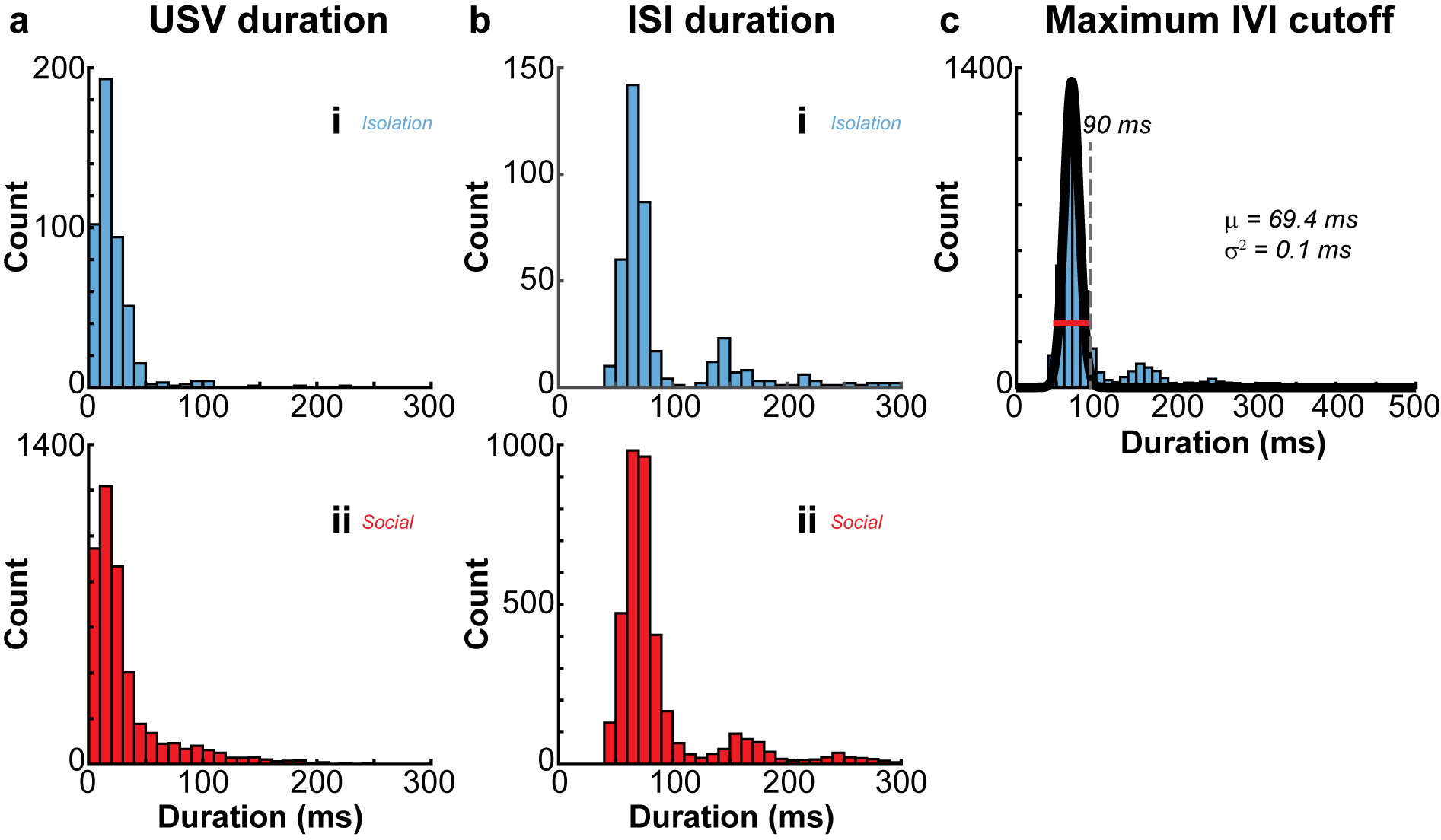

Supplement: S10 Fig — (a) Histogram of USV duration from all recordings of female mice in (a.i) isolation or (a.ii) in female-female dyads. (b) Histogram of ISI duration from all recordings of female mice in (b.i) isolation or (b.ii) in female-female dyads. (c) Histogram of pooled ISI durations across all recordings of adult female mice. The width of the distribution in 10 ms bins at 75% maximum height (red horizontal bars) was determined to assign a maximum IVI duration value, which was defined as the upper limit of the 75% maximum height range (gray vertical line). Data falling within the upper limit of the width at 75% maximum height and the minimum IVI value (40 ms) were fit with a Gaussian to determine the mean and variance of the IVI duration distribution for descriptive purposes. (TIF) [file pone.0199929.s010.tif]

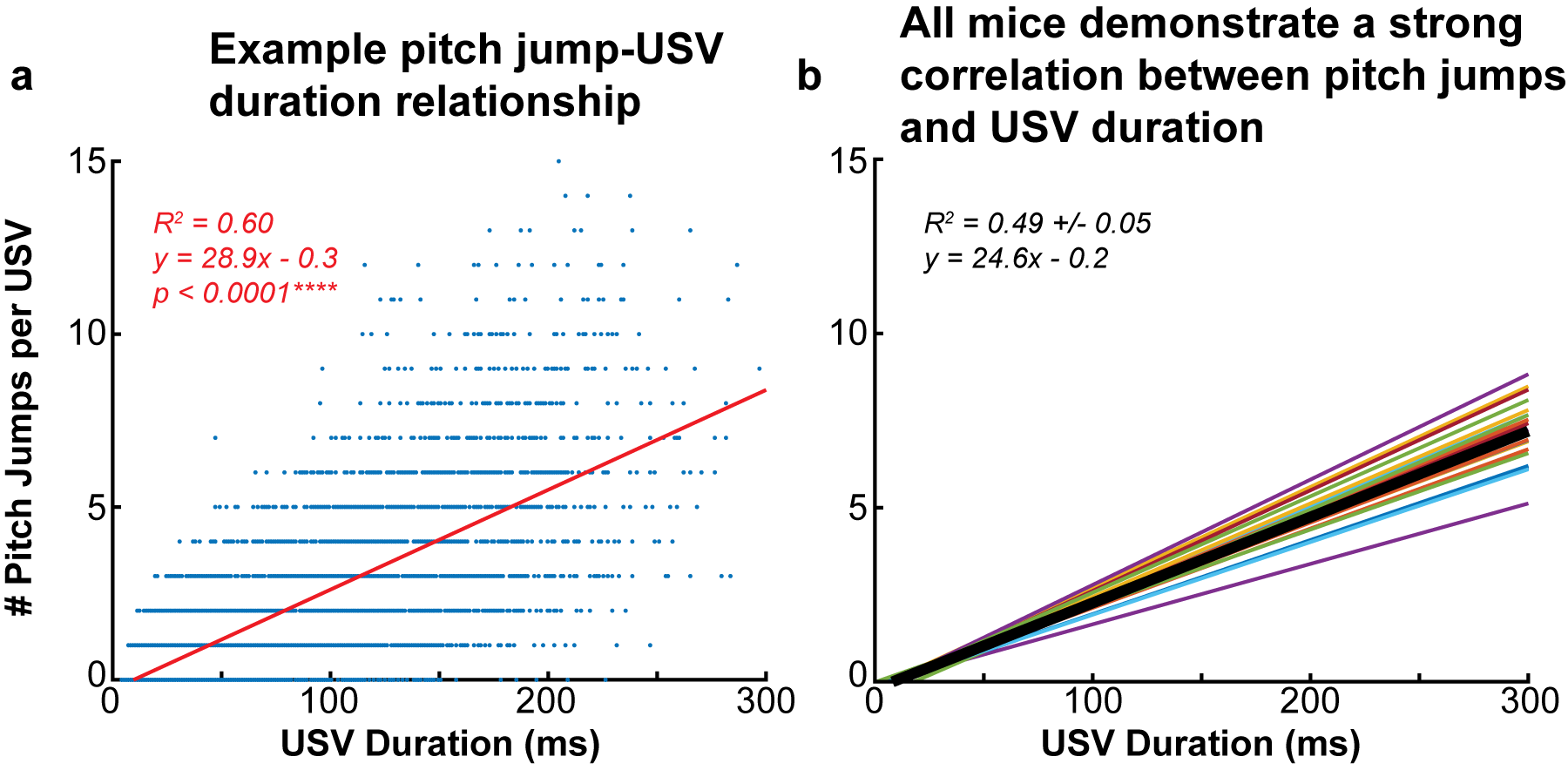

Supplement: S11 Fig — (a) Scatterplot showing the number of pitch jumps in a USV as function of USV duration in one adult mouse. The linear regression line of the relationship is plotted in red; the slope, intercept, and correlation coefficient of this line is also reported in red. (b) Linear regression lines for all 19 adult male mice, with each thin line representing an individual animal. The thick black line is the average regression line; its slope, intercept, and correlation coefficient is also reported in black. All mice displayed a significant linear relationship between number of pitch jumps in a USV and USV duration (all p < 0.0001, see S30 Table for statistical details). See S31 Table for summary statistics. (TIF) [file pone.0199929.s011.tif]
