# Supplementary material for: The temporal organization of mouse ultrasonic vocalizations
Source: PLoS One. 2018 Oct 30;13(10):e0199929. doi: 10.1371/journal.pone.0199929 (PMC6207298; doi:10.1371/journal.pone.0199929)
Supplement: S1 Table — (PDF) [file pone.0199929.s012.pdf]

Table S1. Summary statistics for USV duration distribution fits (n = 19 mice)					
Data Set	Mean	Standard Deviation	Coefficient of Variation	D'Agostino & Pearson Normality Test	
				P-Value (α = 0.009, Sidak Correction)	*K2*
Short USV Mean Duration	26.8 ms	3.6 ms	13.61%	0.0491	6.027
Long USV Mean Duration	116.7 ms	19.4 ms	16.66%	0.0158	8.296
Short-Long USV Cutoff Duration	54.6 ms	5.53 ms	10.13%	0.7023	0.707
Ashman's D Score	2.38	0.26	10.83%	0.3967	1.849
Short USV Proportion	0.45	0.09	20.73%	0.7841	0.486
Long USV Proportion	0.55	0.09	17.27%	0.7841	0.486
