# Supplementary material for: The temporal organization of mouse ultrasonic vocalizations
Source: PLoS One. 2018 Oct 30;13(10):e0199929. doi: 10.1371/journal.pone.0199929 (PMC6207298; doi:10.1371/journal.pone.0199929)
Supplement: S2 Table — (PDF) [file pone.0199929.s013.pdf]

Table S2. Statistics for onset lag comparisons (n = 11 mice)								
Data Set	Median	Mean	Standard Error	Coefficient of Variation	D'Agostino & Pearson Normality Test		Wilcoxon match-pairs signed rank test (two-tailed)	
					P-Value (α *= 0.013, Sidak Correction)*	*K2*	*W*	*P-value*
Short USV Exhalation Onset-Phonation Onset Lag	18.9 ms	20.1 ms	1.0 ms	16.88%	0.0844	4.943	-60	>0.005**
Long USV Exhalation Onset-Phonation Onset Lag	17.2 ms	18.7 ms	1.2 ms	21.22%	0.0038**	11.130		
Short USV Exhalation Onset-Phonation Onset Coordination	99.6 degrees	101.3 degrees	3.5 degrees	11.42%	0.0911	4.793	-66	0.001***
Long USV Exhalation Onset-Phonation Onset Coordination	48.4 degrees	58.4 degrees	3.8 degrees	21.50%	0.0074**	9.814		
