# Supplementary material for: The temporal organization of mouse ultrasonic vocalizations
Source: PLoS One. 2018 Oct 30;13(10):e0199929. doi: 10.1371/journal.pone.0199929 (PMC6207298; doi:10.1371/journal.pone.0199929)
Supplement: S3 Table — (PDF) [file pone.0199929.s014.pdf]

Table S3. Statistics for offset lag comparisons (n = 11 mice)

Data Set	Median	Mean	Standard Error	Coefficient of Variation	D'Agostino & Pearson Normality Test		Paired t-test (two tailed)	
					P-Value (α = 0.013, Sidak Correction)	*K2*	*t, df*	*P-value*
Short USV Inhalation Onset-Phonation Offset Lag	27.8 ms	26.8 ms	1.1 ms	14.13%	0.5788	1.094	t = 14.19, df = 10	<0.0001***
Long USV Inhalation Onset-Phonation Offset Lag	15.9 ms	15.4 ms	0.7 ms	15.23%	0.4616	1.546		
Short USV Inhalation Onset-Phonation Offset Coordination	137.8 degrees	134.6 degrees	4.8 degrees	11.86%	0.5795	1.091	t = 27.01, df = 10	<0.0001***
Long USV Inhalation Onset-Phonation Offset Coordination	44.4 degrees	43.1 degrees	2.0 degrees	15.30%	0.4233	1.719		
