# Supplementary material for: The temporal organization of mouse ultrasonic vocalizations
Source: PLoS One. 2018 Oct 30;13(10):e0199929. doi: 10.1371/journal.pone.0199929 (PMC6207298; doi:10.1371/journal.pone.0199929)
Supplement: S4 Table — (PDF) [file pone.0199929.s015.pdf]

Table S4. Statistics for offset coordination linear fit comparisons (n = 11 mice)

Data Set	Mean	Standard Error	Coefficient of Variation	D'Agostino & Pearson Normality Test		Paired t-test (two tailed)	
				P-Value (α = 0.013, Sidak Correction)	*K2*	*t, df*	*P-value*
Short USV R^2	0.47	0.04	25.42%	0.2203	3.026	t = 6.551, df = 10	<0.0001****
Long USV R^2	0.15	0.02	52.80%	0.2187	3.040		
Short USV Slope	-2726 deg/sec	131.0 deg/sec	15.94%	0.2110	3.111	t = 18.59, df = 10	<0.0001****
Long USV Slope	-422.7 deg/sec	56.09 deg/sec	44.04%	0.7348	0.6163		
