# Supplementary material for: The temporal organization of mouse ultrasonic vocalizations
Source: PLoS One. 2018 Oct 30;13(10):e0199929. doi: 10.1371/journal.pone.0199929 (PMC6207298; doi:10.1371/journal.pone.0199929)
Supplement: S5 Table — (PDF) [file pone.0199929.s016.pdf]

Table S5. Offset coordination linear fit statistics (n = 11 mice)

Data Set	R^2 (Pearson)	Slope (deg/sec)	Standard Error (deg/sec)	F	DFn, DFd	P Value	Y-Intercept (deg)	Standard Error (deg)	n (USVs)
Mouse 9 - Short USVs	0.4123	-3245	97	1119	1, 1596	<0.0001	220.5	3.03	1598
Mouse 10 - Short USVs	0.4239	-2857	121.2	555.5	1, 755	<0.0001	212.7	2.582	757
Mouse 11 - Short USVs	0.4562	-2456	62.88	1526	1, 1819	<0.0001	208.1	1.838	1821
Mouse 12 - Short USVs	0.6602	-3123	48.21	4195	1, 2159	<0.0001	202.6	1.541	2161
Mouse 13 - Short USVs	0.5008	-2714	79.99	1151	1, 1147	<0.0001	204.4	2.177	1149
Mouse 14 - Short USVs	0.5693	-3150	84.49	1390	1, 1052	<0.0001	218.6	1.983	1054
Mouse 15 - Short USVs	0.4799	-2160	59.3	1327	1, 1438	<0.0001	178.3	1.904	1440
Mouse 16 - Short USVs	0.3745	-2144	74.63	825.1	1, 1378	<0.0001	176.7	2.039	1380
Mouse 17 - Short USVs	0.5542	-3120	116.1	722.3	1, 581	<0.0001	221.4	3.177	583
Mouse 18 - Short USVs	0.5396	-2880	64.86	1971	1, 1682	<0.0001	198.4	1.987	1684
Mouse 19 - Short USVs	0.2066	-2133	118.4	324.5	1, 1246	<0.0001	182.9	2.572	1248
Mouse 9 - Long USVs	0.2334	-573.2	70.2	66.68	1, 219	<0.0001	99.68	6.735	221
Mouse 10 - Long USVs	0.2031	-600.4	140.2	18.34	1, 72	<0.0001	102.4	12.86	74
Mouse 11 - Long USVs	0.1702	-356.9	47.87	55.6	1, 271	<0.0001	80.69	5.214	273
Mouse 12 - Long USVs	0.1103	-430.8	93.81	21.08	1, 170	<0.0001	77.32	8.878	172
Mouse 13 - LongUSVs	0.04513	-241.2	118.3	4.159	1, 88	<0.05	69.38	12.43	90
Mouse 14 - Long USVs	0.1586	-406.1	91.71	19.6	1, 104	<0.0001	80.95	8.906	106
Mouse 15 - Long USVs	0.05764	-180.4	64.46	7.829	1, 128	<0.01	54.23	6.53	130
Mouse 16 - Long USVs	0.1075	-368.0	72.15	26.02	1, 216	<0.0001	66.51	6.645	218
Mouse 17 - Long USVs	0.2444	-737.2	189.1	15.21	1, 47	<0.0005	118.4	17.76	49
Mouse 18 - Long USVs	0.04414	-165.9	49.71	11.27	1, 244	<0.001	49.8	5.201	246
Mouse 19 - Long USVs	0.2279	-589.2	51.77	129.6	1, 439	<0.0001	94.53	4.217	441
