# Supplementary material for: The temporal organization of mouse ultrasonic vocalizations
Source: PLoS One. 2018 Oct 30;13(10):e0199929. doi: 10.1371/journal.pone.0199929 (PMC6207298; doi:10.1371/journal.pone.0199929)
Supplement: S6 Table — (PDF) [file pone.0199929.s017.pdf]

Table S6. Multiple comparisons statistics for long USV probability as a function of following ISI duration (n = 19 mice)										
Data Set	Mean	Standard Error	D'Agostino & Pearson Normality Test		Repeated Measures One-Way ANOVA, with Geisser-Greenhouse Correction (50-75 ms Reference)			Repeated Measures One-Way ANOVA, with Geisser-Greenhouse Correction (500+ ms Reference)		
			P-Value (α = 0.003, Sidak Correction)	*K2*	*Adjusted P-Value (Dunnett)*	*q*	*DF*	*Adjusted P-Value (Dunnett)*	*q*	*DF*
40-50 ms	0.369	0.069	0.385	1.91	0.0001****	8.41	18	0.0976	2.83	18
50-75 ms	0.935	0.024	0.014	8.50	N/A	N/A	N/A	0.0001***	27.09	18
75-100 ms	0.825	0.046	0.037	6.58	0.4155	1.97	18	0.0001***	15.32	18
100-125 ms	0.568	0.048	0.131	4.06	0.0001***	6.27	18	0.0001***	9.49	18
125-150 ms	0.534	0.038	0.811	0.42	0.0001***	8.72	18	0.0001***	9.23	18
150-175 ms	0.464	0.028	0.106	4.49	0.0001***	11.99	18	0.0001***	11.43	18
175-200 ms	0.438	0.033	0.444	1.62	0.0001***	12.38	18	0.0001***	9.21	18
200-225 ms	0.474	0.033	0.790	0.47	0.0001***	11.18	18	0.0001***	10.17	18
225-250 ms	0.347	0.039	0.384	1.92	0.0001***	11.72	18	0.0003***	5.64	18
250-275 ms	0.296	0.048	0.829	0.37	0.0001***	11.51	18	0.0117*	3.89	18
275-300 ms	0.220	0.030	0.790	0.47	0.0001***	18.36	18	0.1106	2.77	18
300-325 ms	0.161	0.030	0.553	1.19	0.0001***	21.98	18	0.9993	0.48	18
325-350 ms	0.191	0.040	0.153	3.75	0.0001***	14.73	18	0.9723	0.97	18
350-400 ms	0.195	0.029	0.091	4.80	0.0001***	21.00	18	0.3516	2.08	18
400-450 ms	0.183	0.030	0.460	1.55	0.0001***	26.74	18	0.7504	1.46	18
450-500 ms	0.118	0.026	0.018	8.00	0.0001***	20.50	18	0.9612	1.02	18
500+ ms	0.145	0.020	0.269	2.63	0.0001***	27.09	18	N/A	N/A	N/A
