# Supplementary material for: The temporal organization of mouse ultrasonic vocalizations
Source: PLoS One. 2018 Oct 30;13(10):e0199929. doi: 10.1371/journal.pone.0199929 (PMC6207298; doi:10.1371/journal.pone.0199929)
Supplement: S7 Table — (PDF) [file pone.0199929.s018.pdf]

Table S7. Multiple comparisons statistics for long USV probability as a function of preceding ISI duration (n = 19 mice)

Data Set	Mean	Standard Error	D'Agostino & Pearson Normality Test		Repeated Measures One-Way ANOVA, with Geisser-Greenhouse Correction (50-75 ms Reference)			Repeated Measures One-Way ANOVA, with Geisser-Greenhouse Correction (500+ ms Reference)		
			P-Value (α = 0.003, Sidak Correction)	*K2*	*Adjusted P-Value (Dunnett)*	*q*	*DF*	*Adjusted P-Value (Dunnett)*	*q*	*DF*
40-50 ms	0.7048	0.0426	0.5684	1.130	0.0573	3.11	18	0.0001****	15.11	18
50-75 ms	0.8471	0.02987	0.0364	6.625	N/A	N/A	N/A	0.0001****	24.21	18
75-100 ms	0.8806	0.02874	0.1409	3.920	0.9809	0.93	18	0.0001****	26.46	18
100-125 ms	0.8365	0.0348	0.0431	6.290	0.9997	0.19	18	0.0001****	23.10	18
125-150 ms	0.7558	0.05055	0.4018	1.824	0.8483	1.30	18	0.0001****	13.39	18
150-175 ms	0.4761	0.05623	0.9892	0.022	0.001***	5.08	18	0.0001****	6.65	18
175-200 ms	0.4004	0.02832	0.0466	6.130	0.0001****	10.09	18	0.0001****	11.24	18
200-225 ms	0.3415	0.03915	0.0594	5.648	0.0001****	9.36	18	0.0001****	8.36	18
225-250 ms	0.3155	0.04581	0.7883	0.476	0.0001****	8.68	18	0.0004***	5.49	18
250-275 ms	0.3635	0.04102	0.7685	0.527	0.0001****	9.23	18	0.0001****	8.70	18
275-300 ms	0.2098	0.02826	0.3444	2.132	0.0001****	13.92	18	0.0042**	4.37	18
300-325 ms	0.2548	0.03923	0.4316	1.680	0.0001****	11.28	18	0.0008***	5.16	18
325-350 ms	0.3068	0.04421	0.5551	1.177	0.0001****	11.06	18	0.0003***	5.65	18
350-400 ms	0.2653	0.03043	0.8982	0.215	0.0001****	11.07	18	0.0001****	6.74	18
400-450 ms	0.1988	0.03892	0.0003	16.380	0.0001****	14.74	18	0.0823	2.92	18
450-500 ms	0.2998	0.0364	0.8015	0.442	0.0001****	10.36	18	0.0001****	7.95	18
500+ ms	0.05374	0.01763	0.1243	4.170	0.0001****	24.21	18	N/A	N/A	N/A
