# Supplementary material for: The temporal organization of mouse ultrasonic vocalizations
Source: PLoS One. 2018 Oct 30;13(10):e0199929. doi: 10.1371/journal.pone.0199929 (PMC6207298; doi:10.1371/journal.pone.0199929)
Supplement: S8 Table — (PDF) [file pone.0199929.s019.pdf]

Table S8. Summary statistics for series proportions (n = 19 mice)					
Data Set	Mean	Standard Error	Coefficient of Variation	D'Agostino & Pearson Normality Test	
				P-Value (α = 0.013, Sidak Correction)	*K2*
Proportion Isolate USVs	0.51	0.018	15.60%	0.9391	0.126
Proportion Groups	0.49	0.018	16.04%	0.9391	0.126
Proportion Isolate USVs	0.18	0.007	15.85%	0.0626	5.543
Proportion Bouts	0.82	0.007	3.47%	0.0626	5.543
Proportion USVs in Groups	0.79	0.018	10.09%	0.1884	3.833
Proportion USVs in Bouts	0.98	0.001	0.66%	0.2661	2.648
