# Supplementary material for: The temporal organization of mouse ultrasonic vocalizations
Source: PLoS One. 2018 Oct 30;13(10):e0199929. doi: 10.1371/journal.pone.0199929 (PMC6207298; doi:10.1371/journal.pone.0199929)
Supplement: S9 Table — (PDF) [file pone.0199929.s020.pdf]

**Table S9. Summary statistics for series durations (n = 19 mice)**					
Data Set	Mean (s)	Standard Error (s)	Coefficient of Variation	D'Agostino & Pearson Normality Test	
				P-Value (α = 0.013, Sidak Correction)	*K2*
Isolate USVs	0.037	0.003	30.72%	0.2138	3.085
Groups	0.421	0.021	21.96%	0.6269	0.934
Isolate USVs	0.020	0.001	18.97%	0.8743	0.269
Bouts	1.095	0.043	17.24%	0.9010	0.209
