# Supplementary material for: The temporal organization of mouse ultrasonic vocalizations
Source: PLoS One. 2018 Oct 30;13(10):e0199929. doi: 10.1371/journal.pone.0199929 (PMC6207298; doi:10.1371/journal.pone.0199929)
Supplement: S10 Table — (PDF) [file pone.0199929.s021.pdf]

Table S10. Summary statistics for number of USVs per series and groups per bout (n = 19 mice)

Data Set	Median	Mean	Standard Error	Coefficient of Variation	D'Agostino & Pearson Normality Test	
					P-Value (α = 0.009, Sidak Correction)	*K2*
USVs per Group	3.0	3.11	0.57	18.26%	<0.0001***	23.120
USVs per Bout	7.0	7.47	1.12	15.04%	0.1504	3.789
Groups per Bout	3.0	3.05	0.19	27.78%	0.0166	8.203
