# Supplementary material for: The temporal organization of mouse ultrasonic vocalizations
Source: PLoS One. 2018 Oct 30;13(10):e0199929. doi: 10.1371/journal.pone.0199929 (PMC6207298; doi:10.1371/journal.pone.0199929)
Supplement: S11 Table — (PDF) [file pone.0199929.s022.pdf]

Table S11. Summary statistics for raw transition probabilities (n = 19 mice)					
Data Set	Mean	Standard Error	Coefficient of Variation	D'Agostino & Pearson Normality Test	
				P-Value (α = 0.004, Sidak Correction)	*K2*
bS	0.64	0.02	14.97	0.9834	0.034
bL	0.36	0.02	26.86	0.9800	0.030
gS	0.52	0.03	23.48	0.8930	0.226
bL	0.48	0.03	25.54	0.8930	0.226
SS	0.59	0.01	10.61	0.9211	0.164
SL	0.41	0.01	15.48	0.9200	0.160
LL	0.63	0.02	15.88	0.9951	0.010
LS	0.37	0.02	27.61	0.9951	0.010
Lg	0.57	0.03	20.92	0.6728	0.793
Lb	0.43	0.03	28.13	0.6728	0.793
Sg	0.69	0.02	12.09	0.1640	3.616
Sb	0.31	0.02	27.28	0.1640	3.616
% Short USV Isolates	0.85	0.01	6.05%	0.4312	1.682
% Long USV Isolates	0.15	0.01	34.25%	0.4312	1.682
