# Supplementary material for: The temporal organization of mouse ultrasonic vocalizations
Source: PLoS One. 2018 Oct 30;13(10):e0199929. doi: 10.1371/journal.pone.0199929 (PMC6207298; doi:10.1371/journal.pone.0199929)
Supplement: S12 Table — (PDF) [file pone.0199929.s023.pdf]

Table S12. Summary statistics for Preference Scores (n = 19 mice)

Data Set	Mean	Standard Error	Coefficient of Variation	D'Agostino & Pearson Normality Test	
				P-Value (α = 0.004, Sidak Correction)	*K2*
bS	0.31	0.018	24.90	0.1396	3.938
bL	-0.31	0.020	24.90	0.1400	3.940
gS	0.07	0.022	143.21	0.0878	4.865
bL	-0.07	0.022	143.21	0.0878	4.865
SS	0.19	0.017	39.41	0.8403	0.348
SL	-0.19	0.020	39.41	0.8400	0.350
LL	0.26	0.019	31.55	0.5886	1.060
LS	-0.26	0.019	31.55	0.5886	1.060
Sg	0.17	0.026	65.47	0.4625	1.542
Lg	-0.17	0.026	65.47	0.4625	1.542
Sg	0.40	0.018	19.53	0.3392	2.163
Lb	-0.40	0.018	19.53	0.3392	2.163
