# Supplementary material for: The temporal organization of mouse ultrasonic vocalizations
Source: PLoS One. 2018 Oct 30;13(10):e0199929. doi: 10.1371/journal.pone.0199929 (PMC6207298; doi:10.1371/journal.pone.0199929)
Supplement: S13 Table — (PDF) [file pone.0199929.s024.pdf]

**Table S13. Multiple comparisons statistics for Preference Scores (n = 19 mice)**			
Comparison	Repeated Measures One-Way ANOVA, with Geisser-Greenhouse Correction		
	Adjusted P-Value (Sidak)	*t*	*DF*
bS vs. Sb	0.0002***	5.465	18
gS vs. Sg	<0.0001****	7.902	18
SL vs. LS	<0.0001****	5.855	18
bL vs. gL	<0.0001****	11.32	18
Lg vs. Lb	<0.0001****	10.92	18
