# Supplementary material for: The temporal organization of mouse ultrasonic vocalizations
Source: PLoS One. 2018 Oct 30;13(10):e0199929. doi: 10.1371/journal.pone.0199929 (PMC6207298; doi:10.1371/journal.pone.0199929)
Supplement: S14 Table — (PDF) [file pone.0199929.s025.pdf]

Table S14. Descriptive statistics for series onset and offset temporal regularities

Mouse	Median Durations (s)					Normalized Durations						Median Durations (s)					Normalized Durations					
	SSS	bSS	gSS	SSb	SSg	bSS / SSS	gSS / SSS	gSS / bSS	SSb / SSS	SSg / SSS	SSg / SSb	LLL	bLL	gLL	LLb	LLg	bLL / LLL	gLL / LLL	gLL / bLL	LLb / LLL	LLg / LLL	LLg / LLb
1	0.0256	0.0225	0.0246	0.0266	0.0266	0.88	0.96	1.09	1.04	1.04	1.00	0.1173	0.1034	0.1034	0.0993	0.1034	0.88	0.88	1.00	0.85	0.88	1.04
2	0.0236	0.0174	0.0215	0.0225	0.0236	0.74	0.91	1.24	0.96	1.00	1.05	0.1157	0.1024	0.0983	0.0942	0.1060	0.89	0.85	0.96	0.81	0.92	1.13
3	0.0277	0.0205	0.0297	0.0236	0.0297	0.74	1.07	1.45	0.85	1.07	1.26	0.1577	0.1423	0.1454	0.1178	0.1393	0.90	0.92	1.02	0.75	0.88	1.18
4	0.0277	0.0215	0.0266	0.0256	0.0277	0.78	0.96	1.24	0.93	1.00	1.08	0.1403	0.1219	0.1342	0.1085	0.1265	0.87	0.96	1.10	0.77	0.90	1.17
5	0.0277	0.0215	0.0246	0.0277	0.0277	0.78	0.89	1.14	1.00	1.00	1.00	0.1311	0.1096	0.1162	0.1039	0.1188	0.84	0.89	1.06	0.79	0.91	1.14
6	0.0246	0.0225	0.0261	0.0236	0.0277	0.92	1.06	1.16	0.96	1.12	1.17	0.1690	0.1577	0.1659	0.1106	0.1587	0.93	0.98	1.05	0.65	0.94	1.43
7	0.0236	0.0195	0.0225	0.0246	0.0277	0.83	0.96	1.16	1.04	1.17	1.12	0.1854	0.1603	0.1720	0.1219	0.1690	0.86	0.93	1.07	0.66	0.91	1.39
8	0.0287	0.0195	0.0277	0.0282	0.0307	0.68	0.96	1.42	0.98	1.07	1.09	0.1557	0.1413	0.1495	0.1086	0.1413	0.91	0.96	1.06	0.70	0.91	1.30
9	0.0297	0.0225	0.0256	0.0271	0.0287	0.76	0.86	1.14	0.91	0.97	1.06	0.1352	0.1162	0.1219	0.1137	0.1157	0.86	0.90	1.05	0.84	0.86	1.02
10	0.0225	0.0174	0.0205	0.0184	0.0205	0.77	0.91	1.18	0.82	0.91	1.11	0.1229	0.1060	0.1004	0.0947	0.1167	0.86	0.82	0.95	0.77	0.95	1.23
11	0.0277	0.0220	0.0256	0.0277	0.0277	0.80	0.93	1.16	1.00	1.00	1.00	0.1449	0.1111	0.1239	0.1034	0.1403	0.77	0.86	1.12	0.71	0.97	1.36
12	0.0302	0.0241	0.0307	0.0287	0.0307	0.80	1.02	1.28	0.95	1.02	1.07	0.1290	0.1178	0.1198	0.1014	0.1065	0.91	0.93	1.02	0.79	0.83	1.05
13	0.0277	0.0195	0.0287	0.0266	0.0287	0.70	1.04	1.47	0.96	1.04	1.08	0.1649	0.1475	0.1429	0.1147	0.1434	0.89	0.87	0.97	0.70	0.87	1.25
14	0.0230	0.0164	0.0195	0.0215	0.0195	0.71	0.84	1.19	0.93	0.84	0.90	0.1444	0.1065	0.1254	0.1050	0.1301	0.74	0.87	1.18	0.73	0.90	1.24
15	0.0307	0.0225	0.0225	0.0328	0.0307	0.73	0.73	1.00	1.07	1.00	0.94	0.1260	0.1019	0.0963	0.0973	0.1070	0.81	0.76	0.94	0.77	0.85	1.10
16	0.0256	0.0174	0.0236	0.0266	0.0246	0.68	0.92	1.35	1.04	0.96	0.92	0.1352	0.1183	0.1086	0.1055	0.1219	0.88	0.80	0.92	0.78	0.90	1.16
17	0.0277	0.0195	0.0256	0.0241	0.0266	0.70	0.93	1.32	0.87	0.96	1.11	0.1495	0.1188	0.1290	0.1173	0.1219	0.79	0.86	1.09	0.78	0.82	1.04
18	0.0287	0.0236	0.0266	0.0266	0.0277	0.82	0.93	1.13	0.93	0.96	1.04	0.1536	0.1372	0.1423	0.1188	0.1301	0.89	0.93	1.04	0.77	0.85	1.10
19	0.0184	0.0123	0.0164	0.0164	0.0154	0.67	0.89	1.33	0.89	0.83	0.94	0.1086	0.0860	0.0942	0.0768	0.0845	0.79	0.87	1.10	0.71	0.78	1.10
Total						17/19	3/19	11/19	4/19, 1/19	2/19	1/19						17/19	17/19	0/19	18/19	16/19	12/19

Significant Increase; Significant Decrease
