# Supplementary material for: The temporal organization of mouse ultrasonic vocalizations
Source: PLoS One. 2018 Oct 30;13(10):e0199929. doi: 10.1371/journal.pone.0199929 (PMC6207298; doi:10.1371/journal.pone.0199929)
Supplement: S15 Table — (PDF) [file pone.0199929.s026.pdf]

Table S15. Multiple comparisons statistics for series onset and offset temporal regularities, short USVs (one way Kruskal-Wallis test)

Mouse	SSS vs. bSS			SSS vs. gSS			bSS vs. gSS			SSS vs. SSb			SSS vs. SSg			SSb vs. SSg		
	Adjusted P-Value (Dunn's)	*n1 (USVs)*	*n2 (USVs)*	*Adjusted P-Value (Dunn's)*	*n1*	*n2*	*Adjusted P-Value (Dunn's)*	*n1*	*n2*	*Adjusted P-Value (Dunn's)*	*n1*	*n2*	*Adjusted P-Value (Dunn's)*	*n1*	*n2*	*Adjusted P-Value (Dunn's)*	*n1*	*n2*
1	0.0103*	515	191	0.5576	515	429	0.4387	191	429	>0.9999	515	199	>0.9999	515	453	>0.9999	199	453
2	0.0013**	974	184	0.2745	974	613	0.1222	184	613	>0.9999	974	226	>0.9999	974	660	>0.9999	226	660
3	0.0002***	424	123	>0.9999	424	260	<0.0001****	123	260	0.0418*	424	134	>0.9999	424	305	0.0031**	134	305
4	<0.0001****	787	201	0.248	787	327	0.0123*	201	327	0.0186*	787	241	>0.9999	787	322	0.5919	241	322
5	<0.0001****	639	208	0.0615	639	436	0.0817	208	436	>0.9999	639	190	>0.9999	639	426	>0.9999	190	426
6	>0.9999	140	85	>0.9999	140	114	0.7476	85	114	>0.9999	140	69	>0.9999	140	150	0.1918	69	150
7	0.0092**	179	75	>0.9999	179	102	0.15	75	102	>0.9999	179	74	>0.9999	179	121	>0.9999	74	121
8	0.2113	239	89	>0.9999	239	160	0.1718	89	160	>0.9999	239	84	0.3733	239	192	>0.9999	84	192
9	<0.0001****	744	192	0.0026**	744	392	0.0131*	192	392	0.6214	744	212	>0.9999	744	405	>0.9999	212	405
10	0.0001***	425	157	0.2939	425	392	0.0333*	157	392	0.0213*	425	176	0.9985	425	419	0.3865	176	419
11	0.0015**	325	114	0.0739	325	297	0.4335	114	297	>0.9999	325	150	>0.9999	325	328	>0.9999	150	328
12	<0.0001****	1344	276	>0.9999	1344	396	<0.0001****	276	396	0.0715	1344	304	>0.9999	1344	393	0.3659	304	393
13	<0.0001****	641	202	>0.9999	641	369	<0.0001****	202	369	0.3689	641	225	>0.9999	641	389	0.3502	225	389
14	<0.0001****	746	259	0.0082**	746	375	0.0113*	259	375	0.071	746	278	0.0008***	746	376	>0.9999	278	376
15	0.0032**	422	52	<0.0001****	422	225	>0.9999	52	225	<0.0001****	422	73	0.0002***	422	166	0.9287	73	166
16	<0.0001****	330	158	>0.9999	330	340	0.0006***	158	340	>0.9999	330	205	>0.9999	330	354	>0.9999	205	354
17	<0.0001****	552	212	0.0715	552	347	0.0021**	212	347	0.0093**	552	216	0.0447*	552	371	>0.9999	216	371
18	<0.0001****	625	227	>0.9999	625	292	0.0087**	227	292	0.3677	625	240	0.9581	625	346	>0.9999	240	346
19	0.0003***	250	185	>0.9999	250	451	0.0035**	185	451	>0.9999	250	187	0.3488	250	466	>0.9999	187	466
