# Supplementary material for: The temporal organization of mouse ultrasonic vocalizations
Source: PLoS One. 2018 Oct 30;13(10):e0199929. doi: 10.1371/journal.pone.0199929 (PMC6207298; doi:10.1371/journal.pone.0199929)
Supplement: S16 Table — (PDF) [file pone.0199929.s027.pdf]

Table S16. Multiple comparisons statistics for series onset and offset temporal regularities, long USVs (one-way Kruskal-Wallis test)																		
Mouse	LLL vs. bLL			LLL vs. gLL			bLLvs. gLL			LLL vs. LLb			LLL vs. LLg			LLb vs. LLg		
	Adjusted P-Value (Dunn's)	*n1 (USVs)*	*n2 (USVs)*	*Adjusted P-Value (Dunn's)*	*n1*	*n2*	*Adjusted P-Value (Dunn's)*	*n1*	*n2*	*Adjusted P-Value (Dunn's)*	*n1*	*n2*	*Adjusted P-Value (Dunn's)*	*n1*	*n2*	*Adjusted P-Value (Dunn's)*	*n1*	*n2*
1	0.0061**	826	141	<0.0001****	826	518	>0.9999	141	518	<0.0001****	826	127	<0.0001****	826	461	>0.9999	127	461
2	0.1924	566	77	<0.0001****	566	335	>0.9999	77	335	0.0002***	566	53	0.0024**	566	240	0.2098	53	240
3	0.0024**	1655	126	0.0003***	1655	588	>0.9999	126	588	<0.0001****	1655	84	<0.0001****	1655	510	0.0012**	84	510
4	<0.0001****	1166	157	0.0063**	1166	345	0.1607	157	345	<0.0001****	1166	134	0.0005**	1166	280	0.0001***	134	280
5	0.0011**	945	105	<0.0001****	945	522	>0.9999	105	522	<0.0001****	945	118	0.0015**	945	511	0.0004***	118	511
6	0.1234	1354	227	0.4483	1354	674	>0.9999	227	674	<0.0001****	1354	185	0.0249*	1354	576	<0.0001****	185	576
7	<0.0001****	1453	182	<0.0001****	1453	530	0.1016	182	530	<0.0001****	1453	162	<0.0001****	1453	503	<0.0001****	162	503
8	0.0177*	1897	209	0.1522	1897	695	0.8173	209	695	<0.0001****	1897	190	<0.0001****	1897	587	<0.0001****	190	587
9	<0.0001****	919	120	<0.0001****	919	293	>0.9999	120	293	<0.0001****	919	103	<0.0001****	919	239	0.2577	103	239
10	0.0396*	699	98	<0.0001****	699	511	0.5368	98	511	<0.0001****	699	86	0.0478*	699	453	<0.0001****	86	453
11	<0.0001****	1042	116	<0.0001****	1042	635	0.1329	116	635	<0.0001****	1042	92	0.1931	1042	535	<0.0001****	92	535
12	0.0154*	1479	114	0.004**	1479	185	>0.9999	114	185	<0.0001****	1479	105	<0.0001****	1479	158	>0.9999	105	158
13	0.0132*	1290	123	<0.0001****	1290	398	>0.9999	123	398	<0.0001****	1290	100	<0.0001****	1290	338	<0.0001****	100	338
14	0.0002***	712	45	<0.0001****	712	204	0.4699	45	204	<0.0001****	712	72	0.0277*	712	155	0.0134*	72	155
15	<0.0001****	1655	253	<0.0001****	1655	881	0.4447	253	881	>0.9999	1655	311	>0.9999	1655	844	0.9141	311	844
16	0.0017**	642	126	<0.0001****	642	489	>0.9999	126	489	<0.0001****	642	112	0.0009***	642	404	0.0085**	112	404
17	<0.0001****	699	64	<0.0001****	699	250	0.329	64	250	<0.0001****	699	66	<0.0001****	699	187	>0.9999	66	187
18	0.0001***	1391	167	0.0004***	1391	377	>0.9999	167	377	<0.0001****	1391	129	<0.0001****	1391	348	0.0018**	129	348
19	0.0094**	317	79	0.0106*	317	472	0.9649	79	472	<0.0001****	317	123	0.0013	317	384	0.1937	123	384
