# Supplementary material for: The temporal organization of mouse ultrasonic vocalizations
Source: PLoS One. 2018 Oct 30;13(10):e0199929. doi: 10.1371/journal.pone.0199929 (PMC6207298; doi:10.1371/journal.pone.0199929)
Supplement: S17 Table — (PDF) [file pone.0199929.s028.pdf]

Table S17. Summary statistics for series onset and offset normalized durations (n = 19 mice)						
Data Set	Median	Mean	Standard Error	Coefficient of Variation	D'Agostino & Pearson Normality Test	
					P-Value (α = 0.004, Sidak Correction)	*K2*
bSS / SSS	0.76	0.76	0.02	9.00%	0.3972	1.847
gSS / SSS	0.93	0.94	0.02	8.62%	0.2781	2.559
gSS / bSS	1.19	1.23	0.03	10.36%	0.7014	0.709
SSb / SSS	0.96	0.95	0.02	7.17%	0.8729	0.272
SSg / SSS	1.00	1.00	0.02	8.35%	0.6427	0.884
SSg / SSb	1.06	1.05	0.02	8.57%	0.7031	0.705
bLL / LLL	0.87	0.86	0.01	6.16%	0.2751	2.582
gLL / LLL	0.88	0.89	0.01	6.41%	0.7204	0.656
gLL / bLL	1.05	1.04	0.02	6.57%	0.9801	0.040
LLb / LLL	0.77	0.75	0.01	7.31%	0.8561	0.311
LLg / LLL	0.90	0.89	0.01	5.31%	0.6880	0.748
LLg / LLb	1.16	1.18	0.03	10.37%	0.4162	1.753
