# Supplementary material for: The temporal organization of mouse ultrasonic vocalizations
Source: PLoS One. 2018 Oct 30;13(10):e0199929. doi: 10.1371/journal.pone.0199929 (PMC6207298; doi:10.1371/journal.pone.0199929)
Supplement: S18 Table — (PDF) [file pone.0199929.s029.pdf]

Table S18. Descriptive statistics for adjacency-related temporal regularities

Mouse	Median Durations (s)				Normalized Durations						Median Durations (s)				Normalized Durations					
	SSS	SSL	LSS	LSL	SSL / SSS	LSS / SSS	LSL / SSS	LSL / SSL	LSL / LSS	LSS / SSL	LLL	LLS	SLL	SLS	LLS / LLL	SLL / LLL	SLS / LLL	SLS / LLS	SLS / SLL	SLL / LLS
1	0.0256	0.0287	0.0307	0.0307	1.12	1.20	1.20	1.07	1.00	1.07	0.1173	0.0983	0.0993	0.0911	0.84	0.85	0.78	0.93	0.92	1.01
2	0.0236	0.0266	0.0266	0.0256	1.13	1.13	1.09	0.96	0.96	1.00	0.1157	0.1055	0.0993	0.0911	0.91	0.86	0.79	0.86	0.92	0.94
3	0.0277	0.0348	0.0358	0.0348	1.26	1.30	1.26	1.00	0.97	1.03	0.1577	0.1290	0.1301	0.1091	0.82	0.82	0.69	0.85	0.84	1.01
4	0.0277	0.0297	0.0318	0.0348	1.07	1.15	1.26	1.17	1.10	1.07	0.1403	0.1178	0.1116	0.0983	0.84	0.80	0.70	0.83	0.88	0.95
5	0.0277	0.0307	0.0287	0.0287	1.11	1.04	1.04	0.93	1.00	0.93	0.1311	0.1126	0.1065	0.0922	0.86	0.81	0.70	0.82	0.87	0.95
6	0.0246	0.0307	0.0307	0.0338	1.25	1.25	1.38	1.10	1.10	1.00	0.1690	0.1372	0.1362	0.1034	0.81	0.81	0.61	0.75	0.76	0.99
7	0.0236	0.0287	0.0307	0.0318	1.22	1.30	1.35	1.11	1.03	1.07	0.1854	0.1403	0.1347	0.1004	0.76	0.73	0.54	0.72	0.75	0.96
8	0.0287	0.0338	0.0338	0.0328	1.18	1.18	1.14	0.97	0.97	1.00	0.1557	0.1362	0.1280	0.1106	0.87	0.82	0.71	0.81	0.86	0.94
9	0.0297	0.0328	0.0348	0.0358	1.10	1.17	1.21	1.09	1.03	1.06	0.1352	0.1167	0.1157	0.1024	0.86	0.86	0.76	0.88	0.89	0.99
10	0.0225	0.0236	0.0266	0.0277	1.05	1.18	1.23	1.17	1.04	1.13	0.1229	0.1106	0.1065	0.0973	0.90	0.87	0.79	0.88	0.91	0.96
11	0.0277	0.0307	0.0307	0.0287	1.11	1.11	1.04	0.93	0.93	1.00	0.1449	0.1239	0.1157	0.1014	0.86	0.80	0.70	0.82	0.88	0.93
12	0.0302	0.0338	0.0369	0.0389	1.12	1.22	1.29	1.15	1.06	1.09	0.1290	0.1126	0.1208	0.0993	0.87	0.94	0.77	0.88	0.82	1.07
13	0.0277	0.0338	0.0358	0.0348	1.22	1.30	1.26	1.03	0.97	1.06	0.1649	0.1336	0.1331	0.1106	0.81	0.81	0.67	0.83	0.83	1.00
14	0.0230	0.0256	0.0287	0.0271	1.11	1.24	1.18	1.06	0.95	1.12	0.1444	0.1178	0.1157	0.0922	0.82	0.80	0.64	0.78	0.80	0.98
15	0.0307	0.0338	0.0348	0.0358	1.10	1.13	1.17	1.06	1.03	1.03	0.1260	0.1116	0.1086	0.0973	0.89	0.86	0.77	0.87	0.90	0.97
16	0.0256	0.0287	0.0287	0.0266	1.12	1.12	1.04	0.93	0.93	1.00	0.1352	0.1224	0.1065	0.0983	0.91	0.79	0.73	0.80	0.92	0.87
17	0.0277	0.0297	0.0328	0.0358	1.07	1.19	1.30	1.21	1.09	1.10	0.1495	0.1198	0.1178	0.1029	0.80	0.79	0.69	0.86	0.87	0.98
18	0.0287	0.0318	0.0369	0.0369	1.11	1.29	1.29	1.16	1.00	1.16	0.1536	0.1249	0.1331	0.1086	0.81	0.87	0.71	0.87	0.82	1.07
19	0.0184	0.0164	0.0200	0.0154	0.89	1.08	0.83	0.94	0.77	1.22	0.1086	0.0942	0.0881	0.0727	0.87	0.81	0.67	0.77	0.83	0.93
Total					13/19	16/19	14/19	4/19	1/19	3/19					18/19	19/19	19/19	19/19	16/19	1/19, 1/19

Significant Increase; Significant Decrease
