# Supplementary material for: The temporal organization of mouse ultrasonic vocalizations
Source: PLoS One. 2018 Oct 30;13(10):e0199929. doi: 10.1371/journal.pone.0199929 (PMC6207298; doi:10.1371/journal.pone.0199929)
Supplement: S19 Table — (PDF) [file pone.0199929.s030.pdf]

Table S19. Multiple comparisons statistics for adjacency-related temporal regularities, short USVs (one-way Kruskal-Wallis test)

Mouse	SSL vs. SSS			LSS vs. SSS			LSL vs. SSS			LSL vs. SSL			LSL vs. LSS			LSS vs. SSL		
	Adjusted P-Value (Dunn's)	*n1 (USVs)*	*n2 (USVs)*	*Adjusted P-Value (Dunn's)*	*n1*	*n2*	*Adjusted P-Value (Dunn's)*	*n1*	*n2*	*Adjusted P-Value (Dunn's)*	*n1*	*n2*	*Adjusted P-Value (Dunn's)*	*n1*	*n2*	*Adjusted P-Value (Dunn's)*	*n1*	*n2*
1	0.0168*	515	305	<0.0001****	515	337	<0.0001****	515	355	0.1883	305	355	>0.9999	337	355	0.1051	305	337
2	0.0808	974	484	0.0479*	974	573	>0.9999	974	467	>0.9999	484	467	>0.9999	573	467	>0.9999	484	573
3	<0.0001****	424	245	<0.0001****	424	301	<0.0001****	424	391	>0.9999	245	391	>0.9999	301	391	>0.9999	245	301
4	0.0415*	787	321	<0.0001****	787	356	<0.0001****	787	341	0.0006***	321	341	0.6107	356	341	0.1246	321	356
5	0.0112*	639	434	>0.9999	639	406	>0.9999	639	451	0.0787	434	451	>0.9999	406	451	0.2709	434	406
6	0.0103*	140	111	0.0191*	140	131	<0.0001****	140	137	>0.9999	111	137	0.9943	131	137	>0.9999	111	131
7	0.1144	179	104	<0.0001****	179	122	<0.0001****	179	152	0.404	104	152	>0.9999	122	152	0.3914	104	122
8	0.0017**	239	164	0.0002***	239	191	0.0025**	239	314	>0.9999	164	314	>0.9999	191	314	>0.9999	164	191
9	0.0015**	744	472	<0.0001****	744	505	<0.0001****	744	423	0.1313	472	423	>0.9999	505	423	0.8953	472	505
10	>0.9999	425	221	<0.0001****	425	267	0.0011**	425	313	0.1366	221	313	0.9739	267	313	0.003**	221	267
11	>0.9999	325	162	0.1688	325	229	>0.9999	325	225	>0.9999	162	225	0.2211	229	225	>0.9999	162	229
12	<0.0001****	1344	600	<0.0001****	1344	625	<0.0001****	1344	491	<0.0001****	600	491	0.0562	625	491	<0.0001****	600	625
13	<0.0001****	641	309	<0.0001****	641	352	<0.0001****	641	322	>0.9999	309	322	0.4401	352	322	0.2919	309	352
14	0.0162*	746	332	<0.0001****	746	352	0.0018**	746	302	>0.9999	332	302	0.7466	352	302	0.1595	332	352
15	0.0114*	1655	607	0.0016**	1655	628	0.0003***	1655	346	>0.9999	607	346	>0.9999	628	346	>0.9999	607	628
16	0.2294	330	197	0.0782	330	258	>0.9999	330	230	0.3869	197	230	0.1681	258	230	>0.9999	197	258
17	0.0274*	552	221	<0.0001****	552	249	<0.0001****	552	155	0.0028**	221	155	0.2354	249	155	0.5581	221	249
18	0.0088**	625	351	<0.0001****	625	418	<0.0001****	625	381	<0.0001****	351	381	>0.9999	418	381	0.0019**	351	418
19	>0.9999	250	173	0.9985	250	190	0.1841	250	206	>0.9999	173	206	0.0049**	190	206	0.3549	173	190
