# Supplementary material for: The temporal organization of mouse ultrasonic vocalizations
Source: PLoS One. 2018 Oct 30;13(10):e0199929. doi: 10.1371/journal.pone.0199929 (PMC6207298; doi:10.1371/journal.pone.0199929)
Supplement: S20 Table — (PDF) [file pone.0199929.s031.pdf]

**Table S20. Multiple comparisons statistics for adjacency-related temporal regularities, long USVs (one-way Kruskal-Wallis test)**

| Mouse | LLS vs . LLL | | | SLL vs. LLL | | | SLS vs. LLL | | | SLS vs. LLS | | | SLS vs. SLL | | | SLL vs. LLS | | |
|---|---|---|---|---|---|---|---|---|---|---|---|---|---|---|---|---|---|---|
| | *Adjusted P-Value (Dunn's)* | *n1 (USVs)* | *n2 (USVs)* | *Adjusted P-Value (Dunn's)* | *n1* | *n2* | *Adjusted P-Value (Dunn's)* | *n1* | *n2* | *Adjusted P-Value (Dunn's)* | *n1* | *n2* | *Adjusted P-Value (Dunn's)* | *n1* | *n2* | *Adjusted P-Value (Dunn's)* | *n1* | *n2* |
| 1 | <0.0001**** | 826 | 480 | <0.0001**** | 826 | 409 | <0.0001**** | 826 | 324 | 0.0048** | 480 | 324 | 0.0014** | 409 | 324 | >0.9999 | 480 | 409 |
| 2 | <0.0001**** | 566 | 569 | <0.0001**** | 566 | 450 | <0.0001**** | 566 | 586 | <0.0001**** | 569 | 586 | 0.0086** | 450 | 586 | 0.1677 | 569 | 450 |
| 3 | <0.0001**** | 1655 | 611 | <0.0001**** | 1655 | 491 | <0.0001**** | 1655 | 252 | <0.0001**** | 611 | 252 | <0.0001**** | 491 | 252 | >0.9999 | 611 | 491 |
| 4 | <0.0001**** | 1166 | 497 | <0.0001**** | 1166 | 409 | <0.0001**** | 1166 | 333 | <0.0001**** | 497 | 333 | <0.0001**** | 409 | 333 | >0.9999 | 497 | 409 |
| 5 | <0.0001**** | 945 | 496 | <0.0001**** | 945 | 498 | <0.0001**** | 945 | 373 | <0.0001**** | 496 | 373 | <0.0001**** | 498 | 373 | 0.1165 | 496 | 498 |
| 6 | <0.0001**** | 1354 | 375 | <0.0001**** | 1354 | 235 | <0.0001**** | 1354 | 84 | 0.0112* | 375 | 84 | 0.0111* | 235 | 84 | >0.9999 | 375 | 235 |
| 7 | <0.0001**** | 1453 | 277 | <0.0001**** | 1453 | 230 | <0.0001**** | 1453 | 95 | <0.0001**** | 277 | 95 | 0.0005*** | 230 | 95 | >0.9999 | 277 | 230 |
| 8 | <0.0001**** | 1897 | 536 | <0.0001**** | 1897 | 409 | <0.0001**** | 1897 | 159 | <0.0001**** | 536 | 159 | 0.005** | 409 | 159 | 0.1214 | 536 | 409 |
| 9 | <0.0001**** | 919 | 609 | <0.0001**** | 919 | 538 | <0.0001**** | 919 | 451 | <0.0001**** | 609 | 451 | <0.0001**** | 538 | 451 | >0.9999 | 609 | 538 |
| 10 | <0.0001**** | 699 | 377 | <0.0001**** | 699 | 307 | <0.0001**** | 699 | 249 | <0.0001**** | 377 | 249 | 0.0724 | 307 | 249 | 0.2863 | 377 | 307 |
| 11 | <0.0001**** | 1042 | 393 | <0.0001**** | 1042 | 269 | <0.0001**** | 1042 | 152 | <0.0001**** | 393 | 152 | 0.0127* | 269 | 152 | 0.5701 | 393 | 269 |
| 12 | <0.0001**** | 1479 | 711 | 0.0004*** | 1479 | 675 | <0.0001**** | 1479 | 555 | <0.0001**** | 711 | 555 | <0.0001**** | 675 | 555 | 0.0017** | 711 | 675 |
| 13 | <0.0001**** | 1290 | 522 | <0.0001**** | 1290 | 439 | <0.0001**** | 1290 | 270 | <0.0001**** | 522 | 270 | <0.0001**** | 439 | 270 | >0.9999 | 522 | 439 |
| 14 | <0.0001**** | 712 | 362 | <0.0001**** | 712 | 340 | <0.0001**** | 712 | 349 | <0.0001**** | 362 | 349 | <0.0001**** | 340 | 349 | >0.9999 | 362 | 340 |
| 15 | <0.0001**** | 422 | 473 | <0.0001**** | 422 | 435 | <0.0001**** | 422 | 591 | <0.0001**** | 473 | 591 | 0.0003*** | 435 | 591 | >0.9999 | 473 | 435 |
| 16 | <0.0001**** | 642 | 342 | <0.0001**** | 642 | 243 | <0.0001**** | 642 | 190 | <0.0001**** | 342 | 190 | 0.2486 | 243 | 190 | 0.0361* | 342 | 243 |
| 17 | <0.0001**** | 699 | 303 | <0.0001**** | 699 | 242 | <0.0001**** | 699 | 214 | 0.0002*** | 303 | 214 | 0.0015** | 242 | 214 | >0.9999 | 303 | 242 |
| 18 | <0.0001**** | 1391 | 616 | <0.0001**** | 1391 | 549 | <0.0001**** | 1391 | 317 | <0.0001**** | 616 | 317 | <0.0001**** | 549 | 317 | 0.0814 | 616 | 549 |
| 19 | 0.1083 | 317 | 241 | 0.0027** | 317 | 197 | <0.0001**** | 317 | 172 | 0.0005*** | 241 | 172 | 0.0511 | 197 | 172 | >0.9999 | 241 | 197 |
