# Supplementary material for: The temporal organization of mouse ultrasonic vocalizations
Source: PLoS One. 2018 Oct 30;13(10):e0199929. doi: 10.1371/journal.pone.0199929 (PMC6207298; doi:10.1371/journal.pone.0199929)
Supplement: S21 Table — (PDF) [file pone.0199929.s032.pdf]

Table S21. Summary statistics for adjacency-related normalized durations (n = 19 mice)						
Data Set	Median	Mean	Standard Error	Coefficient of Variation	D'Agostino & Pearson Normality Test	
					P-Value (α = 0.005, Sidak Correction)	*K2*
SSL / SSS	1.11	1.12	0.02	7.39%	0.0480	6.074
LSS / SSS	1.18	1.19	0.02	6.54%	0.7005	0.712
LSL / SSS	1.21	1.19	0.03	11.24%	0.0567	5.741
LSL / SSL	1.06	1.06	0.02	8.86%	0.1419	3.905
LSL / LSS	1.00	1.00	0.02	7.67%	0.0062	10.170
LSS / SSL	1.06	1.06	0.02	6.43%	0.4796	1.469
LLS / LLL	0.86	0.85	0.01	4.82%	0.8549	0.314
SLL / LLL	0.81	0.83	0.01	5.40%	0.1884	3.339
SLS / LLL	0.70	0.71	0.01	9.18%	0.1510	3.782
SLS / LLS	0.83	0.83	0.01	6.26%	0.6805	0.770
SLS / SLL	0.87	0.86	0.01	6.01%	0.4834	1.454
SLL / LLS	0.97	0.97	0.01	4.89%	0.4611	1.548
