# Supplementary material for: The temporal organization of mouse ultrasonic vocalizations
Source: PLoS One. 2018 Oct 30;13(10):e0199929. doi: 10.1371/journal.pone.0199929 (PMC6207298; doi:10.1371/journal.pone.0199929)
Supplement: S22 Table — (PDF) [file pone.0199929.s033.pdf]

Table S22. Descriptive statistics for combined fine-scale temporal regularities

Mouse	Median Durations (s)				Normalized Durations		Median Durations (s)				Normalized Durations	
	bSS	bSL	SSb	LSb	bSL / bSS	LSb / SSb	bLL	bLS	LLb	SLb	bLS / bLL	SLb / LLb
1	0.0225	0.0210	0.0266	0.0307	0.93	1.15	0.1034	0.0835	0.0993	0.0809	0.81	0.81
2	0.0174	0.0225	0.0225	0.0297	1.29	1.32	0.1024	0.0952	0.0942	0.0824	0.93	0.88
3	0.0205	0.0282	0.0236	0.0389	1.37	1.65	0.1423	0.1301	0.1178	0.1014	0.91	0.86
4	0.0215	0.0246	0.0256	0.0312	1.14	1.22	0.1219	0.1045	0.1086	0.1014	0.86	0.93
5	0.0215	0.0241	0.0277	0.0348	1.12	1.26	0.1096	0.0988	0.1039	0.0922	0.90	0.89
6	0.0225	0.0215	0.0236	0.0328	0.95	1.39	0.1577	0.1162	0.1106	0.1178	0.74	1.06
7	0.0195	0.0210	0.0246	0.0317	1.08	1.29	0.1603	0.1229	0.1219	0.1034	0.77	0.85
8	0.0287	0.0338	0.0338	0.0328	1.18	0.97	0.1413	0.1260	0.1086	0.0840	0.89	0.77
9	0.0225	0.0236	0.0271	0.0369	1.05	1.36	0.1162	0.1050	0.1137	0.1014	0.90	0.89
10	0.0174	0.0200	0.0184	0.0277	1.15	1.50	0.1060	0.0886	0.0947	0.0645	0.84	0.68
11	0.0220	0.0256	0.0277	0.0358	1.16	1.30	0.1111	0.0952	0.1034	0.0978	0.86	0.95
12	0.0241	0.0277	0.0287	0.0394	1.15	1.37	0.1178	0.0952	0.1014	0.0952	0.81	0.94
13	0.0195	0.0225	0.0266	0.0379	1.16	1.42	0.1475	0.1075	0.1147	0.1096	0.73	0.96
14	0.0164	0.0195	0.0215	0.0297	1.19	1.38	0.1065	0.0911	0.1050	0.0804	0.86	0.77
15	0.0225	0.0266	0.0328	0.0440	1.18	1.34	0.1019	0.0942	0.0973	0.1075	0.92	1.11
16	0.0174	0.0246	0.0266	0.0317	1.41	1.19	0.1183	0.0947	0.1055	0.0850	0.80	0.81
17	0.0277	0.0297	0.0328	0.0358	1.07	1.09	0.1188	0.0901	0.1173	0.0783	0.76	0.67
18	0.0236	0.0277	0.0266	0.0348	1.17	1.31	0.1372	0.0973	0.1188	0.1091	0.71	0.92
19	0.0123	0.0154	0.0164	0.0205	1.25	1.25	0.0860	0.0748	0.0768	0.0717	0.87	0.93
Total					5/19	17/19					12/19	9/19

Significant Increase; Significant Decrease
