# Supplementary material for: The temporal organization of mouse ultrasonic vocalizations
Source: PLoS One. 2018 Oct 30;13(10):e0199929. doi: 10.1371/journal.pone.0199929 (PMC6207298; doi:10.1371/journal.pone.0199929)
Supplement: S23 Table — (PDF) [file pone.0199929.s034.pdf]

Table S23. Multiple comparison statistics for combined fine-scale temporal regularities (one-way Kruskal-Wallis test)

Mouse	bSS vs. bSL			SSb vs. LSb			bLL vs. bLS			LLb vs. SLb		
	Adjusted P-Value (Dunn's)	*n1 (USVs)*	*n2 (USVs)*	*Adjusted P-Value (Dunn's)*	*n1*	*n2*	*Adjusted P-Value (Dunn's)*	*n1*	*n2*	*Adjusted P-Value (Dunn's)*	*n1*	*n2*
1	>0.9999	191	106	**0.0002******	199	182	**<0.0001*******	141	108	**0.0013*****	127	77
2	0.0562	184	93	**<0.0001*******	226	133	0.1227	77	96	0.3194	53	70
3	**0.0064*****	123	88	**<0.0001*******	134	117	0.3649	126	61	**0.0436***	84	41
4	0.5226	201	115	**0.0006******	241	180	**0.0062*****	157	102	0.616	134	53
5	0.3095	208	88	**0.0025*****	190	127	0.069	105	74	**0.0164***	118	73
6	>0.9999	85	57	**<0.0001*******	69	165	**0.0032*****	227	44	>0.9999	185	28
7	0.2321	75	72	**0.0129***	74	103	**0.0007******	182	52	0.2954	162	26
8	**0.0006******	239	164	0.5109	191	314	**0.0008******	209	50	**0.0121***	190	35
9	0.3008	192	125	**<0.0001*******	212	161	**0.0253***	120	90	0.1114	103	74
10	>0.9999	157	82	**<0.0001*******	176	159	**0.0039*****	98	72	**0.0031*****	86	34
11	0.4643	114	85	**<0.0001*******	150	143	0.0897	116	65	0.167	92	26
12	**0.0116***	276	157	**<0.0001*******	304	196	**0.001*****	114	102	>0.9999	105	72
13	0.4579	202	74	**<0.0001*******	225	160	**<0.0001*******	123	65	>0.9999	100	31
14	**0.0115***	259	93	**<0.0001*******	278	132	0.2534	45	63	**0.0052*****	72	52
15	0.481	253	85	**<0.0001*******	311	117	0.7082	52	59	>0.9999	73	49
16	**0.0057*****	158	70	**0.0007******	205	162	**0.0042*****	126	76	**0.0494***	112	53
17	**0.0091*****	552	221	0.0785	249	155	**0.0155***	64	61	**0.0009******	66	32
18	0.1084	227	125	**<0.0001*******	240	181	**<0.0001*******	167	117	0.6288	129	72
19	0.1727	185	107	**0.0322***	187	159	**0.0336***	79	98	0.1305	123	99
