# Supplementary material for: The temporal organization of mouse ultrasonic vocalizations
Source: PLoS One. 2018 Oct 30;13(10):e0199929. doi: 10.1371/journal.pone.0199929 (PMC6207298; doi:10.1371/journal.pone.0199929)
Supplement: S24 Table — (PDF) [file pone.0199929.s035.pdf]

Table S24. Summary statistics for combined fine-scale normalized durations (n = 19 mice)

Data Set	Median	Mean	Standard Error	Coefficient of Variation	D'Agostino & Pearson Normality Test	
					P-Value (α = 0.012, Sidak Correction)	*K2*
bSL / bSS	1.16	1.16	0.03	10.36%	0.6584	0.836
LSb / SSb	1.31	1.30	0.03	11.44%	0.4053	1.806
bLS / bLL	0.86	0.84	0.02	8.20%	0.2973	2.426
SLb / LLb	0.89	0.88	0.03	12.77%	0.8816	0.252
