# Supplementary material for: The temporal organization of mouse ultrasonic vocalizations
Source: PLoS One. 2018 Oct 30;13(10):e0199929. doi: 10.1371/journal.pone.0199929 (PMC6207298; doi:10.1371/journal.pone.0199929)
Supplement: S25 Table — (PDF) [file pone.0199929.s036.pdf]

Table S25. Summary statistics for pup vocal development (n = 11, *n = 9 mice)

Data Set		Mean	Standard Error	Coefficient of Variation	D'Agostino & Pearson Normality Test	
					P-Value (α = 0.009, Sidak Correction)	*K2*
USV Duration (s)	P0-P3	0.061	0.004	21.93%	0.3238	2.255
	P4-P5	0.048	0.003	20.86%	0.1323	4.045
	P6-P7	0.037	0.003	28.70%	0.9699	0.061
	P8-P9	0.031	0.002	19.19%	0.2506	2.768
	P10-P11	0.031	0.002	23.91%	0.1924	3.297
	P12-P16	0.021	0.002	37.79%	0.2648	2.657
Median IVI Duration* (s)	P0-P3	0.242	0.005	5.97%	0.0740	5.206
	P4-P5	0.169	0.006	10.58%	0.5559	1.174
	P6-P7	0.152	0.003	5.96%	0.5038	1.371
	P8-P9	0.143	0.002	4.99%	0.2653	2.654
	P10-P11	0.133	0.001	3.25%	0.0256	7.334
	P12-P16	0.126	0.003	7.02%	0.8187	0.400
Weighted Frequency (Hz)	P0-P3	74095	643.4	2.88%	0.4489	1.602
	P4-P5	76412	932.8	4.05%	0.1559	3.717
	P6-P7	79465	1591	6.64%	0.7257	0.641
	P8-P9	82639	1193	4.79%	0.0011	13.580
	P10-P11	82414	947.6	3.81%	0.5698	1.125
	P12-P16	81496	836.9	3.41%	0.8327	0.366
