# Supplementary material for: The temporal organization of mouse ultrasonic vocalizations
Source: PLoS One. 2018 Oct 30;13(10):e0199929. doi: 10.1371/journal.pone.0199929 (PMC6207298; doi:10.1371/journal.pone.0199929)
Supplement: S26 Table — (PDF) [file pone.0199929.s037.pdf]

Table S26. Summary statistics for adult vocal development (n = 19 mice)

Data Set		Mean	Standard Error	Coefficient of Variation	D'Agostino & Pearson Normality Test	
					P-Value (a = 0.013, Sidak Correction)	*K2*
Short USV Duration (s)	P17-P34	0.021	0.001	27.75%	0.556	1.175
	P35-P49	0.025	0.001	17.34%	0.192	3.304
	P50-P65	0.027	0.001	14.65%	0.200	3.222
	P66-P95	0.027	0.001	14.59%	0.054	5.854
Long USV Duration (s)	P17-P34	0.087	0.005	24.09%	0.310	2.342
	P35-P49	0.108	0.004	17.35%	0.959	0.085
	P50-P65	0.116	0.005	17.34%	0.377	1.951
	P66-P95	0.117	0.004	15.21%	0.875	0.267
Short USV Variance (ms)	P17-P34	0.158	0.017	46.42%	0.451	1.593
	P35-P49	0.197	0.011	24.29%	0.378	1.947
	P50-P65	0.196	0.010	22.89%	0.174	3.496
	P66-P95	0.181	0.011	25.74%	0.077	5.142
Long USV Variance (ms)	P17-P34	1.660	0.102	26.66%	0.299	2.412
	P35-P49	2.159	0.093	18.72%	0.112	4.371
	P50-P95	2.332	0.115	21.50%	0.524	1.293
	P66-P85	2.628	0.123	20.37%	0.379	1.938
Median IVI Duration (s)	P17-P34	0.087	0.002	9.26%	0.854	0.316
	P35-P49	0.086	0.001	5.88%	0.439	1.645
	P50-P65	0.082	0.001	6.91%	0.557	1.171
	P66-P95	0.080	0.001	5.44%	0.278	2.563
Short USV Weighted Frequency (Hz)	P17-P34	80960	392	2.11%	0.388	1.894
	P35-P49	80269	289	1.57%	0.156	3.720
	P50-P65	79075	486	2.68%	0.391	1.880
	P66-P95	78661	526	2.92%	0.882	0.251
Long USV Weighted Frequency (Hz)	P17-P34	76454	574	3.28%	0.030	6.995
	P35-P49	74887	460	2.68%	0.521	1.303
	P50-P65	73353	719	4.27%	0.774	0.513
	P66-P95	72782	659	3.94%	0.311	2.334
Proportion Long USV	P17-P34	0.398	0.016	17.20%	0.522	1.301
	P35-P49	0.523	0.020	16.52%	0.753	0.568
	P50-P65	0.552	0.020	15.91%	0.739	0.605
	P66-P95	0.529	0.023	19.14%	0.674	0.790
Ashman's D Score	P17-P34	2.17	0.08	16.15%	0.056	5.757
	P35-P49	2.41	0.08	14.65%	0.095	4.715
	P50-P65	2.48	0.08	13.64%	0.402	1.823
	P66-P95	2.41	0.05	9.67%	0.752	0.571
