# Supplementary material for: The temporal organization of mouse ultrasonic vocalizations
Source: PLoS One. 2018 Oct 30;13(10):e0199929. doi: 10.1371/journal.pone.0199929 (PMC6207298; doi:10.1371/journal.pone.0199929)
Supplement: S27 Table — (PDF) [file pone.0199929.s038.pdf]

Table S27. Statistics for pup age group comparisons

Repeated Measures One-Way ANOVA, with Geisser-Greenhouse Correction		Age Group Comparison														
		P0-P3 vs.					*P4-P5 vs.*				*P6-P7 vs.*			*P8-P9 vs.*		*P10-P11 vs.*
		P4-P5	*P6-P7*	*P8-P9*	*P10-P11*	*P12-P16*	*P6-P7*	*P8-P9*	*P10-P11*	*P12-P16*	*P8-P9*	*P10-P11*	*P12-P16*	*P10-P11*	*P12-P16*	*P12-P16*
USV Duration	Adj. P-Value (Dunnett)	0.297	**0.0003*** **	**0.0002*** **	**<0.0001******	**<0.0001******	0.2568	**0.0103***	**0.0053** **	**0.0009*** **	0.1699	0.5689	**0.002** **	>0.9999	**0.0063** **	**0.0021** **
	q	3.18	10.37	10.71	13.55	15.60	3.33	6.40	7.06	8.89	3.75	2.38	8.08	0.26	6.88	7.99
	DF	10	10	10	10	10	10	10	10	10	10	10	10	10	10	10
Median IVI Duration	Adj. P-Value (Dunnett)	**<0.0001******	**<0.0001******	**<0.0001******	**<0.0001******	**<0.0001******	0.3258	0.0865	**0.004** **	**0.0052** **	**0.0399***	**0.0052** **	**0.0017** **	**0.0269***	**0.0005*** **	0.2557
	q	17.81	27.13	26.95	29.19	33.14	3.13	4.59	8.07	7.74	5.41	7.75	9.19	5.84	11.00	3.41
	DF	8	8	8	8	8	8	8	8	8	8	8	8	8	8	8
Weighted Frequency	Adj. P-Value (Dunnett)	0.3479	0.1165	**0.001*** **	**0.0005*** **	**<0.0001******	0.5884	**0.0124***	**0.0026** **	**0.0105***	0.0903	0.1269	0.8495	0.9997	0.9456	0.9781
	q	3.003	4.118	8.85	9.491	12.13	2.333	6.221	7.783	6.381	4.36	4.037	1.628	0.3779	1.23	0.9854
	DF	10	10	10	10	10	10	10	10	10	10	10	10	10	10	10
