# Supplementary material for: The temporal organization of mouse ultrasonic vocalizations
Source: PLoS One. 2018 Oct 30;13(10):e0199929. doi: 10.1371/journal.pone.0199929 (PMC6207298; doi:10.1371/journal.pone.0199929)
Supplement: S28 Table — (PDF) [file pone.0199929.s039.pdf]

Table S28. Statistics for adult age group comparisons

Repeated Measures One-Way ANOVA, with Geisser-Greenhouse Correction		Age Group Comparison					
		P17-P34 vs.			*P35-P49 vs.*		*P50-P65 vs.*
		P35-49	*P50-65*	*P66-95*	*P50-P65*	*P66-95*	*P66-P95*
Short USV Duration	Adj. P-Value (Dunnett)	**0.0072****	**0.0007*****	**0.0091****	**0.0485***	0.5116	0.4328
	q	5.310	6.803	5.159	4.018	1.989	2.185
	DF	18	18	18	18	18	18
Long USV Duration	Adj. P-Value (Dunnett)	**<0.0001******	**<0.0001******	**<0.0001******	**0.0186***	**0.0462***	0.7891
	q	8.153	9.933	9.02	4.679	4.053	1.316
	DF	18	18	18	18	18	18
Short USV Variance	Adj. P-Value (Dunnett)	0.1315	0.2421	0.7123	0.9987	0.2754	0.0766
	q	3.275	2.762	1.511	0.2149	2.644	3.688
	DF	18	18	18	18	18	18
Long USV Variance	Adj. P-Value (Dunnett)	**0.0004*****	**<0.0001******	**<0.0001******	0.1567	**0.0008*****	**0.0003*****
	q	7.248	9.061	12.13	3.134	6.713	7.364
	DF	18	18	18	18	18	18
Median IVI Duration	Adj. P-Value (Dunnett)	0.9985	0.2663	0.0638	**0.001*****	**0.0003*****	0.1666
	q	0.227	2.675	3.822	6.604	7.348	3.083
	DF	18	18	18	18	18	18
Short USV Weighted Frequency	Adj. P-Value (Dunnett)	0.2851	**0.0318***	**0.0047****	**0.0085****	**0.0015****	0.5703
	q	2.611	4.314	5.59	5.203	6.329	1.849
	DF	18	18	18	18	18	18
Long USV Weighted Frequency	Adj. P-Value (Dunnett)	**0.0302***	**0.0006*****	**<0.0001******	**0.0166***	**0.003****	0.4009
	q	4.349	6.911	8.151	4.757	5.89	2.269
	DF	18	18	18	18	18	18
Proportion Long USVs	Adj. P-Value (Dunnett)	**<0.0001******	**<0.0001******	**0.0029****	0.1093	0.9949	0.4943
	q	8.253	8.969	5.909	3.419	0.3403	2.031
	DF	18	18	18	18	18	18
Ashman's D Score	Adj. P-Value (Dunnett)	**0.0133***	**0.0013****	**0.0408***	0.2014	0.9998	0.4108
	q	4.905	6.42	4.141	2.923	0.1055	2.243
	DF	18	18	18	18	18	18
