# Supplementary material for: The temporal organization of mouse ultrasonic vocalizations
Source: PLoS One. 2018 Oct 30;13(10):e0199929. doi: 10.1371/journal.pone.0199929 (PMC6207298; doi:10.1371/journal.pone.0199929)
Supplement: S29 Table — (PDF) [file pone.0199929.s040.pdf]

Table S29. Summary statistics for USV group classifications (100 iterations of 250 random groups each)												
**Number of Groups**	**Data Set**											
	Pup				Adult Female				Adult Male			
	Mean (%)	*StDev (%)*	*95% Confidence Interval (%)*		*Mean*	*StDev*	*95% Confidence Interval*		*Mean*	*StDev*	*95% Confidence Interval*	
1	87.5	1.9	87.1	87.9	83.2	1.8	82.8	83.6	67.5	3.3	66.8	68.1
2	87.7	2.6	87.2	88.3	83.7	3.1	83.1	84.4	68.2	4.3	67.3	69.0
3	96.5	2.0	96.1	96.9	92.5	2.7	92.0	93.1	78.6	4.3	77.7	79.5
4	96.2	2.4	95.8	96.7	93.3	2.8	92.8	93.9	78.6	4.3	77.7	79.5
5	98.8	1.6	98.5	99.1	96.8	2.6	96.3	97.3	84.8	5.0	83.8	85.7
6	98.9	1.8	98.6	99.3	96.2	2.8	95.7	96.8	85.6	5.6	84.5	86.7
7	99.7	0.9	99.5	99.9	98.5	1.8	98.2	98.9	90.8	5.3	89.7	91.8
8	99.7	0.9	99.6	99.9	99.1	1.7	98.8	99.5	89.5	5.8	88.4	90.7
9	99.8	0.8	99.7	100.0	99.3	1.7	98.9	99.6	92.4	5.2	91.4	93.4
10	100.0	0.0	100.0	100.0	99.5	1.7	99.1	99.8	93.3	4.7	92.4	94.3
11	100.0	0.0	100.0	100.0	99.6	1.3	99.3	99.9	94.8	4.5	93.9	95.7
12	100.0	0.5	99.9	100.0	99.7	1.5	99.4	99.9	95.6	4.2	94.8	96.4
