# Supplementary material for: The temporal organization of mouse ultrasonic vocalizations
Source: PLoS One. 2018 Oct 30;13(10):e0199929. doi: 10.1371/journal.pone.0199929 (PMC6207298; doi:10.1371/journal.pone.0199929)
Supplement: S30 Table — (PDF) [file pone.0199929.s041.pdf]

Table S30. Pitch jumps per USV-USV duration linear fit statistics (n = 19 mice)

Data Set	R^2 (Pearson)	Slope (pitch jumps/sec)	Standard Error (pitch jumps/sec)	Slope T-Statistic	Slope P-Value	Y-Intercept (pitch jumps)	Standard Error (pitch jumps)	Intercept T-Statistic	Intercept P-Value	n (USVs)
Mouse 1	0.426	21.0	0.237	88.5	<0.0001	-0.16	0.02	-8.2	<0.0001	10567
Mouse 2	0.484	22.8	0.222	102.6	<0.0001	-0.16	0.02	-10.0	<0.0001	11243
Mouse 3	0.476	23.1	0.242	95.7	<0.0001	-0.04	0.03	-1.6	<0.0001	10104
Mouse 4	0.413	17.3	0.220	78.9	<0.0001	-0.08	0.02	-4.0	<0.0001	8847
Mouse 5	0.456	21.9	0.233	94.0	<0.0001	-0.01	0.02	-0.6	<0.0001	10530
Mouse 6	0.544	26.2	0.273	96.2	<0.0001	-0.21	0.04	-5.5	<0.0001	7742
Mouse 7	0.530	25.7	0.294	87.3	<0.0001	-0.29	0.04	-6.7	<0.0001	6763
Mouse 8	0.422	21.3	0.262	81.3	<0.0001	-0.19	0.03	-6.0	<0.0001	9054
Mouse 9	0.510	25.9	0.260	99.5	<0.0001	-0.23	0.02	-9.7	<0.0001	9526
Mouse 10	0.558	28.9	0.261	110.4	<0.0001	-0.18	0.02	-8.1	<0.0001	9671
Mouse 11	0.517	30.2	0.300	100.7	<0.0001	-0.24	0.03	-7.7	<0.0001	9503
Mouse 12	0.517	27.2	0.253	107.3	<0.0001	-0.49	0.02	-21.5	<0.0001	10746
Mouse 13	0.508	20.9	0.215	97.0	<0.0001	-0.16	0.02	-6.9	<0.0001	9100
Mouse 14	0.604	28.9	0.268	107.6	<0.0001	-0.28	0.02	-12.1	<0.0001	7606
Mouse 15	0.455	23.6	0.239	98.5	<0.0001	-0.14	0.02	-8.5	<0.0001	11622
Mouse 16	0.477	23.8	0.260	91.6	<0.0001	-0.19	0.02	-8.3	<0.0001	9206
Mouse 17	0.557	26.8	0.283	94.7	<0.0001	-0.24	0.03	-9.8	<0.0001	7136
Mouse 18	0.526	25.2	0.242	104.0	<0.0001	-0.32	0.03	-12.3	<0.0001	9759
Mouse 19	0.505	27.6	0.268	103.1	<0.0001	-0.20	0.02	-10.7	<0.0001	10422
