# Supplementary material for: The temporal organization of mouse ultrasonic vocalizations
Source: PLoS One. 2018 Oct 30;13(10):e0199929. doi: 10.1371/journal.pone.0199929 (PMC6207298; doi:10.1371/journal.pone.0199929)
Supplement: S31 Table — (PDF) [file pone.0199929.s042.pdf]

Table S31. Summary statistics for pitch jump-USV duration linear regressions (n = 19 mice)

Data Set	Mean	Standard Error	Coefficient of Variation	D'Agostino & Pearson Normality Test	
				P-Value (a = 0.013, Sidak Correction)	*K2*
Slope	24.7	0.765	13.52%	0.8226	0.391
Intercept	-0.20	0.02	52.87%	0.0652	5.461
R^2 (Pearson)	0.499	0.012	10.14%	0.9925	0.015
