# Supplementary material for: The temporal organization of mouse ultrasonic vocalizations
Source: PLoS One. 2018 Oct 30;13(10):e0199929. doi: 10.1371/journal.pone.0199929 (PMC6207298; doi:10.1371/journal.pone.0199929)
Supplement: S32 Table — (PDF) [file pone.0199929.s043.pdf]

**Table S32. Summary statistics and multiple comparisons for group durations**						
Data Set	Mean (log s)	Median (log s)	Coefficient of Variation	D'Agostino & Pearson Normality Test		One-Way Kruskal-Wallis Test
				P-Value (α = 0.017, Sidak Correction)	*K2*	*Adjusted P-Value (Dunn's)*
Pup (n = 3,434)	-0.330	-0.391	116.94%	<0.0001	246.00	0.4043 (vs. Male), <0.0001**** (vs. Female)
Adult Female (n = 706)	-0.336	-0.351	111.78%	<0.0001	1159.00	<0.0001*** (vs. Male and Pup)
Adult Male (n = 34,163)	-0.553	-0.607	71.95%	<0.0001	47.54	0.4043 (vs.Pup), <0.0001***(vs. Female)
